# A mixed methods expert opinion study on the optimal content and format for an occupational therapy intervention to improve sleep in schizophrenia spectrum disorders

**DOI:** 10.1371/journal.pone.0269453

**Published:** 2022-06-06

**Authors:** Sophie M. Faulkner, Richard J. Drake, Margaret Ogden, Maria Gardani, Penny E. Bee

**Affiliations:** 1 Faculty of Biology, Medicine and Health, The University of Manchester, Manchester, United Kingdom; 2 Greater Manchester Mental Health NHS Foundation Trust, Manchester, United Kingdom; 3 School of Health in Social Sciences, University of Edinburgh, Edinburgh, United Kingdom; Medical University of Vienna, AUSTRIA

## Abstract

**Introduction:**

People with schizophrenia spectrum disorder diagnoses commonly have poor sleep, which predicts various negative outcomes. The problems are diverse, including substantial circadian dysregulation, sleep-wake timing issues, hypersomnia (excessive sleep), and more classic insomnia.

**Methods:**

This paper reports on a mixed methods expert opinion study based on the principles of Delphi methodology. The study examines and explores opinion on the optimal contents and format for an occupational therapy intervention to improve poor sleep in this population. Views of clinical and academic topic experts (n = 56), were elicited, examined and explored in three rounds, views from previous rounds being presented back to participants in subsequent rounds. Participants with relevant personal experience (n = 26) then rated and commented on suggestions, with a focus on acceptability. Descriptive statistics and graphs of ratings were triangulated with qualitative content analysis of free-text.

**Results:**

Participants emphasised the central importance of intervention personalisation, although the manner and extent of personalisation suggested varied. Many components and domains were acknowledged as important, with the challenge being how to keep such an intervention simple, brief, and feasible for end-users, for sustainable implementation. The strongest consensus was to address evening routine, daytime activity, and environmental interventions. Relaxation, mindfulness, thermoregulation, sensory factors, and cognitive or psychological approaches were rated as less important. There was disagreement on whether to include time in bed restriction, and how to address napping, as well as how far to address medication timing. Clinicians and researchers advocated some version of stimulus control, but participants with personal experience reported low levels of acceptability for this, describing entirely negative experiences using ‘the 15-minute rule’ (part of stimulus control).

**Conclusion:**

These results are informative for clinicians treating sleep problems in people with schizophrenia and related conditions, as well as for decision makers considering the potential contribution of the profession of occupational therapy toward sleep treatment.

## Introduction

People with schizophrenia spectrum diagnoses (SzSD) often have chronic sleep problems which persist outside of relapse [1, 2]. Poor sleep is associated with, and may cause, negative physical and mental health outcomes in this population, including, but not limited to, the exacerbation of psychotic symptoms [3], poorer cardiometabolic health [4], compromised cognition [5] and functioning [6].

The type of sleep problems experienced by people with SzSD are diverse [7], and often differ from those in the general population, or in insomnia without comorbidity (previously described as ‘primary insomnia’ or ‘psychophysiological insomnia’). Whilst many adults with SzSD do experience classic insomnia symptoms such as short sleep duration, difficulty with sleep onset and maintenance and early awakening, phenomenologically different problems also occur. Insomnia with normal sleep length, and insomnia with hypersomnia are also common (37.8%, 17.6% respectively) [8]. Circadian dysregulation problems (often identified via sleep timing abnormalities) are also prevalent, including free-running (non-24hr) rhythms [9–11], which are usually considered rare in individuals without visual impairment [12]. Of note, many studies of sleep problems in SzSD do not distinguish between insomnia and circadian dysregulation, and typically describe all sleep problems as insomnia.

Some authors advocate the use of hypnotics and sedative antipsychotics to improve sleep in SzSD, and summarise positive effects [13, 14]. However there is no good evidence of long term sleep improvement, and there is evidence of long term harms associated with hypnotics [15]. Melatonin or melatonin agonists show promise in this group but there has been as yet only pilot work [16, 17] except in antipsychotic naïve or treatment withdrawn patients [18]. People with SzSD tend to see drug treatments for sleep problems as less acceptable [19, 20], and value the opportunity to learn skills to manage their sleep non-pharmacologically [6].

There is clear evidence that cognitive behavioural therapy for insomnia (CBTi) can be effective in people with insomnia with and without comorbidity [21–24], and should be used in preference of drug treatments for sleep because of its lower adverse effects [25, 26]. However, CBTi may not effectively target circadian rhythm sleep problems. CBTi in combination with morning light therapy has shown positive effects on sleep in young people with delayed sleep-wake phase disorder [27, 28], but benefit might have been attributable to light therapy alone, as addition of CBTi showed no evidence of additional benefit [28]. For sleep problems more related to circadian dysregulation than insomnia processes, light therapy with sleep-wake scheduling has good theoretical backing, and support from basic research [29, 30]. Light therapy has shown promising results in meta-analysis of clinical trials, despite some issues identified with sub-optimal treatment protocols [31, 32], and light therapy in combination with CBTi has been successfully trialled in inpatients with psychotic and bipolar disorders [33].

Through having been designed to target insomnia rather than circadian abnormality, CBTi may target some sleep problems in SzSD more effectively than others, as this group experiences a mixture of both insomnia and circadian dysregulation. Chiu et al. (2018) show that the greater benefits of CBTi may be observed in those with classic severe insomnia, with short sleep duration and low sleep efficiency, than in those with poor sleep with normal or excessive sleep duration [8].

Researchers recommend sleep as an important treatment target in people with SzSD [3, 5, 34], but attention to sleep in secondary care mental health services is limited. Staff often lack the knowledge and confidence to intervene [35–37]. Adapted CBTi for people with schizophrenia and related disorders delivered by clinical psychologists has shown positive results, with significantly better sleep outcomes than treatment as usual [8, 33, 38–40]. However clinical psychology and psychological therapies are scarce and costly resources within secondary care mental health services [41, 42]. Furthermore, there are many other well evidenced therapies, such as CBT for psychosis [43], family intervention [41, 44], and cognitive behavioural treatments for people who self-harm [45], which compete for the time of psychologists and psychological therapists. It has been suggested by many authors that the skills of occupational therapists (OTs) align well with the treatment of sleep problems via behavioural, educational and environmental interventions [35, 46–48]. Occupational therapists focus already on activities, routines, and meaningful occupation [49–51], environmental adaptation [52], holistic assessments and consideration of complex systems [49, 53, 54], and work around personal motivations (volition) [55, 56]. Although there is a good argument for this type of intervention delivered by OTs, the optimal approach and its feasibility has not been empirically evaluated.

There is an argument for evaluating acceptability and efficacy of one of the adapted CBTi protocols referenced above, when delivered by OTs. We have focused here instead on developing a novel intervention for delivery predominantly by an individual OT for two reasons: 1. because a formal expert opinion process has not previously been applied to the development of this type of intervention, 2. to generate a therapy which makes best use of the pre-existing skills of OTs.

It is increasingly recognised as important to incorporate the views of those who could receive the intervention during the intervention design process [57]. This study sought to explore opinions of experts and people with personal experience, on the most appropriate content, format and delivery methods for an intervention to be delivered by mental health OTs, to improve sleep in SzSD. Through this process we aimed to co-design an acceptable and feasible intervention, tailored to the needs of people with SzSD, and to delivery by mental health OTs. We believe this to be the first study to explore and examine expert opinion to develop a treatment for poor sleep in SzSD, and the first to examine expert opinion to direct the treatment of sleep by OTs.

## Methods

The study received a favourable ethical opinion from South East Scotland Research Ethics Committee 02, 18/SS/0122. Written informed consent was obtained for in person participation, audio recorded consent for interviews, and implied consent for survey responses.

Our methods were based on the principles of Delphi methodology, including recruitment of relevant experts, iteration between rounds, and presentation of responses from earlier rounds back to participants [58–60]. We did not aim to reach or force consensus [61], placing equal value on identifying where views diverged.

The three online survey rounds with professionals, were followed by an in person modified nominal group technique [62] session with service users and carers with relevant personal experience.

### Sample

We recruited two separate samples, 55 clinical and academic topic-specialists (56 recruited), and 30 people with relevant personal experience (service users and carers); eighty-five participants in total (see Tables 1 and 2).

**Table 1:** Demographic data for professional participants.

Total professional participants		56
Clinical role type*^,^ **^:^	Senior specialist sleep OT	19
	Senior mental health OT	14
	Consultant psychiatrist	7
	Clinical psychologist	5
	Consultant (medical, other)	1
Academic role type*^,^ **^:^	Doctoral (final year)	6
	Post-doctoral	7
	Lecturer / professor	9
	Head of lab / department	3
Participant selected for expertise in**:	Sleep and circadian rhythm	39
	Mental health	32
	Occupational therapy	31
Country of residence & work:	UK	36
	Elsewhere in Europe	5
	USA	8
	Canada	3
	Australia	3
	Asia	1

* = at time of participation,

** = multiple may apply, OT = occupational therapist

**Table 2:** Demographic data for participants with personal experience.

Total participants with personal experience		26
Source of personal experience	Service user	20 (77%)
	Carer / significant other	6 (23%)
Age	Mean: 46.12 (SD = 15.68)	range: 19–80
Gender	Female	8 (31%)
	Male	17 (65%)
	Prefer not to say	1 (4%)
Ethnicity	White British	17 (65%)
	Other	6 (24%)
	Prefer not to say	3 (12%)
Diagnosis (service users only)	Schizophrenia	11 (42%)
	Schizoaffective disorder	2 (8%)
	Delusional disorder	1 (4%)
	Psychosis not otherwise specified	6 (23%)
Types of sleep problems experienced by you or the person you care for: problems with …	Getting to sleep	18 (69%)
	Staying asleep	16 (62%)
	Unrefreshing sleep	7 (27%)
	Sleep timing	9 (35%)
	Sleeping for too long	4 (15%)
	Difficulty waking	9 (35%)
	Nightmares	9 (35%)
	Sleep-disordered breathing / Obstructive Sleep Apnoea (OSA)	1 (4%)
	Restless Legs Syndrome (RLS)	4 (15%)
Advice previously received from… (could select 1 and 2)	Mental health care professionals	15 (58%)
	Other HCPs	7 (27%)
	Neither	5 (19%)
Intervention previously received…	CBTi (computerised or in person)*	1 (4%)
	Specific hypnotic	9 (35%)
	Other prescription sedatives (e.g., Antidepressant, antihistamine)	3 (12%)
	Continuous Positive Airway Pressure (CPAP)	0 (0%)

*Although one participant reported receiving CBTi this participant did not describe anything relating to this in written answers or during focus group discussion, it is possible this participant received another type of CBT.

**Fig 1:**
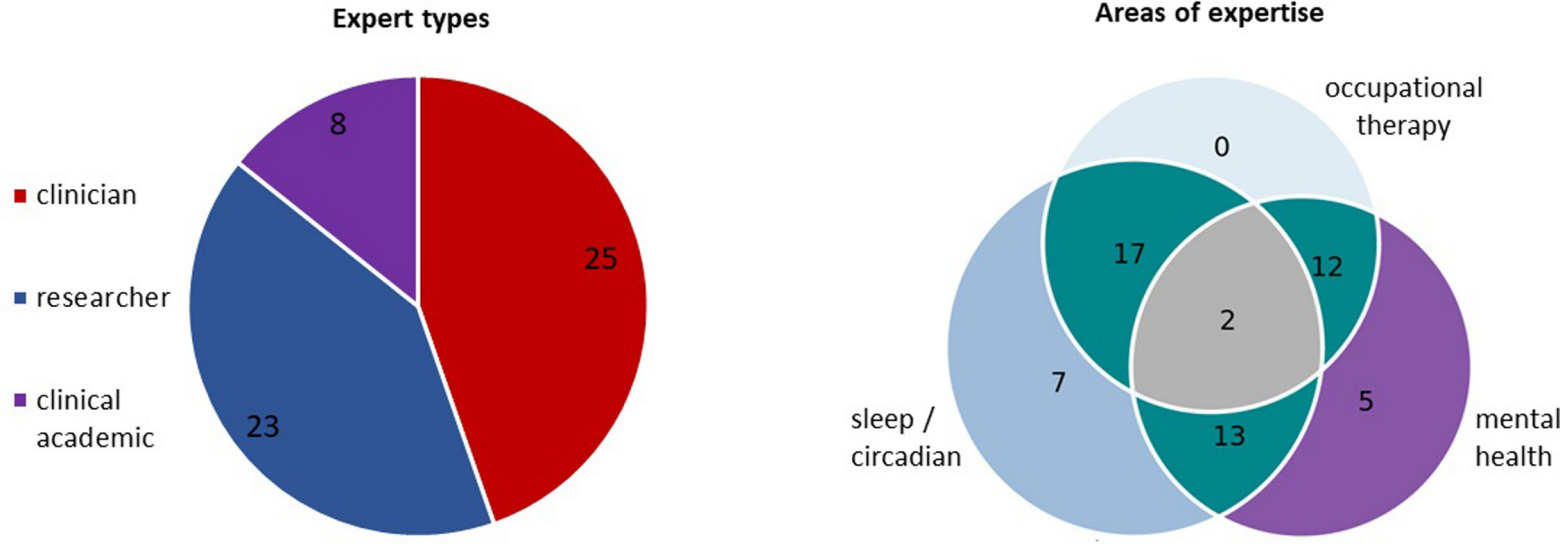
Types of expertise in professional participants.

We were aware that it would not be possible to recruit a large enough sample of occupational therapists with strong expertise in improving sleep in people with schizophrenia spectrum disorders, because this is as yet an underdeveloped area for the profession. As a result, we chose to recruit a range of expert groups each with one or more relevant angles to contribute (*occupational therapy*, *sleep and circadian rhythm*, and/or *mental health and schizophrenia*) (see also Fig 1 in Results). The large target sample accounted for heterogeneity of participants with diverse types of relevant experience [63].

Sampling was purposive, seeking representation of views and expertise from different groups through quota sampling [64], later stages of recruitment then specifically targeted any gaps [65]. Our expert group included clinicians and researchers specialising in insomnia, circadian rhythms, mental health, and occupational therapy (and in many cases multiple of these topics). We included clinicians and researchers, to ensure both performative and epistemological knowledge were considered [66].

We selected 15 as a target sample size for most professional sub-groups as many sources suggest a minimum sample of 12 in a homogenous group may produce adequate saturation [67, 68], and that 8–15 is adequate for Delphi studies conducted in homogenous professional groups [63].

We set minimum criteria for each category, and then began by compiling lists of those we thought were most qualified to comment, then sent invitations individually by email. People with personal experience were recruited via clinical services and through publicly displayed posters and leaflets. We had less control over purposive sampling of people with personal experience, although we did ask recruiters to focus on less represented groups as recruitment progressed (e.g. female service users).

Clinicians and researchers were sought with the following targets:

- Sleep experts (15)
- Occupational Therapists with expertise in sleep (10) (smaller target due to smaller total population)
- Mental health occupational therapy experts (15)
- Mental health experts and other senior mental health stakeholders (15)

Service user and carer participants were sought with the following relevant personal experience:

- Self-reported or referrer reported diagnosis of a schizophrenia spectrum disorder
  AND
- Experience of sleep problems (current or past)
  OR
- Carer of / close supportive contact of someone meeting the above criteria

### Data collection

Data collection instruments were designed in collaboration with patient contributors and were developed iteratively based on the results from previous rounds, as well as the wider evidence base and study aims. Rounds 1–3 were with clinicians and researchers, and a separate final stage sought views from participants with relevant personal experience.

Round 1 focused predominantly on eliciting participants’ pre-existing views on what elements should be included in the intervention and how. Round 2 focused on rating and ranking of possibilities and suggestions, and Round 3 included further rating and further qualitative exploration of remaining controversial questions. The final stage of a service users and carers modified nominal group technique again asked about optimal content and format, but with more focus on intervention acceptability. Later rounds included graphs, and para-phrased quotes from the previous rounds, please see S2–S4 Files.

#### Round 1: Clinicians and researchers. Online survey #1

After a brief introductory video, written information was delivered to meet the differing needs of each group (for instance background re: the role of the occupational therapist (OT) was provided to sleep experts, and background re: sleep and circadian rhythm was provided to mental health OT experts). Open questions were used, with free-text responses, beginning broad and soliciting participants’ suggestions, then only later providing more specific prompts to consider, and options to rate and comment on certain areas.

#### Round 2: Clinicians and researchers. Online survey #2

Where there appeared to be a strong consensus already in Round 1 this information was presented as ‘agreed’. Summaries of content from Round 1 were presented back to participants through qualitative paraphrased comments, and graphed rating results, with summarised rationales given for competing suggestions. Items were rated on a 5-point scale (very important, important, neutral, not important, better NOT to include). Participants were also asked about optimum order of delivery (early, middle, late, doesn’t matter when), and about which elements should be ‘core’ (given to all service users) or ‘optional’ (given when needed) or ‘do not use’ (categorical options) (an item being important to have available to use, was not the same as saying it should be used with all service users). Participants were able to add free-text comments justifying opinions or elaborating.

#### Round 3: Clinicians and researchers. Online survey #3

Views from previous rounds were presented, including differences in views between different participant groups. Remaining areas of controversy were further examined, and feedback was given where consensus had occurred, with the opportunity to comment further. Finally, participant views were sought on some implementation issues raised in earlier rounds.

Rounds 1–3 launched in October 2018, November 2018 and January 2019. We allowed around a month to obtain responses to each email survey round, keeping gaps between rounds to less than a month. We sent up to a maximum of 5 reminders for Round 2 and Round 3. We also offered a thank you gesture of a £20 voucher claimed at the end of Round 3 (final round).

#### Individual interviews: Optional, during rounds 1–3, with existing professional participants

All Round 1 participants were invited to arrange an interview if interested, 16 were able to be scheduled and conducted, those providing interviews were quite evenly spread across expert groups. Interviews explored the same overall research aims, except focusing more on whichever dimension of the intervention that participant had experience and views on. Participants were asked to elaborate on or clarify their answers to earlier rounds where relevant, and sometimes were presented with competing viewpoints to their own to respond to (without making other participants identifiable). This served to provide additional qualitative depth and explanation of rationales for proposed approaches.

#### Final stage: Modified nominal group technique: Service users and carers with relevant personal experience

We presented results from rounds 1–3 to participants for discussion and voting. We used graphs, images and verbal explanations. We presented both areas on which a provisional consensus has been reached, and areas where multiple options and approaches were still under consideration. There was a focus on acceptability of potential aspects of the intervention, format and presentation, expectations of effectiveness, and barriers and facilitators to engagement. There was anonymous voting (on paper) regarding various intervention components presented, focus groups discussions (in five smaller groups), presentation back of points and views from these groups, and then further anonymous voting. The focus groups were between 4–6 participants each, and were chaired in tandem by the first author, and the co-facilitators named in the acknowledgements (some facilitators chaired in pairs). All are mental health researchers or clinical academics. For the topic guide see S5 File. We opted to group carers together on investigator and public patient involvement (PPI) suggestion.

This took place in person and participants were reimbursed £50 for their time and provided travel expenses and lunch.

### Analysis

The progression from the qualitative exploratory aspects of round one, to rating of resulting ideas from round 2 onwards can be characterised as a form of mixed methods exploratory sequential analysis [69]. Analysis of free-text data was using qualitative content analysis [70], including counting [71]. Descriptive statistics and graphs were used to present and analyse Likert scale ratings.

Free-text data was managed in Nvivo (version?), each ‘case’ was coded by participant attributes and participant group and sub-group (e.g., sleep, mental health, OT). Data was coded using both inductive and deductive codes [72]. Deductive coding was using a predetermined framework of codes relating to topics asked about and those expected based on literature (e.g., “Bed or sleeping surface”). Inductive codes were arrived at in response to the data, prompted by the data (not predetermined).

In some cases, statements were classifiable into specific sentiments or suggestions, and content within that topic was coded for each ‘statement’ (e.g., “Advocating Sleep Restriction Therapy (SRT)”, “Be cautious with SRT”, “SRT could trigger mania / psychosis”, “Do not use SRT”, “Use sleep compression instead of SRT”). This allowed use of counting to corroborate or contradict the impression of relative popularity and importance of each theme or sentiment, and prompt further analysis [70]. Data was searched for counterexamples before ‘many’, ‘most’ or ‘all’ statements were made. Caution was applied in over-interpretation of ‘counts’, as they do not show the strength and clarity of expression of a given statement, and they are not equivalent to ratings on the standard items. Similar codes were merged or linked, and related codes were grouped hierarchically to form themes. Contradictory or incompatible ideas were identified as candidates for further analysis, or for questions in later rounds.

Our two, lead PPI contributors who wished to be involved in analysis were shown anonymised data excerpts, the codes and themes being developed, and graphs of the Likert scale responses, and contributed toward decisions on questions for subsequent rounds, and descriptions of the overall findings.

### Assessing consensus

Consensus was assessed via a combination of similarity of qualitative content between participants from different groups, and relative levels of agreement in Likert ratings of items for appropriateness and importance to include. In determining what items to include, we considered not just likely effectiveness, but also affordability, practicability, and cost-effectiveness (APEASE criteria for designing and evaluating interventions or intervention ideas) [73]. The ‘cost’ of delivering an item, in this case, includes for example time to train therapists to deliver, time to deliver, and time to assess with each participant whether it is relevant to deliver. As part of practicability, we considered compatibility of potential components for delivery together.

We had originally planned to assess extent of consensus by calculating median and interquartile range of Likert responses [74], seeking an interquartile range of 2 points or less. In practice this proved unhelpful as by this criterion there was a high level of consensus to include almost all items rated, whilst at the same time, many participant views suggested keeping the intervention as simple as possible (see sub-theme ‘keep it simple’). Seventy-five percent is often considered an acceptable level of consensus [61]. Few items included with under 75% endorsing either ‘appropriate’ or ‘very appropriate’ and many had much more (e.g., 90–100%). Where lower percentages of professional participants endorsed an item, we focused on examining differences in views between participant groups (e.g., CBTi therapists VS mental health clinicians), and what qualitative rationales were given. Although re-rating was completed for some Round 2 items in Round 3, on other occasions we instead opted to request ratings of more granular items, or new suggestions not rated in the prior round (see S1 and S4 Files).

Whilst we were able to examine consensus via multiple-choice ratings for larger domains (e.g., addressing light exposure), it was unrealistic to ask participants to rate all potential intervention protocol sub-components at the most granular level (e.g., if sleep is delayed encourage a morning walk). Some key suggestions on specifics were brought to later rounds for rating where we judged more views were needed; others were assessed based on congruence and compatibility of the free-text responses with each other and with relevant literature.

## Results

### Participants

We were able to exceed all our sub-group targets for all clinical and academic professional groups except mental health occupational therapists (target = 15, recruited = 14). Many participants fit into more than one subgroup, and we achieved a balance of clinical and academic expertise, as shown in Fig 1.

Uptake from initial email invitation for Round 1 was good (66% of those invited participated). We overshot our target by one participant, as it was not possible technically to lock the survey to prevent further participation without also preventing part-completed responses from being finished. Retention through Round 2 (98% partially, 94% total completion) and Round 3 (96% partial, 95% total completion) was good. We allowed participation in Round 3 if Round 2 was missed. Although we anticipated relatively short free-text responses in the survey rounds, many participants entered rich multiple-sentence responses. Sixteen participants provided individual interviews between rounds 1 and 3.

As our Modified Nominal Group Technique with participants with personal experience was completed on a single day it was not possible to recruit further to replace participants who had to cancel at short notice or who did not arrive on the day. The participants were relatively diverse, although the low proportion of female participants should be noted, which appears to be at least somewhat in excess of any differences in gender balance of schizophrenia [75]. Problems getting to sleep and staying asleep were more common in our sample (69%, 62%) than irregular, reversed, unrefreshing or excessive sleep (27%, 35%, 15% respectively) (see Table 2).

## Results

The full findings are presented in S1 File, for qualitative data excerpts and graphed results of all multiple choice and Likert responses the below summary may be read alongside this supplement. Findings are summarised here in Table 3, as themes (1–7) and sub-themes. Themes 1–3 concern the content of the intervention (see also Table 4): 1. Intervention targets and scope (what problems to treat and in whom), 2. The assessment, and 3. Intervention domains (what broad areas to address, using what specific components). Themes 4–7 concern the manner of intervention delivery: 4. Personalisation, 5. Format, Structure and Pragmatic considerations, 6. Therapeutic Approach and therapist factors, 7. Implementation considerations.

**Table 3:** Summary of content themes and sub-themes within data.

Broad topic area	Sub-topic / sub-theme	Specific suggestion or issue raised
INTERVENTION TARGETS AND SCOPE	Sleep problems and sleep interferers	Sleep effort and frustration
		Worry, rumination, stress and anxiety
		Psychotic symptoms
		Fear of the dark
		Fear of silence
		Fear of the bed
		Fear of sleep
		Long sleep
		Difficulty rising & sleep inertia
		Physical illness / physical symptoms
	How far to address ‘other’ sleep disorders	Screen for sleep-disordered breathing (SDB) and parasomnias
		Nightmares
		Assess nightmares
		Directly address nightmares specifically
		Nightmares may improve through treating sleep
		Refer on regarding nightmares
	Stability to intervene	How well or stable would clients need to be to benefit?
		Stability of social situation important
		Stability of medication important
		Concerns about exclusions
	Transdiagnostic intervention? (applied across diagnostic groups)	The intervention should be applied trans-diagnostically
		The intervention should focus exclusively on people with a schizophrenia spectrum diagnosis within this study as they are harder to reach
THE ASSESSMENT	Format & manner of assessment	Use an interview
		Use checklists and / or standardised questionnaires
		Rapport in assessment
	Prioritisation of areas to assess	-
	Longitudinal self-report of sleep & activity (activity & sleep diary)	Sleep diary
		Activity diary
		Diary burden & difficulties
		Completing diaries as an intervention
		Format options, prompts and support
		Possibility of using an app
	Passive monitoring within the assessment	Self-report and passive monitoring results will differ (useful to compare / need both)
		Passive monitoring as an intervention
	Measurement of light exposure	Measurement or self-reporting of light exposure at baseline and during the intervention
INTERVENTION DOMAINS	Sleep schedule	Address sleep schedule regularity
		Regular rise time
		Regular bedtime
		Allowable flexibility in sleep schedule
		Need to fit sleep in with life
		It might be OK to be nocturnal
		Gradual approach to sleep timing changes
		Stabilise timing first before changing times
		Support to change sleep times
	Time in Bed restriction	Advocating Sleep Restriction Therapy (SRT)
		Be cautious with SRT
		SRT could trigger mania / psychosis
		Do not use SRT
		Use sleep compression instead of SRT
		Not keen to try reducing time in bed
		Already reduce time in bed, & advocate it
	Napping	Allow napping
		Avoid napping
		Evaluate naps
		Nap duration
		Nap timing
		Replace naps with activities
		Schedule naps
	Stimulus control, and managing awakenings	Avoid non-sleep activities in bed / bedroom
		Use ‘the 15 minute rule’ or similar
		Bad experience using ‘the 15 minute rule’ as self-help advice
		Address activities to do if awakening in the night
		Provide education on awakenings being normal
	Morning routine	Address type of activities
		Use of alarms
		Dawn simulator alarms
		Education on sleep inertia
		Experience of struggle with waking
	Evening routine	Evening wind-down activities, lower stimulus
		Preparation for bed before wind-down
		Prepare for the next day—if relevant
		Support to find suitable activities
		Get ready for bed alarm
	Daytime activity	Increasing amount of activity
		Address activity type
		Address activity timing
		Scheduling activities
		Routines and habit formation
		Meaning, satisfaction and enjoyment
		Support to find and plan activities
	Addressing medications	Consider side effects
		Addressing timing of prescribed medications
3. INTERVENTION DOMAINS (continued)	Addressing food and drink	Consider food and drink timing
		Address avoiding late eating
		Address skills and / or routines around meals
		Night eating
		Consider food and drink content
	Addressing substance use	Substance use
		Alcohol
		Caffeine
		Nicotine
	Light Exposure	Modifying light exposure
		Timing of modifications to light
		Morning light exposure
		Daytime light exposure
		Increasing evening light
		Reducing evening light exposure
		Reducing light at night
		Method to modify light
		Light box
		Light visor
		Blue-blockers
		Modifying light in the home & bedroom
		Using outdoor light / natural light
		Season is important
		Embedding light in activity / occupation
		Education regarding light, circadian rhythm and mood
		Low expectation of efficacy regarding light
		Acute alerting effects of light
	Environmental assessment and intervention	Home environment
		Bed or sleeping surface
		Bedroom / bed not for non-sleep activities
		Having other useable rooms
		Noise in the bedroom
		Temperature in the bedroom
		Air quality
		Sensory factors
		Pets in the bedroom
		Home environment intervention
		Feeling safe in the home
		Social environment & context
		Social environment in the home
		Support from friends, family and carers
		Social commitments
		Peer support
		Loneliness
		Cultural factors
	Relaxation and / or mindfulness	Relaxation technique
		Breathing techniques
		Mindfulness meditation
3. INTERVENTION DOMAINS (continued)	Thermoregulation	-
	Addressing sensory factors	-
	Cognitive or psychological approaches	Cognitive or psychological approaches
		Psychological approaches better dealt with by psychological therapist
4. PERSONALISATION	-	The goals of the intervention should be individually determined
		The methods of intervention should be personalised
		Limits to personalisation
FORMAT, STRUCTURE AND PRAGMATIC CONSIDERATIONS	Personalisation and complexity vs simplicity to deliver	Personalisation
		Keep it simple
	Format of intervention and assessment materials	Format options & literacy
		Use of technology in delivery of the intervention
	Core vs optional components	-
	Order of delivery	-
	Follow up and ending of therapy	Maintenance plan
		Follow up / tapering of ending
THERAPEUTIC APPROACH AND THERAPIST FACTORS	Therapeutic approach, therapist attitude & manner	An educational approach
		Education re: normal sleep
		Normalising
		Experimentation
		Benefits of change, motivational interviewing approach
		Therapeutic rapport & listening
		Rapport required before home assessment
	Therapist knowledge, skills & confidence	Therapist confidence in delivering the intervention
		Relationship to OT role & skills
		Generic working barrier to OT interventions
IMPLEMENTATION CONSIDERATIONS	Reaching referrals	-
	MDT approach	MDT knowledge & attitude
		MDT approach to intervention
		MDT approach to medication
		MDT approach to maintenance

**Table 4:** Summary of findings regarding intervention domains to address and how.

Domain	Consensus to include*	Congruence and compatibility of suggestions on *how* to address (if included)	
	Strength	Rating	Brief description
Sleep schedule	Very strong	Mostly congruent	Some disagreement re: level of rigidity of regular rise time required.
Time in Bed restriction	Weak—conflicted	Somewhat congruent	Strong feelings for and against. Some variability in level / manner of restriction.
Addressing napping	Strong	Conflicted	Consensus to evaluate napping, conflict regarding extent to reduce / allow naps.
Stimulus control, and managing awakenings	Strong	Congruent / conflicted	Consensus re: non-sleep activities away from bed, views differ re: 15min rule.
Morning Routine	Strong	Mostly congruent	General agreement re: creating morning routine, some variation re: alarms
Evening Routine	Very strong	Congruent	Strong agreement re: setting similar calming evening routine.
Daytime activity	Very strong	Very congruent	Compatible suggestions from all participant groups.
Addressing medications	Moderate	Somewhat congruent	Views vary on far to address and with how much prescriber input
Addressing food and drink	Moderate	Congruent	Consensus re: late eating, less re: food timing, least consensus re: food content
Addressing substance misuse	Moderate	Congruent	Disagreement only re: how personalised or flexible to be re: caffeine reduction
Light exposure	Strong	Somewhat congruent	Agreement to address, some variance on priority level, and means to modify light
Environmental intervention	Strong	Very congruent	Similar suggestions on all aspects except re: reducing / blocking noise at night.
Relaxation and / or mindfulness**	Weak—less priority	Somewhat conflicted	Raised often (together), not often highly prioritised, incongruent re: best approach
Thermoregulation	Weak—less priority	Congruent / conflicted	Agreement re: bedroom temp & bedding, disagreement re: socks & baths
Addressing sensory factors	Weak—less priority	Congruent	Argument against inclusion to prioritise other areas, but brief to address.
Cognitive or psychological approaches	Weak—less priority & conflicted	Somewhat congruent	A little conflict re: whether in scope of OT, also less prioritised than other areas.

*There were no domains with a consensus *not* to address.

**We acknowledge these are not equivalent, but they were usually discussed together and form one domain in these findings.

### 1. Intervention targets and scope

From the outset participants were told the intention was to develop an intervention to improve poor sleep, including insomnia and circadian dysregulation. All participants appeared to assume that sleep onset, maintenance and timing were valid targets. People with personal experience rated ‘feeling more alert’, ‘sleeping at night, not in the day’, ‘waking less often’, and ‘falling asleep without getting stressed’ as the most important targets.

#### Sleep related distress

Participants identified problems to be targeted: frustration when trying to sleep (intense sleep effort), non-sleep related worry preventing sleep, the impact of psychotic symptoms, sleep related fears, and the social and functional impact of long sleep and difficulty rising.

#### How far to address ‘other’ sleep disorders

Some participants suggested screening for sleep-disordered breathing and common parasomnias, and referring to sleep services. Others did not mention these conditions.

All who commented on it, agreed to assess nightmares. There was disagreement about how far the intervention should address nightmares; professionals often noted specialist psychological therapies are contingent on therapist training. It was noted that some non-specific approaches, e.g., stabilising and consolidating the sleep period, might improve nightmares, without requiring psychological therapy skills training.

#### Stability

There was some consensus that a behavioural sleep treatment was unlikely to be effective either during acute psychiatric crisis, or during environmental instability, such as homelessness, and ratings agreed (66% in round 2 rising to 71% in round 3). Some participants suggested that addressing sleep problems in acutely unwell patients, for example hospital inpatients, needed a different intervention.

#### Transdiagnostic intervention?

Although it had not been our aim to explore this, many participants suggested the intervention could be suitable irrespective of diagnosis, or said the study should not be diagnostically focused. Once we asked in round 3, 62% endorsed this in some form. A large minority however suggested retaining the diagnostic focus because those with SzSD may be harder to reach and less likely to be offered non-drug interventions.

### 2. The initial assessment

#### Format and manner of assessment

Professional participants emphasised establishing rapport, whilst also finding out a potentially large amount of information on many areas. An interview format was recommended, incorporating structured elements. Some people with personal experience felt a home assessment requires developing trust and so should be done later, and for some a home assessment was not acceptable.

#### Prioritisation of areas to assess

Very many assessment areas were suggested by professionals, and were highly endorsed in ratings, however it was acknowledged that covering everything in full depth would be laborious. We asked about areas to prioritise, some were acceptable to explore in-depth only if necessary.

#### Longitudinal self-reported assessment of sleep and activity

Although both professionals and people with personal experience described completing a sleep and daytime activity diary as burdensome, they also felt the data this would produce would be very valuable to guide intervention. Many professionals felt the process of completing this might have some direct beneficial effect. Participants recommended seeking an easy-to-use format, possibly offering format options, and endorsed reminders to complete (65% = ‘important’ or ‘very important’, personal experience).

#### Passive monitoring within the assessment

Professionals varied in their prioritisation of subjective or objective measures, but participants agreed that both combined provided valuable insight. The process of comparison was noted as potentially useful in itself, by both professionals and those with personal experience. Participants with personal experience were mostly willing to wear an activity monitoring watch (42% = definitely, 25% = probably, 12% = possibly, 21% = not really), and suggested a longer period of wear for assessment before beginning the intervention than did professionals on average (3 or 4 weeks versus 2 weeks).

Some suggested varying the length of the baseline passive monitoring period depending on the type of sleep problem being assessed (circadian rhythm problems potentially taking longer to capture than classic insomnia), others felt this complicated matters.

### 3. Intervention domains

Consensus, controversy and dilemmas around which domains to address are discussed, see Table 4 for a summary.

#### Sleep schedule

Suggestions were largely consistent to increase regularity of sleep timing, with some variation around the level and timing of any allowable flexibility. There was more support for occasional flexibility to accommodate life events once a routine is established, than for flexibility to recover sleep debt, although a minority did advocate the latter.

Although two participants with personal experience suggested it might be OK to be nocturnal / reversed, they described this as merely better than getting no sleep, rather than a preference, and the goal of ‘sleeping at night, not in the day’ was one of the most highly rated by this group. Suggestions were consistent regarding changes to sleep timing being made in small increments, and two professional participants suggested first stabilising timing, then moving the sleep window, if practical.

#### Time in bed restriction

Time in bed restriction was controversial, and views diverged further as rounds progressed. Qualitatively, strong views were expressed on both sides. Participants emphasised the efficacy and evidence base for sleep restriction therapy, and others emphasised the potential risk of adverse effects such as triggering mania or psychosis. Some felt this meant it should not be used, whilst others suggested it should be used with caution, regular monitoring, and adequate therapists training and supervision. Sleep compression was suggested as a gentler alternative approach (reducing the sleep window gradually rather than in one step), and this was then endorsed by 56% (28% neutral, 6% disagree, 0% strongly disagree).

Participants with personal experience expressed some reluctance toward the idea of time in bed restriction and avoidance of napping, which may have been driven by their lack of belief that this approach would work (ratings of feeling it would be likely to work: 35% = not really, 44% = somewhat, 20% = mostly, 4% = completely). This was quite in contrast to professionals who felt it was likely to work, but difficult to adhere to or might have adverse effects. Participants with personal experience rated this as the area of which they had second-lowest prior awareness, after light exposure.

#### Napping

Professionals endorsed addressing napping, and agreed strongly to evaluate the role of naps, but were divided between ‘avoid napping’, ‘allow napping’ and ‘encourage a regular planned nap’. Some said it depended on the individual’s mental health and co-morbidities, whilst others felt avoiding or reducing napping was always preferable. Keeping naps short and not too late in the day (if taken) was suggested and was uncontroversial, as was a safety nap if driving and sleepy (or, do not drive). In participants with personal experience, some thought avoiding napping might be useful whilst, some already did not nap, and some would not be willing to try this. Qualitatively some expressed they felt one should ‘get sleep whenever you can’.

#### Stimulus control, and managing awakenings

Professionals and people with personal experience alike agreed on making the bedroom, or at least the bed, for sleep and sex only (if other rooms are available). Rising if not asleep was mostly uncontroversial with professionals, who varied more on after how long to get up, or whether this depended on how you were feeling (e.g., sleepy, or irritable and alert). Much discussion explored how best to support clients to find suitable activities to engage in during the night if they wake.

By contrast, many people with personal experience had tried ‘the 15 minute rule’ or similar, as part of self-help advice, and in 4 out of 5 of the focus group discussions participants independently raised problems with this approach. People described getting less sleep, feeling worse in the daytime, waking up even more, and becoming engrossed in activities so not going back to bed. It was not clear how long participants had persevered, although we know these were not experiences as part of structured CBTi as participants had not had CBTi (see Table 2). No-one replied to describe any good experiences when attempting to use the 15-minute rule.

#### Morning routine

Morning routine was rated as very appropriate to address in round 1 (>80%), and suggestions were consistent regarding how to address this. These focused around energising activity, and some emphasised light exposure soon after waking, planning activities to improve motivation to get up, and use of alarms. ‘Support to set alarms’, and ‘dawn simulation alarms’ were rated highly by professionals. Dawn simulator alarms were also popular when discussed by participants with personal experience, none had tried them, but many felt they might help.

The use of multiple alarms set away from the bed was rated highly by professionals. Participants with personal experience emphasised the real struggle to wake, and many emphasised that harsh approaches were probably necessary and acceptable if they can be woken. Some already asked friends to phone to wake them up or arranged activities they would have to get up for. We note the daytime nature of the discussion group may have meant those with ongoing delayed or reversed sleep were underrepresented (discussed in limitations).

#### Evening routine

Evening routine was discussed very frequently, and almost all the specific suggestions rated, were rated as very important to include. Participants with and without personal experience suggested similar types of activities for therapists to suggest or encourage within the evening routine. The exception being watching TV, which some strongly advised against, whilst others felt it was not a major problem. There was varied emphasis on having a *similar routine each night* during the wind-down period in the qualitative content, but this was rated highly by professionals when asked. Many participants with mental health expertise suggested it might be necessary to support participants to identify and plan suitable activities, describing using lists of ideas, or providing materials. Two professionals and one person with personal experience recommended the option of setting an alarm to cue initiating the getting ready for bed routine.

#### Daytime activity

Daytime activity was rated in round 1 as one of the more important areas to address, perhaps influenced by knowledge that it will be OTs delivering the intervention. The types of interventions to address daytime activity were very consistent between participants, and involved promoting occupational balance in routines, identifying interests, scheduling activities, and setting goals (resembling common mental health OT approaches). Participants with personal experience concurred regarding the barriers, and the approach needed: describing too few satisfying productive activities, the need to increase activity and exercise, and to make very concrete and specific plans to increase follow-through. This specific scheduling fits well with the suggestion ‘continue with activities planned irrespective of sleep’ (akin to behavioural activation), which had been well endorsed in round 2 ratings by professionals.

#### Addressing medications

Some participants suggested addressing the exact timings of oral medications, particularly those which are sedating and taken at night, levels of confidence to discuss medication timing varied (even within the ‘nocte’ prescriber instruction). Many suggested liaising with the prescriber or a pharmacist. Mental health clinicians and researchers were more likely to think this was important to address. Participants with personal experience were more focused on addressing or considering side effects of medications. Sometimes professionals mentioned addressing medication dosages in liaison with the prescriber.

#### Addressing food and drink

Meal timing was rated as important, but less so than other areas, and type of food and drink was only rated as important by a few participants. Some linked daytime meal timing to circadian rhythm and daytime routine, others only focused on late eating causing sleep interference. To avoid late night eating, some participants (mostly but not exclusively OTs) noted it might be necessary to address food preparation and shopping skills and routines elsewhere in the day. The same was said about night eating, although more tentatively. Participants varied a lot in how common they thought night eating might be in this group and this did not seem to relate to amount of experience with people with SzSD. One person admitted not asking about night eating as much as they thought they should. Religious and cultural considerations were noted (e.g., Ramadan).

#### Addressing substance misuse

Participants in round 1 commonly and consistently suggested addressing illicit substance misuse as far as possible and as far as the client is willing. They also noted though some levels of substance abuse that would preclude useful participation in the intervention such as regular amphetamine use.

Education regarding the impact of alcohol on sleep was emphasised. Late caffeine or high levels of caffeine was recommended to address, however participants varied in how strict or how personalised rules should be. The impact of smoking was acknowledged; some professionals said ‘reduce smoking’ or ‘avoid late at night’, but professionals and people with personal experience noted reducing smoking was hard to do.

#### Light exposure

Increasing morning or daytime light exposure was regularly emphasised in qualitative data, more so than reducing evening light. Professionals, including circadian rhythm researchers, varied in how strong they felt the evidence was for reducing evening light exposure. Whilst no professionals described altering light exposure as harmful, a minority felt it was relatively unimportant or not worth the effort / burden. Professionals spoke of circadian effects, a small number spoke specifically about the acute alerting effects of light (4 professionals, one person with personal experience). Some said the importance of light will vary between individuals. Views among those with personal experience varied regarding light.

Using natural light was by far the most endorsed method to modify light exposure, partly for accompanying benefits of social contact and physical activity. A few participants, mostly those with mental health expertise, spoke about embedding light exposure within activity / occupation. People with personal experience were reasonably willing to go outside for natural light, more than to use a light box, although many also expressed difficulties and concerns around going out. Some described needing a lot of support to work toward going out, as more of a long-term goal. Modifying light exposure to improve sleep was rated as difficult to stick to (65% rated ‘somewhat difficult’, ‘difficult’ or ‘very difficult’).

Season was noted as influential, with both professionals and people with personal experience describing potential for sleep patterns depending on season, and some noting light boxes were more needed in winter than summer, as well as weather affects going out.

Reducing light at night during the sleep period was very often mentioned and advocated by professionals. Among people with personal experience, some felt darkness improved sleep, others experienced fear of the dark. Participants mentioned blackout curtains, eye-masks, and avoiding screens and bright lights during awakenings (but light enough to prevent falls).

Giving education regarding the effects of light was often discussed as important by professionals. In-keeping with this; people with personal experience demonstrated often quite poor understanding of the effects of light especially on circadian rhythm (understandably), and many had low expectations of efficacy from changing light exposure.

#### Environmental assessment and intervention

There was a strong consensus to address bedroom environment, and to a slightly lesser extent home environment generally. The specific suggestions which we requested rating of, were highly endorsed, especially addressing window coverings, and moving belongings to support stimulus control (bed only for sleep). There were many areas identified as potentially helpful to address but where financial constraints might limit options, such as poor-quality bed or bedding, or air quality related to mould and damp. Noise disruption at night was similarly ‘worth being aware of’; but might be beyond control, except by using white noise generators or earplugs—views on these were mixed. Improving the ability to return to sleep after disruptions, or reducing sleep related anxiety, could help with noise. Sense of security in the home would affect sleep, and might be modifiable via practical measures or discussions, or might be hard to change, depending on the basis of these fears. It was acknowledged that clients might not sleep in the bed for a range of reasons including trauma.

The social environment, both in the home and more broadly was noted as potentially a barrier or facilitator of successful sleep improvement. Either busy homes, or loneliness in those who live alone, could cause problems. Social commitments could facilitate regularity, or it might be unconducive to regular sleep schedules (e.g., work and family responsibilities might conflict with desired sleep pattern). Cultural factors must also be taken account of, for example religious observances, and varied cultural norms relating to sleep. Professionals and people with personal experience cited support from family, friends or carers as a facilitator should be utilised when present, although some lack informal support. Some professionals suggested using peer support (e.g., groups) within the intervention, while this was not mentioned by those with personal experience.

#### Relaxation and / or mindfulness

In round 1 many participants suggested inclusion of either mindfulness or relaxation. Ratings of breathing techniques, progressive muscle relaxation, guided imagery, and mindfulness meditation, endorsement and consensus were higher in round 2 then reduced by round 3. Some commented that relaxation or mindfulness were not enough of a priority to include in limited time, others felt it was a priority, and some strongly felt relaxation was *counterproductive* for sleep, particularly if practiced in bed with the intention of inducing sleep (induces sleep effort).

Although many treated mindfulness and relaxation as interchangeable or at least closely related in the context of a sleep intervention, some noted that mindfulness is not intended to produce relaxation.

Of those who advocated use of relaxation, there were suggestions to practice this in the daytime (although some did suggest practicing in bed), and to select a physically based strategy over for instance visualisation. Many suggested individual responses vary, and some thus suggested offering different options for clients to try, but then some pointed out the high cost in training, skills and time (of client and therapist) to try out multiple options.

#### Thermoregulation

Thermoregulation could be addressed in terms of the temperature of the bedroom, bedding and nightclothes, warming the extremities prior to bed (slippers, bed socks, hot water bottles), and pre-bed baths or showers. There was agreement regarding addressing appropriate bedroom temperature (cool-ish) and that optimum temperature varies individually.

Beyond bedroom temperature there was little agreement, readings of the evidence varied between professionals, as did practice-based experiences. Some felt warming the feet or bathing prior to bed were neither harmful nor effective, others felt pre-bed bathing / showering might add to a calming evening routine, others felt direct thermoregulatory effects were important in aiding sleep onset (drop in body temperature and vasodilation of extremities). Participants mostly agreed that these strategies did not take much time to address for therapist or client and were low risk; may help some, and could be tried at low ‘cost’. No one with personal experience expressed any strong views relating to temperature, some felt these interventions can be, or might be, helpful.

#### Addressing sensory factors

Although sensory factors were mentioned in some way by 52% of professionals, they were not given a lot of emphasis by many and were rated as relatively less important. They related mostly to the bedroom environment (colour, clutter), but also nightclothes. Many noted that sensory preferences are individual and can be identified by the client. More endorsed this as ‘optional’ than ‘core’.

#### Cognitive or psychological approaches

Addressing sleep interfering beliefs was rated as relatively important in round 1, some suggested this should be using cognitive or psychological approaches, although some suggested these might be better delivered by a psychological therapist. Some stated sleep interfering beliefs or cognitive processes would often be modified by behavioural approaches, and cognitive techniques not required. It was not possible to fully explore all the many therapeutic modalities and techniques mentioned. Overall views suggested some patients may benefit from use of some cognitive approaches if it is feasible to equip the therapist with relevant skills to use when needed, or if therapists already have some skills.

### 4. Personalisation

Professionals suggested personalisation of the *goals of the intervention* to suit clients’ priorities, to varying degrees. Similarly, people with personal experience rated very highly ‘find the best sleep times to fit your schedule’. Personalisation of the *methods of intervention* for different sleep phenotypes, or patient choice and preferences, was also suggested by both professionals and people with personal experience (personalisation of various components is discussed in their respective sections). Limits of personalisation were acknowledged, in the form of increased complexity and skill demand upon the therapist.

### 5. Format, structure and pragmatic considerations

A tension was identified by some participants between the desire to personalise, and the need to keep the protocol and the process simple, for both the client and the therapist. Increased personalisation options (content and delivery order) were noted to potentially increase the training requirements, whilst simplicity could improve therapist confidence, which was also important (discussed below).

Having options in the format of materials and around use of technology vs paper was described as straightforward, and was advocated. People with personal experience were positive about the use of technology, apart from those who were not confident using devices.

Most suggested some components would be optional, whilst a few recommended to cover everything. There was some consensus regarding some components which were ‘core’ (vs ‘optional’), especially: evening routine, psychoeducation regarding normal sleep, activity and occupation, and morning routine (85%-98% = ‘core’). There was weaker consensus regarding: modifying light exposure, sleep schedule and home environment being core components (all 70% = ‘core’). For other components views varied more. Rationales given for some items being optional were around personalisation to phenotypes, to preferences, or where sleep might be improved by an earlier ‘core’ component, making other items unnecessary.

Views were divided regarding pre-determined versus variable order of component delivery. Where it came to which domains should be addressed first, overall, more items were suggested for delivery early or middle, whilst less were suggested to be delivered later or ‘it doesn’t matter when’. Evening routine and psychoeducation regarding normal sleep were recommended to be delivered early (90%, 88%), and to a slightly lesser extent morning routine (73%), whilst views were more divided for other components. Often those which were rated as ‘optional’ elements were suggested to deliver middle, later, or doesn’t matter when (food and drink, thermoregulation, sensory factors, cognitive approaches to worry), with those rated as ‘core’ also most rated as ‘use early’. Exceptions included addressing medication (optional, use early), and activity and occupation (core, use middle).

There were suggestions around how to maintain gains: many suggested a follow up or booster session, or a tapered ending to therapy. Participants suggested producing a maintenance plan for the client and their care team to keep and refer to (choice of format to suit the client).

### 6. Therapeutic approach and therapist factors

An educational approach was promoted, and education on normal sleep, sleep pressure, and circadian rhythm was suggested often. This was very compatible with suggestions of a ‘normalising’ approach. Some spoke about promoting learning using experimentation / behavioural experiments, using motivational interviewing techniques to increase readiness for change, and the need to establish rapport. People with personal experience touched on rapport specifically regarding the home assessment, which some felt could otherwise be invasive—some participants feared judgement.

As well as an empowering approach, and establishing rapport, some professionals described the importance of the therapist being confident in their delivery, thus inspiring confidence in the client. Areas of the intervention where common OT skills would be well utilised were mentioned, including problem solving, teaching, listening, and exploring routines and activities with clients. Barriers to the delivery of this type of intervention by OTs were discussed, including lack of talking therapy skills / the need for training in this area. Three UK OTs independently raised the issue of generic working in community mental health teams, with some UK OTs becoming unable to deliver OT interventions as their roles had become focused on case management (care coordination).

### 7. Implementation considerations

Team perceptions were identified as a potential implementation barrier, as staff may not see sleep as the role of OT. Limited staff awareness of sleep might prevent identification of sleep problems, or staff might not be in the habit of asking clients about sleep as they feel they have nothing to offer. A potential facilitator of reaching the clients, was that clients were very willing to discuss sleep, and wanted support. Also, some who were not interested in talking therapy might be interested in a more behaviourally based and educational approach (comments from professionals and people with personal experience). Similarly, people with personal experience rated intervention ‘using elements of CBTi’ as relatively unappealing, despite that few (possibly none) had had CBTi.

A multi-disciplinary team (MDT) approach to the intervention was advocated, particularly around medication advice, and maintenance of gains after therapy is complete. Some described benefits of involvement of multiple professions, each with sleep specialism, directly in intervention delivery, whilst others framed MDT collaboration more as the individual sleep OT liaising and delivering information or training to those non-sleep specialist MDT members involved in the client’s ongoing care.

## Discussion

This paper has evaluated the views and recommendations of relevant experts regarding the appropriate contents and format for a mental health OT intervention to improve poor sleep, in people with SzSD. Although a clear consensus was not reached on several issues, the results were informative and provided a basis for the development of an intervention. Due to the diverse experiential and theoretical participant perspectives drawn upon, we anticipated that consensus may not be reached on some items. The results instead describe the arguments for certain approaches, and how views and approaches vary within and between groups. We hope these results may also prove informative for others making decisions about treatment of poor sleep in SzSD, and sleep treatment by mental health occupational therapists.

The findings emphasised the importance of personalisation within an OT-delivered intervention for poor sleep in SzSD, although the optimal manner and extent of personalisation described varied. Professionals agreed on what to assess, and agreed on a few domains to address with all clients. There was consensus regarding the importance of addressing evening routine, education regarding sleep processes, morning routine, and input regarding daytime activity, as core elements, and suggestions on how to address these domains were aligned.

Findings suggested many domains were better to address early. This may suggest a design of intervention with more input weighted toward the front. The time required for behaviour change and circadian rhythm change, equally suggests allowing time for clients to put new knowledge into practice. Emphasis on supportive therapeutic relationships, suggests some ongoing check-ins with the therapist for troubleshooting and support.

Interventions recommended appeared to be influenced by professional roles and accepted theoretical or moral stances, for instance in views around approaches to napping, time in bed restriction, and regularity of rise time. Participants who were CBTi practitioners emphasised these elements and suggested less flexibility here, and focused on homeostatic sleep drive and behavioural associations, which are core elements within CBTi theory [76]. Mental health experts, and particularly mental health OTs, emphasised personalisation and patient choice over various areas of the intervention (often including napping), perhaps in line with the predominant professional culture around client-centred practice [77, 78] and shifts toward shared decision making in mental health [79]. The formulation and presentation of the intervention should take account of the predominant professional culture of both those who will deliver the intervention, as well as other groups in the wider healthcare system.

Views diverged between professionals and those with personal experience regarding the ‘15 minute rule’ aspect of stimulus control therapy, although some professionals acknowledged some of the potential difficulty of this rule, others described it as fairly benign. It is indeed often included in self-help advice [80–83]. These negative experiences emphasise that how a component is delivered and received (interpretation and intention) is crucial to its effect. This finding may raise the possibility that this component could be differently received in people with SzSD than other groups, perhaps due to difficulties in regulation of arousal [84]. It would be interesting to know how and by whom this advice was delivered, and for how long and in what context the person tried it (e.g., during an acute exacerbation of sleep and mental health, or under more stable circumstances). These may be questions to address in future research focused on this component alone. We suggest that this aspect of stimulus control advice may not be useful as stand-alone self-help advice in this group.

Views on the appropriate contents of the intervention can be interpreted in terms of differing emphasis regarding which key mechanisms to focus on; some professionals emphasised sleep pressure and behavioural associations (more so recommending time in bed restriction and stimulus control), whilst others emphasised modulation of arousal (more so recommending relaxation and anxiety reduction), and still others emphasised circadian rhythm (more so recommending timed light exposure and other cues). These differing emphases reveal participant’s working hypotheses regarding the predominant factors which interfere with sleep in clients with SzSD. These different hypotheses are each supported by empirical evidence. Meta-analysis of passive monitoring studies shows high prevalence of hypersomnia in SzSD [2], and authors propose a role of maladaptive time in bed extension [85, 86]. A role of hyperarousal and of night-time worry in interfering with sleep has been supported in this group [87] and in general [88, 89], and both observational and basic science studies support a role of altered or reduced circadian response in SzSD [34, 90]. Thus, is it reasonable and not surprising that a number of distinct mechanisms might be targeted.

Readings of the evidence for light-based intervention varied widely among professionals, with some feeling there was very good evidence whilst others felt there was very little. This may relate to partial or incomplete awareness of the evidence, but our impression was that this was more due to differing epistemological perspectives regarding the evidence hierarchy [91]. Perhaps also different standards of proof were being sought, by some as though to recommend in policy, and by others only to be a good candidate for testing.

Another area where diverging views highlight a lack of directly relevant evidence was time in bed restriction in clients with SzSD, arguments for and against its safety and appropriateness were based on theoretical considerations or application of evidence from other groups. This points to a need for more studies of time in bed restriction in SzSD which assess or monitor safety (‘single component’ and ‘multi-component’ formulations may both be informative).

Which factor(s) to focus on first, or most, can potentially be completely determined in response to each client’s individual problems and presentation, and some advocated this. However, there was a potential trade-off identified between the extent of personalisation and increasing complexity. The amount of therapist procedural knowledge required (when-then rules) may be increased by such personalisation, which each require repetition to become automatic. Prior to this procedural knowledge being easily accessed, interpersonal skills are adversely affected [92]. Thus it is a consideration that therapist capacity for listening, use of self, and fostering alliance [93, 94], could actually be adversely affected by a protocol that allows too much personalisation.

Furthermore, presenting options and choices to clients is not always experienced as positive, and can be debilitating, leading to excessive delay of decisions, avoidance of regret, and a feeling of forced responsibility—clients want information, and the choice of a practitioner they trust, but vary in their preferences for role and control within clinical decisions [79]. A particular area where the prioritisation of presenting a clear message versus offering choice is pertinent is time in bed restriction; by its nature time in bed restriction is challenging [95], but through its rigorous application can reduce sleep effort, dysfunctional sleep beliefs, and insomnia [96]. Our findings described the view that time in bed restriction must be delivered confidently to be effective, but we also found many participants are concerned about the risks of time in bed restriction.

This poses a potential challenge. If therapists attempt to deliver time in bed restriction, but do so too deliberatively and cautiously, this may increase rather than reduce sleep effort and excessive concern about sleep; as therapist anxiety can interact with that of the patient [97]. This phenomenon has been found in exposure therapy; where therapists who were too cautious, allowing safety behaviours, offering many options, and terminating tasks too soon, produced poorer treatment effects, and even increased fear sensitisation [98]. Similarly, in relation to daytime activity plans, participants with personal experience, emphasised they didn’t want things to be too vague to give them the opportunity to talk themselves out of things. This highlights that responsiveness to the patient should not mean changing course at the first sign of difficulty, or at any expression of resistance or reluctance.

There are a number of ways an intervention can be responsive to the individual. Personalisation may be driven solely by individual choice (as with ‘personalisation of care’ with individual budgets), or by identification of biomarkers or phenotypes which respond differently to different treatments. Both types of personalisation were discussed in these results. The former taken too far can be criticised for abdication of responsibility to individuals [99], whilst the latter alone might be too solely biological and neglect human factors [100].

Personalisation can be via an adaptive protocol with different plans for different scenarios, or personalisation can involve more free-form clinical reasoning of the individual therapist. The latter is described in some occupational therapy literature as a *feature* of client-centred practice, with authors describing the ‘artistry’ of clinical judgement, involving creativity and intuition, and very much a non-standardised approach [77, 78]. Personalisation advocated within these results includes the biologically driven, such as to account for interindividual variability in phase-delaying response to evening light [101], and also the less biological, involving intuitive crafting of some aspects of the intervention plan to suit the individual’s unique circumstances and priorities. It is our challenge to accommodate both; they can be compatible. If occupational therapy is aligned with romanticism rather than empiricism [97] this might suggest a difficulty of implementing a protocol based therapy, involving biological mechanisms, as well as personal and contextual client factors. Occupational therapists have however also expressed alignment with empiricism [102], and have specifically described the desire for more research and scientific evidence based techniques to use in relation to sleep [35]. Further, many of the strongest advocates for a protocol-based approach within this study were sleep OTs.

Another approach to personalisation beyond the scope of current work but which could usefully be studied in future would be personalisation of sleep intervention by selection of a different therapy protocol/pathway and lead professional, based on screening or clinical assessment. Our findings suggest which areas are best suited to delivery by an OT (for instance routine and activity), some better address by a clinical psychologist or psychological therapist (such as nightmares and trauma) and some which required medical input (such as medication). We have developed a stand-alone intervention deliverable by a single OT with access to supervision, partly for ease of testing and implementation against a background of limited current sleep expertise in services. However, we would equally be keen to see the development of an adaptive MDT delivered therapy protocol embedded in a rigorous process of co-design with clinician and stakeholder input.

### Limitations

We have not deemed it ethical to separately report on the views of participants who personally deliver adapted CBTi to participants with SzSD due to this being a very limited pool of experts internationally. Whilst it might have been interesting and informative to examine these views separately, our duty not to make individuals potentially identifiable over-rode this.

Typically health-related expert opinion Delphi studies recruit a smaller sample, all with similar expertise [63] and do not attempt to triangulate between groups. The diverse experience within this study is both a strength, as it addresses this topic in which various groups meet, and a limitation as it made the variation in responses more complex to analyse and interpret. Similarly, we included service users and carers together in the final stage of data collection, and largely analysed these views together, as they were sought to answer the same aims with carers giving proxy reports. Separate analysis appeared to further fragment the data and did not suggest any important differences, but we acknowledge that service user and carer views are not equivalent.

The rating of daytime activity as ‘core’, may of course have been influenced by the lead researcher and interviewer being an OT, thus those who felt activity and occupation were less relevant, or that OTs were an inappropriate profession to deliver sleep interventions might not have volunteered to participate, or may have increased their endorsement of the importance of occupation and activity through social acceptability bias [103, 104]. This is a potential limitation, although efforts were made to reduce bias through encouraging honest responses, and through use of an online survey which is associated with less bias than in person survey or interview [103].

It is acknowledged that the possibility of bias in selection of experts [105] cannot be completely removed, however the diverse views represented hopefully gives some reassurance that participant selection was not biased by any desired outcome other than seeking relevant knowledge to inform intervention development.

We did not obtain any views from mental health nurses, who represent a large part of the workforce and include many individuals with relevant expertise. With hindsight, some of our mental health clinician and researcher experts should have been drawn from mental health nursing and had we set a sub-group target this would clearly have been achievable. We acknowledge limited representation of views from professionals with Asian and African residence, and our inability to include non-English speaking participants limits transferability.

Although we were able to recruit 24% non-White-British participants with personal experience, representation of reversed or very delayed sleep and hypersomnia may have been limited by the daytime nature of the group (10:30am-3pm). Previous work offering individual interviews in a location of participant choice included many more participants with these difficulties [20]. The group setting is also less likely to attract participants who are reluctant to disclose a psychosis related diagnosis, thus excluding those who experience most diagnosis related stigma or self-stigma [106]. Although we hoped to include by-proxy the views of service users who may struggle to attend by inclusion of carers, in hindsight adding options for remote or delayed participation could have improved inclusiveness.

Finally, it is a limitation that we stopped after a predetermined number of survey rounds (three), when there was more we could usefully have asked; but at the same time, we assume it would have negatively impacted our ability to recruit and retain the desired volume and quality of participants if length was not pre-determined.

## Conclusion

Participants felt intervention by mental health occupational therapists to improve sleep in people with SzSD was potentially feasible and worthwhile, and they were exceptionally willing to contribute time and energy to the development of such an intervention. They almost entirely agreed on the inclusion of a few core elements within this intervention, whilst views were mixed on other elements, and on the most appropriate emphasis within the intervention. There was no consensus on the *extent* of personalisation to accommodate, or on and *what aspects* of the intervention to personalise in response to individual circumstances and needs. Similarly, views varied on how and where to offer patient choice on therapy approach and format. It was agreed though that the intervention must personalise, whilst avoiding excess complexity.

Suggestions regarding how to address activity, routines and environment were very congruent within and between groups, and the approaches described were very compatible with the existing approaches of mental health occupational therapists. Occupational therapists have previously suggested that some of their core skills can effectively be repurposed to deliver behavioural sleep interventions, and our findings, based on a far wider group of stakeholders, tend to confirm that view.

## Supporting information

S1 File Data to support findings presented. Qualitative data excerpts and graphed results of multiple choice and Likert responses, organised by topics and themes.
(DOCX)

S2 File Round 1 survey questions. (PDF)

S3 File Round 2 survey questions. (PDF)

S4 File Round 3 survey questions. (PDF)

S5 File Topic guide for focus groups. Used in stage 4 with participants with relevant personal experience.
(DOCX)
