# Supplementary material for: A mixed methods expert opinion study on the optimal content and format for an occupational therapy intervention to improve sleep in schizophrenia spectrum disorders
Source: PLoS One. 2022 Jun 6;17(6):e0269453. doi: 10.1371/journal.pone.0269453 (PMC9170103; doi:10.1371/journal.pone.0269453)
Supplement: S1 File — Qualitative data excerpts and graphed results of multiple choice and Likert responses, organised by topics and themes. (DOCX) [file pone.0269453.s001.docx]

**S1 Supporting Information. Data to support findings presented.**

Qualitative data excerpts and graphed results of multiple choice and Likert responses, organised by topics and themes

Notes to the reader:

The number of participants voicing the qualitative content represents how many specifically expressed this at least once. In some cases participants were asked their view on this topic by the survey or during discussion, and in some cases they brought up this topic themselves. Participants not counted among those expressing any given view does not necessarily mean they did not hold this view but that they did not explicitly express this in their qualitative data. Where for a thematic code a group is listed as ‘n/a’, this reflects that this group was not asked about this topic and/or that their responses were not coded regarding this topic.

Apparently opposing views listed below are not always mutually exclusive, often participants did express both views, and this content was coded and counted thus. In many such cases participants acknowledged a tension and a balance to be struck.

This content is presented in roughly the order of the findings in the manuscript, however it is noted that there are of course some overlaps and links between themes. Quantitative data summaries on the same or related topics and questions are inserted throughout.

**Overview of topic areas and themes:**

[1. Intervention targets and scope 7](#_Toc58406403)

[Sleep problems and sleep interferers 7](#_Toc58406404)

[Sleep effort and frustration 7](#_Toc58406405)

[Worry, rumination, stress and anxiety 7](#_Toc58406406)

[Psychotic symptoms 8](#_Toc58406407)

[Fear of the dark 8](#_Toc58406408)

[Fear of silence 8](#_Toc58406409)

[Fear of the bed 8](#_Toc58406410)

[Fear of sleep 8](#_Toc58406411)

[Long sleep. 8](#_Toc58406412)

[Difficulty rising & sleep inertia 8](#_Toc58406413)

[Physical illness / physical symptoms 9](#_Toc58406414)

[How far to address ‘other’ sleep disorders 10](#_Toc58406415)

[Screen for sleep disordered breathing (SDB) and parasomnias 10](#_Toc58406416)

[Nightmares 10](#_Toc58406417)

[Assess nightmares 10](#_Toc58406418)

[Directly address nightmares specifically 10](#_Toc58406419)

[Nightmares may improve through treating sleep 10](#_Toc58406420)

[Refer on regarding nightmares 10](#_Toc58406421)

[Stability 11](#_Toc58406422)

[How well or stable would clients need to be to benefit? 11](#_Toc58406423)

[Stability of social situation important 12](#_Toc58406424)

[Stability of medication important 12](#_Toc58406425)

[Concerns about exclusions 12](#_Toc58406426)

[Transdiagnostic intervention? 13](#_Toc58406427)

[The intervention should be applied trans-diagnostically 13](#_Toc58406428)

[The intervention should focus exclusively on people with a schizophrenia spectrum diagnosis within this study as they are harder to reach 13](#_Toc58406429)

[2. The Assessment 14](#_Toc58406430)

[Format & manner of assessment 14](#_Toc58406431)

[Use an interview 14](#_Toc58406432)

[Use checklists and/or standardised questionnaires 14](#_Toc58406433)

[Rapport in assessment 14](#_Toc58406434)

[Prioritisation of areas to assess 15](#_Toc58406435)

[Longitudinal self-report of sleep & activity (activity & sleep diary) 17](#_Toc58406436)

[Sleep diary 17](#_Toc58406437)

[Activity diary 17](#_Toc58406438)

[Diary burden & difficulties 17](#_Toc58406439)

[Completing diaries as an intervention 17](#_Toc58406440)

[Format options, prompts and support 17](#_Toc58406441)

[Possibility of using an app 17](#_Toc58406442)

[Passive monitoring within the assessment 19](#_Toc58406443)

[Self-report and passive monitoring results will differ (useful to compare / need both) 19](#_Toc58406444)

[Passive monitoring as an intervention 19](#_Toc58406445)

[Measurement of light exposure 22](#_Toc58406446)

[Measurement or self-reporting of light exposure at baseline and during intervention 22](#_Toc58406447)

[3. Intervention components 23](#_Toc58406448)

[Sleep schedule 23](#_Toc58406449)

[Address sleep schedule regularity 23](#_Toc58406450)

[Regular rise time 23](#_Toc58406451)

[Regular bedtime 23](#_Toc58406452)

[Allowable flexibility in sleep schedule 23](#_Toc58406453)

[Need to fit sleep in with life 23](#_Toc58406454)

[It might be OK to be nocturnal 23](#_Toc58406455)

[Gradual approach to sleep timing changes 24](#_Toc58406456)

[Stabilise timing first before changing times 24](#_Toc58406457)

[Support to change sleep times 24](#_Toc58406458)

[Time in Bed restriction 27](#_Toc58406459)

[Advocating Sleep Restriction Therapy (SRT) 27](#_Toc58406460)

[Be cautious with SRT 27](#_Toc58406461)

[SRT could trigger mania / psychosis 27](#_Toc58406462)

[Do not use SRT 27](#_Toc58406463)

[Use sleep compression instead of SRT 27](#_Toc58406464)

[Not keen to try reducing time in bed 27](#_Toc58406465)

[Already reduce time in bed, & advocate it 27](#_Toc58406466)

[Napping 30](#_Toc58406467)

[Allow napping 30](#_Toc58406468)

[Avoid napping 30](#_Toc58406469)

[Evaluate naps 30](#_Toc58406470)

[Nap duration 30](#_Toc58406471)

[Nap timing 30](#_Toc58406472)

[Replace naps with activities 30](#_Toc58406473)

[Schedule naps 30](#_Toc58406474)

[Stimulus control, and managing awakenings 33](#_Toc58406475)

[Avoid non-sleep activities in bed / bedroom 33](#_Toc58406476)

[Use ‘the 15 minute rule’ or similar 33](#_Toc58406477)

[Bad experience using ‘the 15 minute rule’ as self-help advice 33](#_Toc58406478)

[Address activities to do if you awaken in the night 34](#_Toc58406479)

[Provide education on awakenings being normal 34](#_Toc58406480)

[Morning routine 36](#_Toc58406481)

[Address type of activities 37](#_Toc58406482)

[Use of alarms 37](#_Toc58406483)

[Dawn simulator alarms 37](#_Toc58406484)

[Education on sleep inertia 37](#_Toc58406485)

[Experience of struggle with waking 38](#_Toc58406486)

[Evening routine 40](#_Toc58406487)

[Evening wind down activities, lower stimulus 41](#_Toc58406488)

[Preparation for bed before wind down 41](#_Toc58406489)

[Prepare for the next day - if relevant 41](#_Toc58406490)

[Support to find suitable activities 41](#_Toc58406491)

[Get ready for bed alarm 42](#_Toc58406492)

[Daytime activity 44](#_Toc58406493)

[Increasing amount of activity 44](#_Toc58406494)

[Address activity type 44](#_Toc58406495)

[Address activity timing 44](#_Toc58406496)

[Scheduling activities 44](#_Toc58406497)

[Routines and habit formation 44](#_Toc58406498)

[Meaning, satisfaction and enjoyment 44](#_Toc58406499)

[Support to find and plan activities 44](#_Toc58406500)

[Addressing medications 47](#_Toc58406501)

[Consider side effects 47](#_Toc58406502)

[Addressing timing of prescribed medications 47](#_Toc58406503)

[Addressing food and drink 49](#_Toc58406504)

[Consider food and drink timing 50](#_Toc58406505)

[Address avoiding late eating 50](#_Toc58406506)

[Address skills and/or routines around meals 50](#_Toc58406507)

[Night eating 50](#_Toc58406508)

[Consider food and drink content 51](#_Toc58406509)

[Addressing substance use 53](#_Toc58406510)

[Substance use 54](#_Toc58406511)

[Alcohol 54](#_Toc58406512)

[Caffeine 54](#_Toc58406513)

[Nicotine 54](#_Toc58406514)

[Light Exposure 55](#_Toc58406515)

[Modifying light exposure 55](#_Toc58406516)

[Timing of modifications to light 55](#_Toc58406517)

[Morning light exposure 55](#_Toc58406518)

[Daytime light exposure 55](#_Toc58406519)

[Increasing evening light 55](#_Toc58406520)

[Reducing evening light exposure 55](#_Toc58406521)

[Reducing light at night 55](#_Toc58406522)

[Method to modify light 56](#_Toc58406523)

[Light box 56](#_Toc58406524)

[Light visor 56](#_Toc58406525)

[Blue-blockers / amber glasses 56](#_Toc58406526)

[Modifying light in the home & bedroom 56](#_Toc58406527)

[Using outdoor light / natural light 56](#_Toc58406528)

[Season is important 57](#_Toc58406529)

[Embedding light in activity / occupation 57](#_Toc58406530)

[Education regarding light, circadian rhythm and mood 57](#_Toc58406531)

[Low expectation of efficacy regarding light 57](#_Toc58406532)

[Acute alerting effects of light 57](#_Toc58406533)

[Environmental assessment and intervention 60](#_Toc58406534)

[Home environment 60](#_Toc58406535)

[Bed or sleeping surface 60](#_Toc58406536)

[Bedroom / bed not for non-sleep activities 60](#_Toc58406537)

[Having other useable rooms 60](#_Toc58406538)

[Noise in the bedroom 60](#_Toc58406539)

[Temperature in the bedroom 60](#_Toc58406540)

[Air quality 61](#_Toc58406541)

[Sensory factors 61](#_Toc58406542)

[Pets in the bedroom 61](#_Toc58406543)

[Home environment intervention 61](#_Toc58406544)

[Feeling safe in the home 61](#_Toc58406545)

[Social environment & context 61](#_Toc58406546)

[Social environment in the home 61](#_Toc58406547)

[Support from friends, family and carers 62](#_Toc58406548)

[Social commitments 62](#_Toc58406549)

[Peer support 62](#_Toc58406550)

[Loneliness 62](#_Toc58406551)

[Cultural factors 62](#_Toc58406552)

[Relaxation and / or mindfulness 64](#_Toc58406553)

[Relaxation techniques 65](#_Toc58406554)

[Breathing techniques 65](#_Toc58406555)

[Mindfulness meditation 65](#_Toc58406556)

[Thermoregulation 68](#_Toc58406557)

[Thermoregulation 68](#_Toc58406558)

[Addressing sensory factors 70](#_Toc58406559)

[Cognitive or psychological approaches 71](#_Toc58406560)

[Cognitive or psychological approaches 71](#_Toc58406561)

[Psychological approaches better dealt with by psychological therapist 71](#_Toc58406562)

[4. Personalisation 74](#_Toc58406563)

[The goals of the intervention should be individually determined 74](#_Toc58406564)

[The methods of intervention should be personalised 74](#_Toc58406565)

[Limits to personalisation 75](#_Toc58406566)

[5. Format, structure and pragmatic considerations 76](#_Toc58406567)

[Personalisation and complexity vs simplicity to deliver 76](#_Toc58406568)

[Personalisation 76](#_Toc58406569)

[Keep it simple 76](#_Toc58406570)

[Format of intervention and assessment materials 77](#_Toc58406571)

[Format options & literacy 77](#_Toc58406572)

[Use of technology in delivery of the intervention 77](#_Toc58406573)

[Core vs optional components 78](#_Toc58406574)

[Core vs optional components 78](#_Toc58406575)

[Order of delivery 79](#_Toc58406576)

[Order of delivery 79](#_Toc58406577)

[Follow up and ending of therapy 81](#_Toc58406578)

[Maintenance plan 81](#_Toc58406579)

[Follow up / tapering of ending 81](#_Toc58406580)

[6. Therapeutic approach and therapist factors 82](#_Toc58406581)

[Therapeutic approach, therapist attitude & manner 82](#_Toc58406582)

[An educational approach 83](#_Toc58406583)

[Education re: normal sleep 83](#_Toc58406584)

[Normalising 83](#_Toc58406585)

[Experimentation 83](#_Toc58406586)

[Benefits of change, motivational interviewing approach 83](#_Toc58406587)

[Therapeutic rapport & listening 84](#_Toc58406588)

[Rapport required before home assessment 84](#_Toc58406589)

[Therapist knowledge, skills & confidence 85](#_Toc58406590)

[Therapist confidence in delivering the intervention 85](#_Toc58406591)

[Relationship to OT role & skills 85](#_Toc58406592)

[Generic working barrier to OT interventions 85](#_Toc58406593)

[7. Implementation considerations 86](#_Toc58406594)

[Reaching referrals 86](#_Toc58406595)

[Reaching referrals 86](#_Toc58406596)

[MDT approach within intervention 86](#_Toc58406597)

[MDT knowledge & attitude 87](#_Toc58406598)

[MDT approach to intervention 88](#_Toc58406599)

[MDT approach to medication 88](#_Toc58406600)

[MDT approach to maintenance 88](#_Toc58406601)

# Intervention targets and scope

## Sleep problems and sleep interferers

| Recommendation or issue raised | | Data excerpts | professionals  n= (%) | personal exp.  n= (%) |
| --- | --- | --- | --- | --- |
| Sleep effort and frustration | | “It doesn’t work. In fact, it makes it worse and then you get angry.” (personal experience)  “you think, I’ve got something to do tomorrow just sleep, you are forcing yourself to sleep and then you can't sleep then. I have that.” (personal experience)  “if I was to say, go and read a book for 15 minutes or just listen to some music, it just wakes me up completely. Whereas like I can still get a couple of hours sleep by staying there and I like “go to sleep, go to sleep”.” (personal experience)  “try and lie still” (personal experience)  “…the more we focus on it the more we get worked up” (sleep OT clinician)  “…when I asked them how they utilise relaxation, it is clear that the use as a bludgeon to try to knock themselves into unconsciousness. […] So we need to drop all of that it’s a therapy of learning to let go and give up.” (CBTi therapist) | 10 (18%) | 4 (15%) |
|  |  | A minority of people with personal experience described sleep effort and frustration, with some passion. One person however explicitly described sleep effort being potentially effective, whilst others described similar sentiments around ‘just lie still’ and advocating staying in bed for long periods even if not asleep.  Professionals varied in their focus on reducing sleep effort, with it being referred to as central by some and not explicitly mentioned by others. | | |
| Worry, rumination, stress and anxiety | | “No, you just lay there going through the day and just everything just swirls around in your head.” (personal experience)  “I know that I’m going to have nightmares, and then obviously your thoughts start racing thinking about it” (personal experience)  “Clear mind at night don't over think.” (personal experience)  “No amount of tinkering with the environment will help if the individual's sleep is disrupted by worry. This has to be addressed if it is an issue” (OT CBTi therapist)  “Treating rumination [suggested component]” (psychiatrist)  “Not in-depth, unless the primary cause of insomnia is rumination, in which case why not refer to a specialist.” (MH OT) | 23 (41%) | 18 (69%) |
|  |  | People with personal experience described worry and rumination both about sleep or unrelated to sleep, both as a cause of sleep disruption and as what happens if they cannot sleep. Professionals gave varied amounts of focus to addressing worry, usually acknowledging its impact, but varying regarding to what extent they felt addressing this directly should be within the scope of this intervention (discussed below).  This links to the discussions (below) of whether and when are psychological aspects better referred to a psychological therapist. | | |
| Psychotic symptoms | | “relationship between nightmares and delusions. Determine they are really nightmares not merely delusions” (sleep OT)  “… because of the negative symptoms, and just being in the sick role, losing that structure to the day, that’s why they have become bad sleepers and that’s quite difficult [to work with]” (MH OT)  “[voices] that’s what keeps me up some nights.” (personal experience) | 11 (20%) | 4 (15%) |
| Sleep related fears | Fear of the dark | “Some patients may sleep better if there is some light if it makes them feel safe.” (sleep specialist psychiatrist)  “like dark bedroom, many pts are afraid in the night and have the lights on so try to help them with that.” (circadian rhythm & MH clinician researcher)  “Yeah, I still go to sleep in the pitch black. […] Which is stupid because I’m scared of the dark.” (personal experience) | 2 (4%) | 2 (8%) |
|  | Fear of silence | “I listen to the radio in mine. I never switch it off. […] Well, I’m afraid of the silence, you know [inaudible]. In a way it’s a distraction” (personal experience) | 1 (2%) | 1 (4%) |
|  | Fear of the bed | “scared to go to sleep in their bed as they had associated it with voices” (sleep & MH researcher) | 5 (9%) | 0 (0%) |
|  | Fear of sleep | “People who have nightmares can often get to dread sleep (as might someone with sleep terrors).” (sleep OT)  “fear of sleeping due to paranoid delusions an issue in some, but not many, patients.” (sleep specialist psychiatrist) | 3 (5%) | 0 (0%) |
| Oversleeping | Long sleep | “I have experience of clients sleeping 12-16 hours on these medications [Olanzapine and Clozapine].” (psychiatrist)  “you might get people feeling they need 11hrs but then that might be because it is such poor quality.” (MH sleep researcher)  “so that’s a whole work week extra you are spending on sleeping that you could be spending on something else” (psychiatrist)  “Sometimes I sleep like 15 hours if nobody wakes me […] It really worries him that I’ve actually passed away in my sleep” (personal experience) | 27 (48%) | 4 (15%) |
|  | Difficulty rising & sleep inertia | “even if you get them up and standing, they will have any recollection of what happened to them though being a trancelike state” (CBTi therapist)  “I would be worried about the morning part […] Because I struggle to wake up […] even for alarms and stuff.” (personal experience) | 21 (38%) | 8 (31%) |
|  |  | People with personal experience described worry and rumination both about sleep or unrelated to sleep, both as a cause of sleep disruption and as what happens if they cannot sleep. Professionals gave varied amounts of focus to addressing worry, usually acknowledging its impact, but varying regarding to what extent they felt addressing this directly should be within the scope of this intervention (discussed below).  This links to the discussions (below) of whether and when psychological aspects are better referred to a psychological therapist. | | |
| Physical illness / physical symptoms effect on sleep | | “physical setup of the mattress, pillows etc pertaining to physical injuries/pain or biomechanical support needs to improve comfort.” (sleep OT)  “menopause, hot flashes…” (sleep OT)  “what keeps you awake? […] If it’s worries it’s worries, if it’s pain it’s pain.” (sleep specialist psychiatrist)  “ [naps] Very dependent on individual's situation/needs/co-morbidities/medication” (sleep OT) | 12 (21%) | 4 (15%) |

Please note the above are shortenings of how the questions were phrased.

Falling asleep without getting stressed was highly endorsed, which relates well to comments made about worry and rumination. Other priorities which were most highly endorsed relate a lot to daytime functioning (first 4 listed, plus ‘sleep in the night not in the day). These were followed by priorities around sleep maintenance. Sleeping within less than 15 minutes was by no means a universal priority.

Only limited time was devoted to the topic of what aspects of sleep were a priority to treat as previous work has explored this .

## How far to address ‘other’ sleep disorders

| Recommendation or issue raised | | Data excerpts | professionals  n= (%) | personal exp.  n= (%) |
| --- | --- | --- | --- | --- |
| Screen for sleep disordered breathing (SDB) and parasomnias | | “OSA/PLMD/RLS/parasomnia, the practitioner should have a basic understanding of these conditions, and be able to screen for them, and refer on if likely to be a problem” (sleep & MH researcher) | 21 (38%) | 1 (3%) |
|  |  | Participants suggested screening for SDB and parasomnias in round 1, and it was accepted as consensus by round 2. We did not ask in the Nominal Group Technique stage / final stage (participants with personal experience) if we should screen for these but one person brought it up. | | |
| Nightmares | Assess nightmares | “how often these occur and how distressing they are” (MH OT) | 37 (66%) | 0 (0%) |
|  | Directly address nightmares specifically | “self-soothing techniques to address emotional dysregulation following nightmares” (sleep OT clinician)  “Education on how you should not hold off your sleep in fear of having a nightmare as this can increase the likelihood that you experience a nightmare when falling asleep (REM rebound)” (OT CBTi therapist)  “strategies for dealing with nightmares - e.g. rescripting, grounding” (MH & sleep clinical academic) | 16 (28%) | 0 (0%) |
|  | Nightmares may improve through treating sleep | “Our CBTi […] resulted in decreased nightmares without specific "rescripting" interventions.” (OT CBTi therapist) | 6 (11%) | 0 (0%) |
|  | Refer on regarding nightmares | “Access to a clinician specialised in treating trauma” (MH & sleep clinical academic)  “I feel they should be treated differently and receive something special for dealing with nightmares.” (circadian rhythm researcher)  “I don’t get into dream content. […] Yes I don’t feel comfortable doing that.” (sleep OT clinician) | 13 (23%) | 0 (0%) |
|  | There was a consensus to assess regarding nightmares, with some variation regarding importance and time to devote to this.  Where it came to directly addressing nightmares, some suggested education, or developing strategies similar to for how to manage awakenings for other reasons, when rated in round 2 this was rated mostly as important or very important.. Some suggested use of specific nightmare interventions such as imagery rehearsal. Some felt this could be delivered by the therapist, and some suggested referral onward. The possibility of trauma and increased suicidal risk were mentioned in relation to nightmares. | | | |

## Stability

| Recommendation or issue raised | Data excerpts | professionals n= (%) | personal exp. n= (%) |
| --- | --- | --- | --- |
| How well or stable would clients need to be to benefit? | “Mental health needs to be settled (non-acute) at the time the patient enters the programme” (sleep OT clinician)  “[Too unwell would be] Too thought disordered to understand the purpose of the intervention or the instructions.” (sleep specialist psychiatrist)  “A lot of people who have high levels of residual symptoms are perfectly capable of understanding…” (sleep & MH researcher)  “If they are unable to attend to conversation with clinician, retain information and implement suggested interventions” (sleep OT clinician)  “…"acutely unwell" suggests the patient has been better relatively recently, and is likely to become less symptomatic in the near future, so this would suggest there would be a better time to offer the therapy.” (psychiatrist)  “[With severely ill patient] then I would suggest the simple … like starting with light, and dark bedroom is a simple thing.” (sleep specialist psychiatrist) | 48 (86%) | n/a |
|  | A few participants who did not work in specialist mental health settings suggested the patient should be completely ‘well’ in other respects before sleep treatment commenced, however the predominant view was to keep any such threshold low. Factors which would present too much of a barrier were around the ability to attend to and take in information and carry out recommendations, as well as regarding suicidal risk and risk to others.  It was noted that those who were more acutely unwell might benefit from environmental interventions, or more intensively supported behavioural interventions, but that this might be better as a separate intervention. | | |
| Stability of social situation important | “If the person is in very temporary accommodation or sleeping on a friends’ couch or something temporary, it would be better to start the intervention when they are in more stable accommodation” (sleep OT clinician) | 5 (9%) | n/a |
| Stability of medication important | “…change of anti-psychotic medication, it would be desirable for this to be done before the start of treatment” (sleep & MH clinical academic) | 6 (11%) | n/a |
| Concerns about exclusions | “I think if the person has issues with sleep and wants to engage they should have the opportunity to.” (MH OT)  “…a sleep intervention could actually be a positive step in starting their recovery.” (sleep & MH researcher) | 4 (7%) | n/a |
|  | Professionals were prompted to describe concerns with having exclusion criteria around stability or wellness but few were described. What was said links to suggestions above of potential for a separate intervention designed for during acute illness. | | |

Agreement converged toward ‘agree’, away from strongly disagree, and with fewer participants expressing ‘neutral’ opinion. This combined with the qualitative data suggests participants converged toward a view that there were some aspects or degree of stability that were required to make this kind of intervention practicable, but that it could be applied fairly broadly.

## Transdiagnostic intervention?

| Recommendation or issue raised | Data excerpts | professionals  n= (%) | personal exp.  n= (%) |
| --- | --- | --- | --- |
| The intervention should be applied trans-diagnostically | “Similar considerations would be relevant for various groups” (sleep researcher)  “It is a bit "medical-model" to focus on the diagnosis itself, though I understand that the interventions should be tailored based on research to specific situations” (sleep OT clinician) | 35 (62%) | n/a |
|  | Participants raised that the intervention would be relevant trans-diagnostically, so despite that our initial aim was to develop it for people with schizophrenia spectrum disorder diagnoses, we decided to ask participants in round 3 about this. | | |
| The intervention should focus exclusively on people with a schizophrenia spectrum diagnosis within this study as they are harder to reach | “Very relevant. And perhaps easier to implement - as someone also working specifically in sleep-psychosis, I can't help feeling that sz is the tougher end of the spectrum in which to initiate change.. But that of course doesn't mean that we should be trying.” (sleep specialist psychiatrist)  “I think you’ll have a much higher referral rate, if its non-schizophrenic, purely because practitioners will be weighing up whether they can participate and would want to… […] I’ve had quite a lot of people come round researching schizophrenia, and I’ve not been able to give them very many [referrals] …as a group of people, male especially, they tend to be less ‘on’ for participating in research than maybe some other groups of people.” (MH OT)  “My understanding is that the format of this intervention is designed to be more user-friendly and accessible to those with schizophrenia spectrum disorders, and those of other diagnoses could benefit greatly from this opportunity also.” (sleep & MH researcher) | 14 (25%) | n/a |
|  | Participants saw many advantages and arguments for applying the intervention trans-diagnostically, yet we stuck with testing the intervention in our narrower population. The researchers and PPI contributors and some participants felt that this group were more underserved in terms of sleep intervention, and might be harder to reach, thus we decided to maintain exclusive focus on this group in the feasibility study. | | |

# The Assessment

## Format & manner of assessment

| Recommendation or issue raised | Data excerpts | professionals  n= (%) | personal exp.  n= (%) |
| --- | --- | --- | --- |
| Use an interview | “patient perspective is key”  “Patient views”  “A few open questions to start […][then] need to ask very detailed specific questions”  “Detailed and skilled sleep history is the cornerstone of assessment.” | 27 (48%) | n/a |
| Use checklists and/or standardised questionnaires | “SDS-CL-25”  “e.g. PSQI, ESS”  “Brief screen for other sleep disorders eg Wilson et al would be realistic”  “OCAIRS”  “Interest checklist may be used.”  “social rhythm metric”  “DBAS”  “develop a sleep OT assessment kit”  “Horne Ostberg Morningness-Eveningness Questionnaire” | 31 (55%) | n/a |
|  | Various validated sleep and occupation measures were suggested, and non-validated checklists.  There was no indication of anyone suggesting this was instead of interviewing. | | |
| Rapport in assessment | “Behaviour change is hard and there is nothing as critical as an effective therapeutic relationship/rapport.” (sleep OT CBTi practitioner)  “listening to what is on the client's mind - it may take time”  “must emphasize good connection/good relation with the patient” (sleep specialist psychiatrist)  “I don't like strangers so interaction in the community first would be necessary” (personal experience)  “Home assessment would make me feel invaded.” (personal experience) | 5 (9%) | 6 (23%) |
|  | Some specifically advised or suggested how to improve rapport, whilst others might have assumed this was a given.  Rapport came up with those with personal experience specifically in relation to the idea of ‘home assessment’, which may not be acceptable to some, and may require building trust for others. The intention and focus of the assessment must be made very clear. | | |

## Prioritisation of areas to assess

Round 1 solicited short free text suggestions of assessment topics to cover. Answers were synthesised to produce these categories and rated in Round 2.

**Round 3 comments on the above:**

“Roughly agree with above ratings” (clinical psychologist)

“Looks good to me” (sleep & circadian rhythm researcher)

“I'm surprised medication isn't higher up. Its a significant factor in hypersomnia, which may maintain insomnia, and also a potentially modifiable factor (dose/timing). I would put this in the 'definitely include' category.” (sleep specialist psychiatrist)

“Even though most agree to not ask re family history of sleep complaints, I wonder if specific questions should be asked to rule out restless legs syndrome and other parasomnias (if you don't ask, people may not recognise to tell you about that; strong familial component; easily missed as major cause of sleep onset insomnia).” (sleep OT researcher)

Almost all suggestions were rated mostly as important or very important, posing the dilemma (acknowledged by some participants) that the assessment could become very long (see more below).

In anticipation of everything being rated important, we forced ranking of topics to attempt to distinguish the most important topics to prioritise (below). This resulted in the two clear less important areas, to receive less time or attention, although participants made persuasive arguments for the importance of these areas too. In conclusion on some topics, for some participants, it may be possible to move on sooner, whilst they may require more time for others.

Furthermore to shorten time needed, information on psychiatric condition and medication should be gathered from referrer or notes (also suggested by some participants).

## Longitudinal self-report of sleep & activity (activity & sleep diary)

| Recommendation or issue raised | | Data excerpts | | professionals  n= (%) | | personal exp.  n= (%) | |
| --- | --- | --- | --- | --- | --- | --- | --- |
| Sleep diary | “Informative but not crucial. You’d get a gauge from actigraphy.” (MH & sleep clinician researcher)  “relying on patient's self-report, so information may not be complete/accurate.” (sleep OT)  “could be helpful if done often” (personal experience) | | 25 (43%) | | 4 (15%) | |  |
| Activity diary | “activity and light paper logs […] to capture the activity being performed and whether an individual is inside or outside which can be very useful” (sleep & circadian rhythm researcher)  “Activity diary as people both over and underestimate the amount of activity they undertake…” (psychiatrist)  “I think it could add another layer of anxiety but it might be worthwhile (the data could be useful)” (personal experience) | | 16 (28%) | | 2 (8%) | |  |
| Diary burden & difficulties | “its important but may be tricky to get services user's to remember to do this.” (MH OT)  “[activity diary] laborious […] at times has also felt too confronting for them when they realise how little they do during they day” (MH & sleep clinician researcher)  “just remembering to do so” (personal experience) | | 21 (38%) | | 6 (23%) | |  |
| Completing diaries as an intervention | “Passive monitoring (actigraphy) may be okay, but having the client actively recording activities/sleep may better enhance personal awareness/understanding” (sleep OT CBTi practitioner) | | 25 (45%) | | 0 (0%) | |  |
| Format options, prompts and support | “In terms of high tech or low tech, I think people should be given an option.” (sleep OT)  “a call-in diary. Written activity day may be asking too much.” (sleep OT)  “…remind…” (professionals, 4 instances)  “Would need help from family member / friend. But would give it a go with help.” (personal experience) | | 24 (43%) | | 3 (12%) | |  |
| Possibility of using an app | “A tablet with preloaded apps where people can choose to write/ an icon will make daily diaries easier.” (MH OT)  “…routine and the diary, sleep diary but if you’ve got that app with the watch, I suppose you wouldn’t need to do it.” (personal experience) | | 7 (12%) | | 2 (8%) | |  |
| Overall most participants, professionals and those with personal experience alike, agreed that there were benefits to self-reported activity data above those of only passively recorded.  To reduce the challenge and motivation involved in remembering to record reminders should be used, and the process made as easy as possible.  One person with personal experience and one professional raised the possibility that the process could be anxiety provoking or distressing, but no-one suggested it should not be done for this reason. Most people were interested, or thought their clients would be interested, in seeing their data summarised. | | | | | | |  |

**Regarding completing self-reported activity and sleep diaries (NGT, personal experience):**

People with personal experience acknowledged / anticipated that recording one’s own activities and sleep timing over a period of time might be challenging, but they also saw value in how this data might be informative or empowering.

## Passive monitoring within the assessment

| Recommendation or issue raised | Data excerpts | professionals  n= (%) | personal exp.  n= (%) |
| --- | --- | --- | --- |
| Self-report and passive monitoring results will differ (useful to compare / need both) | “Objective and subjective assessments can be compared.” (MH OT)  “it is crucial to have this because an individual's impression of their patterns may be very different to what they are actually doing.” (circadian rhythm researcher)  One tricky thing though is when this doesn't match up with self-report […] put self-reported above when they don't match” (MH & sleep researcher) | 22 (39%) | 0 (0%) |
|  | Whilst the relative priority of objective and subjective measures varied between participants; overall there was consensus that both together and compared was optimum. Many mentioned the process of comparing these. | | |
| Passive monitoring as an intervention | “diary and actigraphy to have proof of their pattern - often it comes as a great relief to them to have proof of what they felt but because of low self-trust they doubted they were right” (sleep & MH researcher)  “to help service users understand how they spend their time could affect sleep” (MH OT)  “I don’t think the watch is going to work” (personal experience)  “…why I said on the watch I’d like to have feedback” (personal experience) | 16 (28%) | 5 (19%) |
|  | The feedback from the watch could have therapeutic value, particularly if it was accessible by the client between visits. Views on how useful this would be varied in those with personal experience. | | |

**Final stage, views from participants with personal experience: activity tracking wearables acceptability:**

**Duration of longitudinal measurement required (self-report and / or passive):**

During round 1 some suggested varying the baseline length based on what type of sleep problem is being assessed, we asked about this in rounds 2 & 3.


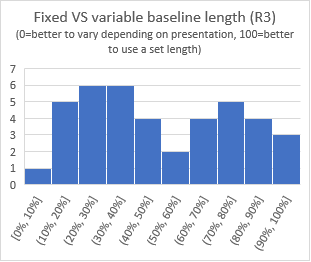

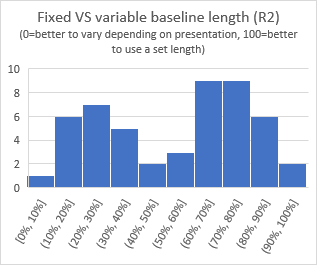


Professionals varied in how long they felt was needed to assess sleep pattern, with those working mostly in insomnia suggesting shorter periods and those in mental health and circadian researchers suggesting somewhat longer.

Overall professionals were more concerned about baseline period being too long than people with personal experience, who thought a longer period might be needed and were willing to wait.

“Pt's often want help immediately and already don't like to wait 2 weeks until the next appointment” (sleep OT)

## Measurement of light exposure

| Recommendation or issue raised | Data excerpts | professionals  n= (%) | personal exp.  n= (%) |
| --- | --- | --- | --- |
| Measurement or self-reporting of light exposure at baseline and during intervention | “Assessment of […] how the person’s current occupational routine results in exposure to light” (MH OT)  “would certainly want to know about first exposure to daylight and the general level of light and duration of exposure.” (sleep OT)  “Good experience with wrist-worn light sensor combined with actigraph, not much covering, can use rubber band and instruct carefully […] Most problematic during night-time, not valid because of blankets covering.” (MH & circadian rhythm clinician researcher)  “…an app that logs minutes looking at the phone screen), especially in the hours before bedtime, that could serve as a proxy for overall light exposure.” (MH researcher)  “The issue of light exposure is important, but measuring the light exposure accurately (e.g. by lux) doesn't seem as important as providing the education on how it can negatively impact sleep. I wouldn't sweat too much about accurate measurement (in lux) of light, unless you want to give a "teaching moment" to people.” (sleep OT)  “Important for people who don’t leave home often or have turned day into night and may allow them the insight into how this could be effecting their sleep” (MH OT) | 33 (59%) | 1 (4%) |

# Intervention components

## Sleep schedule

| Recommendation or issue raised | | Data excerpts | professionals  n= (%) | personal exp.  n= (%) |
| --- | --- | --- | --- | --- |
| Regularity | Address sleep schedule regularity | “Regular sleep times” (circadian rhythm researcher)  “routine may help to gain better sleep over time” (sleep OT)  “Routine may be helpful but may impact my social life” (personal experience) | 37 (66%) | 16 (62%) |
|  | Regular rise time | “getting up at pre-agreed time when slept badly” (sleep OT)  “Anchoring the rise time” (sleep specialist psychiatrist) | 25 (45%) | 15 (57%) |
|  | Regular bedtime | “Setting a bed time, is helpful to ensure the person doesn't sleep all the next day, particularly if they are a night owl.” (MH OT) | 9 (16%) | 2 (8%) |
| Flexibility | Allowable flexibility in sleep schedule | “Regular rise time is essential during Tx and can be flexed slightly in maintenance” (CBT-i practitioner)  “Schedule should allow for social participation/avoid isolation” (sleep OT)  “While we emphasize consistency, if sleep debt grows opportunity to pay it off should be included.” (sleep OT) | 12 (21%) | 0 (0%) |
|  |  | Some participants thought it was better not to mention possible flexibility too early so that people can adhere to regular times during therapy, others suggested it was important to stress that totally rigid times were not forever to gain buy in. | | |
|  | Need to fit sleep in with life | “An idea of how many odd days they can have a lie in/late night etc before sleep is affected (otherwise life gets very boring!” (psychiatrist)  “Goals for what they want their sleep to look like, possibly linked in to other things they want improving” (sleep & MH researcher) | 10 (18%) | 0 (0%) |
|  |  | Schedules shouldn’t remain so rigid that the person can’t have a social life. Sleep shouldn’t be at the expense of life. | | |
|  | It might be OK to be nocturnal | “but if you can’t sleep at night and you feel like you could sleep during the day, you need to grab that sleep when you can” (personal experience)  “There’s no law on it” (personal experience) | 0 (0%) | 2 (8%) |
|  |  | Although two participants discussed this suggestion they didn’t sound very happy with being nocturnal, but felt it was better than getting no sleep at all. | | |

| Recommendation or issue raised | | Data excerpts | professionals  n= (%) | personal exp.  n= (%) |
| --- | --- | --- | --- | --- |
| Timing changes | Gradual approach to sleep timing changes | “Small steps” (sleep OT)  “half an hour” (CBTi practioner)  “Slowly bring forward” (sleep specialist psychiatrist)  “Gradually adjusting wake/sleep rhythm […] making sure not to expose to light on wrong side of nadir” (MH & sleep researcher) | 11 (20%) | 0 (0%) |
|  | Stabilise timing first before changing times | “[Those with a] severe delay, they don’t have a clean severe delay, what they tend to be also is all over the place, and so it’s really hard at the beginning to determine what’s what […] So first get them stable […] [then] light exposure and start advancing that by half an hour a day” (CBTi practitioner) | 2 (4%) | 0 (0%) |
|  | Support to change sleep times | “I would need the support.” (personal experience)  “Might consider morning calls if the client indicates they will get up for someone else when they can not manage it for themselves” (MH OT) | 4 (7%) | 4 (15%) |

**Regular Rise time was mentioned in Round 1 and asked about in Rounds 2 & 3:**
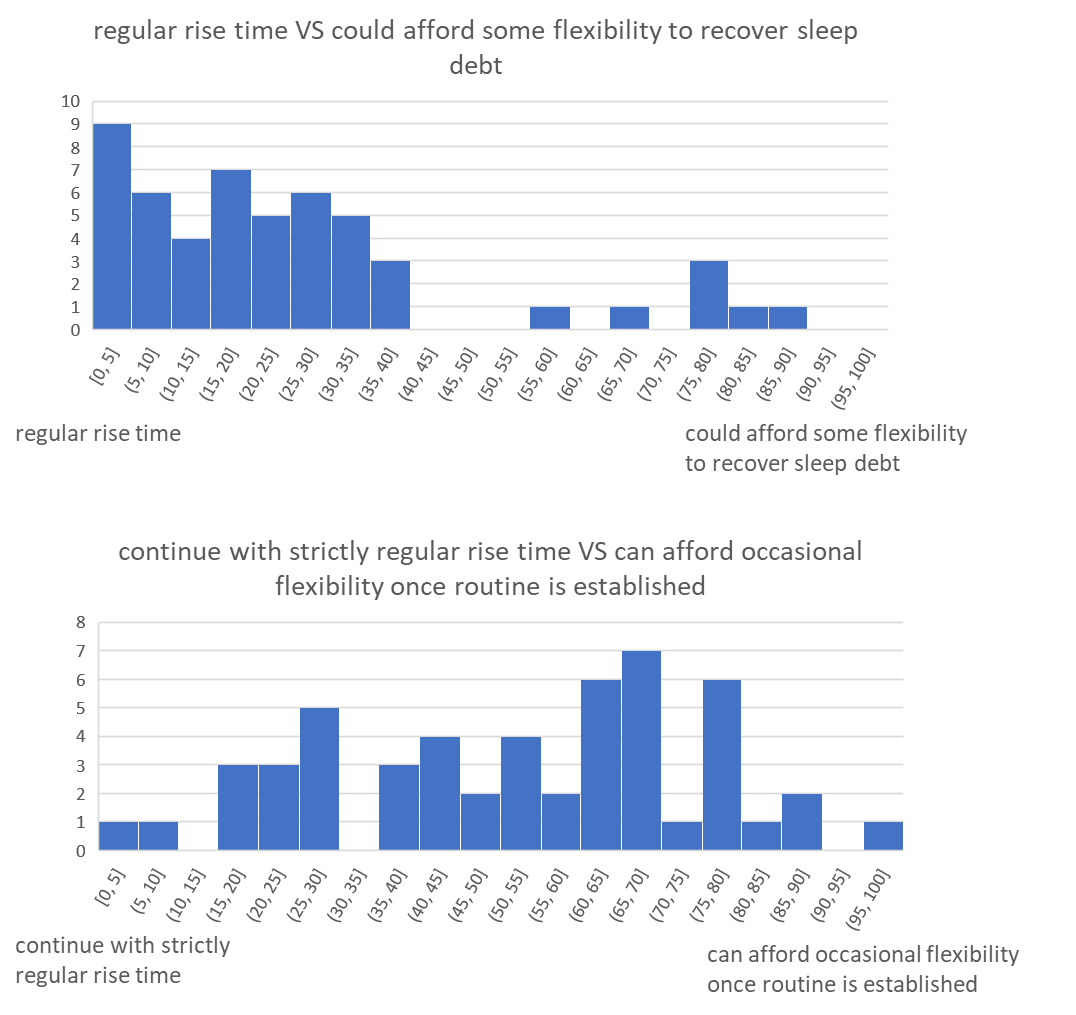


## Time in Bed restriction

| Recommendation or issue raised | Data excerpts | professionals  n= (%) | personal exp.  n= (%) |
| --- | --- | --- | --- |
| Advocating Sleep Restriction Therapy (SRT) | “Stimulus control is also effective as a mono-therapy. but sleep restriction with stimulus control is the BOMB!” (sleep OT CBTi practitioner)  “strong evidence base behind this component.” (sleep & MH researcher) | 17 (30%) | n/a |
| Be cautious with SRT | “Sleep restriction therapy only if the therapist has training and supervision” (MH & sleep researcher)  “Patients should be monitored though” (psychiatrist) | 18 (32%) | n/a |
| SRT could trigger mania / psychosis | “…i would be worried about using it in case it triggers them.” (sleep & MH researcher)  “Bear in mind that sleep deprivation has potential to trigger psychotic episode” (sleep & MH clinician researcher)  “Consider individual's symptoms and medication to evaluate whether sleep restriction etc. could exacerbate problems” (sleep researcher) | 16 (28%) | n/a |
| Do not use SRT | “sleep restriction therapy is probably contraindicated” (sleep OT)  “If one chooses to focus on light exposure/not blue light in late evening and night, there will presumably be no need for sleep restriction” (sleep & MH researcher) | 4 (7%) | n/a |
| Use sleep compression instead of SRT | “Sleep compression is appropriate if patient has any condition sensitive to short term sleep loss or if they are very anxious.” (sleep specialist psychiatrist)  “That is why I feel that sleep compression would be a good idea as there is literature that also backs using sleep compression with bipolar populations” (sleep OT CBTi practitioner) | 6 (11%) | n/a |
| Not keen to try reducing time in bed | “If I go to bed later, I’m up until 3am instead of 1am, you know what I mean. So I’ve got to go to bed at 21:00 just to unwind myself down.” (personal experience) | n/a | 5 (19%) |
| Already reduce time in bed, & advocate it | “I try to spend as little time as possible in bed.” (personal experience) | n/a | 3 (12%) |

Although sleep restriction was relatively highly endorsed during round 1 (52%=very appropriate, 83%=appropriate or very appropriate) ratings, views diverged by round 2 (only 62% suggesting to use some form of time in bed restriction). In round 3 a large minority selected the option ‘don’t know / no view’ (33%) when this option was offered, or abstained (4%), revealing then a larger majority of those with a view endorsing some form of time in bed restriction (91% / 31) and 8% / 3 suggesting it was better not to use time in bed restriction.

In the final stage participants with personal experience asked together about ‘Reducing excess time in bed and avoiding napping’, see below.

## Napping

| Recommendation or issue raised | Data excerpts | professionals  n= (%) | personal exp.  n= (%) |
| --- | --- | --- | --- |
| Allow napping | “Education about pros and cons, when is best or not, and strategies to integrate this into a sustainable routine if the person needs or likes to nap” (MH OT)  “Avoiding / reducing napping: would probably exacerbate” (personal experience) | 24 (43%) | 3 (12%) |
| Avoid napping | “Avoiding them where possible...!” (sleep & MH researcher)  “Ban it!” (sleep OT CBTi practitioner) | 19 (34%) | 2 (8%) |
| Evaluate naps | “It depends why the person is napping.” (MH OT)  “How often, what times, how long, do they feel refreshed, do they try to resist and what happens if they do.” (psychiatrist) | 27 (48% | 4 (15%) |
| Nap duration | “naps are ok if only 15-20 minutes” (sleep OT) | 18 (32%) | 2 (8%) |
| Nap timing | “What time does it occur - the later the more damaging it is.” (sleep specialist psychiatrist) | 11 (20%) | 0 (0%) |
| Replace naps with activities | “engage in physical activity and meaningful/productive activities rather than sleeping to promote engagement.” (sleep OT)  “Energising activities particularly important in hypersomnia population” (sleep & MH researcher)  “I think you need encouragement for that sometimes because you get [inaudible] very tired and unless you’ve got somebody in the house to say, let’s go for a walk or [inaudible] and if you haven’t got that then text reminders to encourage you” (personal experience) | 14 (25%) | 2 (8%) |
| Schedule naps | “Scheduled napping may be helpful in some cases” (sleep & MH researcher)  “nap to be taken 7-9hrs after habitual wake time in the typical circadian dip” (CBTi practitioner) | 6 (11%) | 0 (0%) |

**Final stage, views from participants with personal experience:**

(Reducing excess time in bed & avoiding napping, explained in terms of:

Sleep pressure is the drive to sleep

which builds up more the longer you have been awake.

Example (diagram) - reducing 12hrs sleep window (10pm-10am) down to 9hrs (12midnight - 9am)

Daytime naps reduce sleep pressure (balloon analogy)

• Avoid naps

• 1 short nap (under 30min)

• Not nap late in day

Explore the reasons people have for napping

Explore activities to replace naps with)

## Stimulus control, and managing awakenings

| Recommendation or issue raised | Data excerpts | professionals  n= (%) | personal exp.  n= (%) |
| --- | --- | --- | --- |
| Avoid non-sleep activities in bed / bedroom | “Modify to remove stimuli - like TV/computers etc... and make environment for sleeping and sex only” (MH OT)  “…where would they go. Is bed in same room. Can you set up separate zone etc.” (sleep & MH clinician researcher)”  “Respondent A: People say you shouldn’t have a television in the bedroom.  Respondent B: Yes, yes, it’s that unnatural…  Respondent C: You shouldn’t have anything electronic in the bedroom.” (personal experience)  “Like only go in your bedroom to sleep, that’s been quite helpful for me actually.” (personal experience) | 28 (50%) | 13 (50%) |
| Use ‘the 15 minute rule’ or similar | “I think it would be a bit harsh to set 15 minutes, because if someone took 20 minutes regularly, I wouldn’t be upset with that. (sleep & MH researcher)  “So if you’ve been in bed for a couple of hours its better to get out of bed and do an activity” (MH OT)  “I recommend if SRT is not used and stimulus control therapy is used there is still daily monitoring (sleep diary).” (sleep OT CBTi practitioner) | 8 (14%) | 0 (0%) |
|  | Some participants who deliver CBTi suggest using the 15 minute rule unmodified as a part of stimulus control to work with sleep restriction to establish the bed-sleep association, some who worked in mental health suggested milder versions, longer than 15 minutes or depending how the person is feeling in bed. | | |
| Bad experience using ‘the 15 minute rule’ as self-help advice | “It wakes you up.”  “Up, down, up, down, up, down. I find that if I just lie there and don’t move and don’t do anything whether I sleep or not and I stay like that until about eight o’clock in the morning, I feel a lot more refreshed than if I was to go to bed, keep getting up, sitting up, going out of my room and going doing this.”  “you’re going to disturb other people in the house”  “Your body needs that rest even though you’re not sleeping your body needs that rest.”  “You start finding something to do then, don’t you, and then get preoccupied with what you’re doing and…”  “Then I don’t get any sleep.” (personal experience) | n/a | 8 (31%) |
|  | Very many participants with personal experience separately raised this from direct personal experience, it was brought up in 4 out of 5 of the focus group discussions. No-one replied in these discussions describing a good experience when attempting to use the 15 minute rule. Professionals emphasised that stimulus control still restricts sleep and requires support and monitoring, but it sounded like these participants had been attempting to follow this based on one off advice. | | |

| Recommendation or issue raised | Data excerpts | professionals  n= (%) | personal exp.  n= (%) |
| --- | --- | --- | --- |
| Address activities to do if you awaken in the night | “not using screens, not eating when you get up” (sleep & MH researcher)  “move to quiet room with quiet activity until sleepy.” (sleep & MH researcher)  “so, if you’re thinking once you got up good let me read a technical journal so that I get sleepy, I go to my patient ‘ah ha! there’s sleep effort again!’ […] so the first rule is ‘get out of bed and do something fun’” (CBT-i practitioner)  “So I think having a lot of activity and things they enjoy doing that are really set up for them in that environment without a lot of thought involved.” (sleep OT CBTi practitioner) | 9 (16%) | 0 (0%) |
|  | Views differed regarding what types of activities are appropriate to do if awakening in the night and the best way to choose these, although participants agreed this should be addressed (see graph below). | | |
| Provide education on awakenings being normal | “What is normal sleep - many people I’ve assessed actually sleep fairly well but don’t realise it’s normal to wake several times per night and go straight back to sleep again” (psychiatrist) | 3 (5%) | 0 (0%) |

**Final stage, views from participants with personal experience:**

(Stimulus Control was explained in terms of:

Avoid doing other non-sleep activities in bed (e.g. reading,

phone, TV)

Reduces association of bed with wakefulness

Increases association of bed with falling asleep

Bed then acts as a ‘cue’ to the brain & body to fall asleep

• If not asleep after a set period (often 15 minutes) get up again

• Get into bed only when feeling sleepy

• If awakening in the night, get out of bed and do something until feeling sleepy again (explore and find suitable activities))

## Morning routine

| Recommendation or issue raised | Data excerpts | professionals  n= (%) | personal exp.  n= (%) |
| --- | --- | --- | --- |
| Address type of activities | “Activities that facilitate waking up and boosting energy. Ideally including light exposure.” (sleep & MH clinician researcher)  “And get out of the bedroom as soon as they have risen from bed.” (sleep specialist psychiatrist)  “…also with motivation and just knowing they have that consistent role of things they do, and can go into more autopilot in the morning” (sleep OT CBTi practitioner)  “a planned, pleasurable, meaningful activity” (MH OT) | 17 (30%) | n/a |
|  | The types of activities suggested were quite consistent and included energising activities, ideally going out of the house, or having light exposure, incorporating social contact or concrete plans early in the day if possible. Crucially, getting out of bed. Some discussed having a set morning routine of the same activities, and preparing for the morning, to make the morning easier once it comes. | | |
| Use of alarms | “Assisting clients with setting up an alarm on their phone etc if they do not know how” (sleep & MH clinician researcher)  “…I say to [my friend], can you ring me and make sure, you know, if I meet her in the morning” (personal experience)  “Respondent A: It would be okay for it to be in the room, but, like, far away, so I can’t press the button so that I can go back to sleep.  Facilitator: Okay. So that thing about alarms on the other side of the room, you didn’t think that was a bit much?  Repondent B: No, that’s a good idea.  Respondent A: Good idea.  Respondent C: Yeah” (personal experience) | 20 (36%) | 10 (38%) |
| Dawn simulator alarms | “Dawn simulation with light and sound will support the circadian system and sleep structure, including the microbiome” (MH & circadian rhythm researcher)  “I’m not worried they are harmful, more the unnecessary cost. I’m not convinced they are so very effective” (sleep specialist psychiatrist)  “It might set you into a routine so you don’t need it by the end of it, so you might just wake up on your own…” (personal experience)  “So, it’s like a brightness, isn’t it, when you first wake up in the morning, instead of waking up with your blinds open and it’s raining, you get a bright light and it’ll make you feel better.” (personal experience) | 6 (11%) | 9 (35%) |
| Education on sleep inertia | “Pts needs to understand that first 10-30mins may involve sleep inertia and as such should not use this fatigue feeling to gauge how well they slept.” (CBTi practitioner) | 4 (7%) | 0 (0%) |
| Experience of struggle with waking | “…it wouldn’t always wake me because I would be in such a deep sleep that it wouldn’t wake me.” (personal experience)  “Because I struggle to wake up. Like I struggle to get to sleep and then when I’m supposed to wake up I don’t wake up, not even for alarms and stuff.” (personal experience) | n/a | 8 (31%) |

**Final stage, views from participants with personal experience:**

## Evening routine

| Recommendation or issue raised | Data excerpts | professionals  n= (%) | personal exp.  n= (%) |
| --- | --- | --- | --- |
| Evening wind down activities, lower stimulus | “A buffer zone 30-60 minutes prior to bedtime to decrease arousal” (sleep OT CBT-i practitioner)  “having a wind down - similar every evening, calming relaxing routines” (sleep OT)  “…some people find structured relaxation stressful. maybe they could be in a passive activity rather than relaxation” (MH OT)  “It’s a very passive activity isn’t it [formal] relaxation whereas if you do something like having a bath listening to music, creating scent in your room, doing the knot tying, or whatever you want to do, that is more occupational of an intervention maybe” (MH OT)  “Sometimes I get to sleep by watching YouTube on my phone, with my eyes straining on the small screen, I actually get some sleep eventually.” (personal experience)  “I used to listen to music to help sleep; that worked for about a month or so, but then it just stopped working and it didn’t work any more.” (personal experience) | 27 (48%) | 13 (26%) |
| Preparation for bed before wind down | “Brush teeth and get ready for bed before wind down so that when bedtime comes pt can be ready to go straight to bed” (CBT-i practitioner)  “otherwise you go into the bathroom and is a really bright light” (sleep & MH researcher) | 3 (5%) | 0 (0%) |
| Prepare for the next day - if relevant | “Getting a chance to work out a plan for the next day” (sleep OT CBT-i practitioner)  “the value of this will probably vary between individuals. For some people, thinking about the following day may increase arousal levels” (sleep OT)  “…not everyone finds this an important thing to do. For those who would benefit and want to do it, it is very important” (sleep OT) | 6 (11%) | 0 (0%) |
| Support to find suitable activities | “…try to make it a quality time for them, especially if staying up much late than usual, don’t want it to feel like a punishment, it’s got to feel like a really nice time. I throw out lots of ideas but leave it to them to decide.” (sleep OT CBT-I practitioner)  “Provision of materials that they could try out for winding down - e.g. art and craft materials, puzzles, books, comics, podcasts, […] may usually be unable to afford these things or lack motivation to look for them on their own at first.” (sleep & MH clinician researcher) | 7 (12%) | 0 (0%) |
| Get ready for bed alarm | “a cue to start bedtime routine” (circadian rhythm researcher)  “I set my alarm to say that I need to go to bed at 10am, what I got into that routine I did sleep for longer than 2 hours, its hard to keep the routine.” (personal experience) | 2 (4%) | 1 (4%) |

**Final stage, views from participants with personal experience:**

## Daytime activity

| Recommendation or issue raised | Data excerpts | professionals  n= (%) | personal exp.  n= (%) |
| --- | --- | --- | --- |
| Increasing amount of activity | “many patients v lonely, and sleep because they are bored /nothing to do- good to encourage social activities” (sleep & MH researcher)  “Fill the day with interests and exercise.” (personal experience) | 28 (50%) | 16 (62%) |
| Address activity type | “Meaningful activities, relaxation activities, mix of ADLs, time outside, time doing physical activity, social contact” (sleep OT) | 52 (93%) | 18 (69%) |
| Address activity timing | “Level of activity and pattern of activity - looking at the time of day that activity occurs - impact that this has on light exposure - impact that activity can have on clock - is it periodic - will it impact ability to fall asleep” (circadian rhythm researcher)  “And having something to work with to highlight which ones are daytime and nighttime activities.” (personal experience) | 20 (36%) | 8 (31%) |
| Scheduling activities | “scheduling of activities, especially with commitments to others (e.g. meet a friend for coffee each morning)really helps” (CBT-i practitioner)  “Use of a wall calendar to prompt them to remember their plan for that day to encourage them to get going purposefully.” (MH OT) | 23 (41%) | 17 (65%) |
| Routines and habit formation | “Regularity of routines (including meal timing, walks, social interaction etc.) to strengthen circadian rhythm” (MH & sleep researcher) | 16 (28%) | 3 (12%) |
| Meaning, satisfaction and enjoyment | “Meaningful activities - if activity is replacing time in bed trying to sleep then those activities should be valued and useful in working toward a valued "future self"” (sleep OT CBTi practitioner)  “Satisfaction, isn’t it. If you’re doing things for yourself all the time it’s not; whereas if you’re doing something that’s worthwhile and contributing – I think most people want to contribute.” (personal experience) | 21 (38%) | 7 (27%) |
| Support to find and plan activities | “Very explicitly planning activities, checking in on what was helpful and what wasn't done, creatively brainstorming things to increase activity and light exposure” (sleep & MH researcher)  “[If its not scheduled it will] …give people a chance to think and talk themselves out of doing it. […] If it’s more vague it’s not definitely going to happen, is it?” (personal experience) | 9 (16%) | 5 (19%) |

**Final stage, views from participants with personal experience:**

Explained in terms of:

Physical activity -more tired - sleep better Mentally active tasks -feel alert - less naps

Outdoor activities improve light exposure Moving stressful activities away from bedtime

Regular meal times to structure the day (& not eating too late)

Might involve: • choosing activities • setting graded goals • Scheduling • involving friends & family

(images of paper and electronic diaries)

## Addressing medications

| Recommendation or issue raised | Data excerpts | professionals  n= (%) | personal exp.  n= (%) |
| --- | --- | --- | --- |
| Consider side effects | “Antipsychotics may improve and extend sleep appropriately. There are some without significant insomnia for whom APs may cause hypersomnia” (sleep specialist psychiatrist)  “some people seem to be sedated where others aren't at all.” (MH OT)  “Facilitator: Tablets? In what way?  Respondent A: They can make you either too tired or to hyper.  Respondent B: They can paralyse your body and not turn off your mind.  Respondent C: Or they can sedate you so you’re not as active during the day.  Respondent A: So you’re just a cabbage basically, aren’t you, for 90 per cent of the week.” (personal experience) | 8 (14%) | 8 (31%) |
| Addressing timing of prescribed medications | “correct timing eg mane or nocte” (psychiatrist)  “understand side effects of medication, timing of intake, and why they are prescribed at certain times of day and not others, promote adherence to routine” (sleep OT)  “Where medication is at odds with the sleep window this is important, but I would always recommend doing this work in liaison with the prescriber” (sleep & MH clinician researcher)  “Consider medication timing as part of evening routine.” (sleep specialist psychiatrist)  “what time do you take them, do you get a hangover in the mornings?” (psychiatrist)  “Well, I know there are certain medications that you’re meant to be taking earlier rather than later because they will help you sleep, but sleep too much. So, if you took it say at ten o’clock at night you might be still in bed at ten o’clock in the morning. So, it’s advisable to take it say at six o’clock in the evening. Things like that that could really make a difference.” (personal experience) | 19 (34%) | 3 (12%) |
| MDT approach | See below: 7. Implementation considerations MDT approach within intervention, MDT approach to medication. | | |


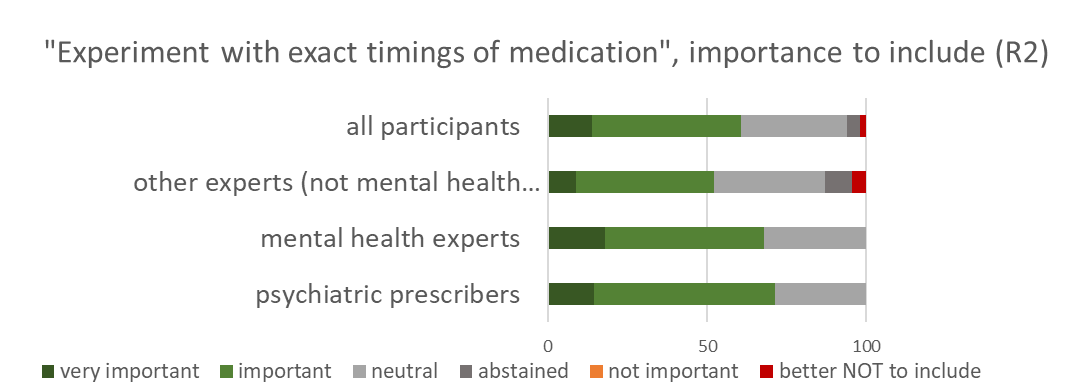


Round 3:

## Addressing food and drink

| Recommendation or issue raised | Data excerpts | professionals  n= (%) | personal exp.  n= (%) |
| --- | --- | --- | --- |
| Consider food and drink timing | “timing of meals to help add structure and pattern to the day which may be chaotic because of the current difficulties with sleep.” (MH OT) | 49 (88%) | 4 (15%) |
| Address avoiding late eating | “…not too much food too close to bedtime.” (sleep OT CBTi practitioner)  “A light snack 1-1.5hrs before bed is great marker and prevent hunger in middle of night. Pt should avoid heavy foods close to bedtime” (CBTi practitioner) | 25 (45%) | 1 (4%) |
| Address skills and/or routines around meals | “Also elements of how to integrate this into a routine of ensuring the appropriate resources are available, and the person has the skills, to prepare meals on time and budget (i.e. not realising there is no food and going for a late takeaway on a regular basis)” (MH OT)  “…increasing awareness and reinforcing planning which is consistent with having adequate time for sleep […] includes have decent food in the fridge and time set aside” (sleep OT) | 15 (27%) | 0 (0%) |
| Night eating | “If someone isn't sleeping well it is not unusual for them to be hungry in the middle of the night. If they must eat, then high tryptophan lowish glycaemic index food may be helpful. Half a banana for example.” (sleep OT CBTi practitioner)  “[Night eating] has not been the key part of the clinical formulation for any client that I've met with insomnia and psychosis.” (sleep & MH clinician researcher)  “I don’t… encounter it because I don’t do it myself and a keep forgetting to ask people about it, so that’s a shortcoming in me, I’m sorry I should ask more.” (sleep specialist psychiatrist)  “night eating needs to be addressed in the context of culture. some cultures have emphasis on night meals vs day meals and also seasons for example during muslim fasting month of Ramadan night eating becomes a social norm.” (sleep OT)  “What I find is…I have broken sleep. I go to bed about 11 o’clock, every two hours, I mean, it’s like clockwork, what I find is if I go down and have a half a banana or a banana, I eat something, I comfort eat in the middle of the night [inaudible] send me to sleep…and now I have a weight problem” (personal experience) | 18 (31%) | 1 (4%) |
|  | Participants varied between feeling this was very common to being slightly surprised when it was raised (Round 2 onwards). One participant admitted feeling it is probably common but not having been asking about it. | | |
| Consider food and drink content | “Avoiding heavy / spicy meals late at night” (sleep OT)  “a nice breakfast as something to look forward to on getting out of bed can be successful.” (sleep & MH researcher)  “at the same time, OT should not be the one main one assessing a person's diet for nutritional content and calories” (sleep OT) | 28 (50%) | 6 (23%) |

## Addressing substance use

| Recommendation or issue raised | Data excerpts | professionals  n= (%) | personal exp.  n= (%) |
| --- | --- | --- | --- |
| Substance use | “[work on reducing] use of substances (especially cannabis and alcohol) to aid sleep.” (sleep specialist psychiatrist)  “[Circumstances to exclude for drug use] An example of a hard core amphetamine use who would stay awake for 4 days then just crash […] so if drugs are one of the main determinants of your sleep then that would have to change first” (MH OT)  “I think cannabis would be alright if you’re given the right dose. That’s more natural than any of this crap they give you.” (personal experience) | 18 (32%) | 5 (19%) |
| Alcohol | “Substances especially alcohol very important. Alcohol disrupts sleep physiologically (the need to urinate) and even on slight withdrawal (few units) heightens anxiety which when awoken in the night or morning can prevent a return to sleep” (psychiatrist)  “…reduce or eliminate alcohol entirely when in active treatment because drinking at night increases sleepiness and is counterproductive to maintaining a later bedtime” (CBTi practitioner) | 12 (21%) | 2 (8%) |
| Caffeine | “Priority in terms of food/drink content is caffeine, especially if the person may not realise caffeine content of certain food/drinks.” (MH & sleep researcher)  “… adjust this to the patient as some are rapid metabolisers” (psychiatrist)  “…cannot be one rule for all.” (MH OT)  “Caffeine has a 6-8 hour half life. It should therefore be stopped by 2pm for most people” (sleep OT CBTi practitioner)  “…after e.g. 6 p.m” (MH & circadian rhythm researcher) | 24 (43%) | 5 (19%) |
| Nicotine | “smoking, is best avoided as it can act as a reward or reinforcement for waking.” (sleep OT CBTi practitioner)  “no heavy meals/alcohol/smoking late at night” (MH OT)  “very hard to reduce smoking” (MH OT)  “maybe have cigarette and alcohol intake [on the initial assessment] but explain that this isn’t to police what they drink and smoke” (MH OT)  “Respondent A: I’ve stopped smoking an hour before I go bed.  Respondent B: That’s something I couldn’t do.” (personal experience)  “I tell you what I do do in bed when I can't fall asleep, smoke. […]Which that keeps me awake even longer […] if I’ve done that half the night then I’m shattered.” (personal experience) | 9 (16%) | 4 (15%) |

## Light Exposure

| Recommendation or issue raised | | Data excerpts | professionals  n= (%) | personal exp.  n= (%) |
| --- | --- | --- | --- | --- |
| Modifying light exposure | | | 49 (88%) | 21 (81%) |
| Timing of modifications to light | Morning light exposure | “Getting morning exposure to daylight is the key to entrainment” (sleep OT CBTi practitioner) | 18 (32%) | 1 (4%) |
|  | Daytime light exposure | “Daytime light exposure because of improving sleep quality, more deep sleep, less fragmentation, less sleepy at waking up” (circadian rhythm researcher)  “I think its better to be out. I tend to start sleeping if its dark” (personal experience) | 11 (20%) | 10 (38%) |
|  | Increasing evening light | “for earlier sleep bright light early morning, for later bright light in the evening - even better if linked with exercise” (sleep OT CBTi practitioner) | 2 (4%) | 0 (0%) |
|  | Reducing evening light exposure | “7pm it is fine to have whatever light […] into evening and it is before you are going to sleep, this is when it is really important as it allows the melatonin” (MH & circadian rhythm researcher)  “…a lot of effort for minimal benefit. The evidence that evening light is detrimental is equivocal at best. I would just focus on morning light...” (sleep specialist psychiatrist)  “Respondent A: …if you keep looking at your phone light, it’s been scientifically proven, that light on your phone, it’s called blue something, I can’t remember the full name of it but it keeps you awake.  Facilitator: What do you all think about that? Do you all have your phones in bed with you or a computer in bed?  Respondent A: I’ve got laptops everyone does.  Respondent B: I leave my phone switched on but I don’t use the phone, but if I did use my phone before I went to bed, I could still sleep.” (personal experience) | 29 (52%) | 15 (57%) |
|  | Reducing light at night | “Quiet, dark, cool bedroom.” (CBTi practitioner)  “black out curtains” (MH OT)  “Have safe alternative for night trip to bathroom etc rather than full lights on.” (sleep OT)  “I’ve got eye patches at home […] I’ve got to be in complete dark.” (personal experience)  “That ‘cut out all the light’, I can't sleep like that, I have got to have the lobby light on […] never been able to sleep without the light on.” (personal experience) | 30 (54%) | 11 (42%) |

| Recommendation or issue raised | | Data excerpts | professionals  n= (%) | personal exp.  n= (%) |
| --- | --- | --- | --- | --- |
| Method to modify light | Light box | “during winter, could use SAD lamp for 30 minutes after waking if it is still dark” (sleep & MH researcher)  “I’m not opposed to light boxes, its harder to get them, whose paying for them etc.” (CBTi practitioner) | 15 (27%) | 15 (57%) |
|  | Light visor | “use of light mask?” (sleep OT CBTi practitioner) | 1 (2%) | 0 (0)%) |
|  | Blue-blockers / amber glasses | “high effect sizes in the few studies undertaken in dark therapy […] definitely many people with sleep problems report very dramatic changes in sleep” (MH & sleep clinician researcher)  “I prefer to lower intensity more than filtering out specific colours. So, I am more in favor of lowering environmental light” (circadian rhythm researcher)  “…number of pts willing to try it [amber glasses] was too few” (sleep specialist psychiatrist)  “Respondent A: Yeah, I think they’d be all right.  Respondent B: Just for a couple of hours at nighttime…  Facilitator: Yeah. You wouldn’t be worried about what they look like particularly?  Respondent C: No, because you’d be in the house anyway.  Respondent D: I don’t like wearing sunglasses or anything. […] Just irritate me.  Facilitator: Mmm. Oh, like, what it feels like on your face?  Respondent D: Mmm.” (personal experience) | 6 (11%) | 4 (15%) |
|  | Modifying light in the home & bedroom | “Indoor light is often very low and not bright enough, irrespective of window covering. Only if you sit next to the window or with very large windows [so] I am more in favour of using light boxes than addressing window coverings.” (circadian rhythm researcher)  “[should address] Light environment - lighting, windows (access to natural light), where spend the majority of time, electronic devices” (circadian rhythm researcher)  “Respondent A: I suppose that could work, yeah; because I normally sit in the dark, all, like, in a darkened room; so, I could try opening the curtains more, try and lighten the place up a bit. […] Respondent B: I do the same, keep the blinds shut so it just feels like the outside isn’t there.” (personal experience) | 28 (50%) | 6 (23%) |
|  | Using outdoor light / natural light | “lack of exposure to daylight seems to be a significant issue for many people with psychotic disorders” (psychiatrist)  “Ideally, the person should just get outside. In cases where that is difficult use of a light box is a possibility” (sleep OT CBTi practitioner) | 29 (52%) | 0 (0%) |

| Recommendation or issue raised | **Data excerpts** | professionals  **n= (%)** | personal exp.  **n= (%)** |
| --- | --- | --- | --- |
| Season is important | “UK is relatively far north and has significant seasonal differences in the number of hours of daylight per day” (sleep OT CBTi practitioner)  “anchor point in the morning […] will probably change with season. if [sleep duration] is long, is it also long in the summer?” (circadian rhythm & MH researcher)  “I go into hibernation when it's winter and want to have summer/spring for light mornings as I feel happier.” (personal experience) | 12 (21%) | 4 (15%) |
| Embedding light in activity / occupation | “More activity outdoors to reduce social isolation” (sleep & MH clinician researcher)  “Also, if people are getting more daylight exposure, they are likely to do so by acting in ways that would be beneficial for other reasons (eg. benefits of leaving the house or light exercise)” (psychiatrist)  “[dog walking] Routine and daylight so you get multiple things.” (personal experience) | 8 (14%) | 1 (4%) |
| Education regarding light, circadian rhythm and mood | “I explain it to people in terms of evolution, that we are day-active animals, humans are not nocturnal, so seeing light makes us want to be alert and awake.” (sleep & MH clinician researcher)  “empower them to take charge of light exposure” (circadian rhythm & MH researcher)  “actually the thing is most people respond best to is not the circadian rhythm or the vit D, its that daylight is as effective as antidepressants […] it’s a better incentive for ppl.” (sleep OT CBTi practitioner) | 27 (48%) | 0 (0%) |
| Low expectation of efficacy regarding light | “Don't think I'm affected by circadian rhythm very much.” (personal experience)  “I’m going to be honest with you, what I’ve noticed is all this stuff with light and all that, yes, it does affect other people differently but if you’ve got insomnia because of mental health problems, whether it’s light or dark […] it doesn’t matter what colour it is outside your eyes, you’re not going to sleep.” (personal experience) | 0 (0%) | 10 (38%) |
| Acute alerting effects of light | “not bright light right up till bed […]so can wind down and feel like its evening” (sleep OT)  “The basic research is very convincing that light is activating and sleep-disturbing […] It is also true that the clinical studies are few and with low *n* for the time being” (MH & sleep clinician researcher) | 4 (7%) | 1 (4%) |

**Final stage, views from participants with personal experience:**

This is the component people knew least about. Whilst some were enthusiastic about light modification others felt it would not work. Awareness of the non-visual effects of light was poor. Unsurprisingly the avoiding screens at night was not popular.

## Environmental assessment and intervention

| Recommendation or issue raised | | Data excerpts | professionals  n= (%) | personal exp.  n= (%) |
| --- | --- | --- | --- | --- |
| Home environment | Home environment | | 54 (96%) | 18 (65%) |
|  | Bed or sleeping surface | “whether they have a comfortable bed/ pillows/ covers/ curtains” (sleep & MH researcher)  “do they have a bed and do they use it” (psychiatrist) | 23 (41%) | 0 (0%) |
|  | Bedroom / bed not for non-sleep activities | “Creating/keeping a dedicated sleeping space” (sleep OT)  “Is bed in same room. Can you set up separate zone etc.” (sleep & MH clinician researcher)  “That’s what beds are for anyway, just to sleep.” (personal experience)  “ [I know about] not going on phones in bed, or watching TV where you lay in bed, and stuff like that. […] I do it all the time.” (personal experience) | 28 (50%) | 13 (50%) |
|  | Having other useable rooms | “many of our patients live in cramped conditions and have to multi-purpose their bedrooms” (MH OT)  “A person may have neglected all areas but bedroom” (MH OT) | 6 (11%) | 0 (0%) |
|  | Noise in the bedroom | “noisy neighbours” (sleep & MH researcher)  “white noise […]something that negates outside noise.” (sleep OT)  “ear plugs needed?” (sleep & MH clinician researcher)  “earplugs […] may also block smoke alarm signals […] ideally noise should be stopped at the source, tho' this is not always easy.” (sleep OT)  “Sleep spindles are believed to be protective of sleep disruption by noise. [People with schizophrenia] are thought to have fewer and may be more vulnerable. Therefore a quiet environment is particular critical” (sleep OT) | 34 (61%) | 0 (0%) |
|  | Temperature in the bedroom | “comfortable temp - cooler at night” (MH OT)  “bedroom too hot = difficult to sleep; but bed cosy = easier to sleep” (sleep OT) | 36 (64%) | 1 (4%) |

|  | Air quality | “airing room” (MH OT)  “mould - damp - allergens (blocked nose at night)” (sleep consultant)  “I imagine air quality might be the hardest of these variables to address with limited financial resources” (MH OT) | 7 (12%) | 0 (0%) |
| --- | --- | --- | --- | --- |
|  | Sensory factors | “colour of furnishings and walls / calm vs hectic” (sleep consultant)  “possibly also general clutter, safety, comfort, being a nice space to settle into for sleep.” (sleep OT)  “ask about how they feel about the environment, the pyjamas, the sensory qualities of the room” (sleep OT)  “Suspect this is a much less important factor to prioritise for most clients, but could be important for a minority.” (sleep researcher) | 29 (52%) | 1 (4%) |
|  | Pets in the bedroom | “Not pets in bed” (CBTi practitioner) | 9 (16%) | 0 (0%) |
|  | Home environment intervention | “Floor should be clear to prevent falls” (sleep OT)  “Function and tidiness to allow visitors” (sleep & MH researcher)  “options to change bed/environment to improve sleep pattern may be limited due to financial constraints” (sleep OT CBTi practitioner)  See also ‘3. The Assessment - Format and Manner of the assessment - Rapport’, rapport required for home assessment | 13 (23%) | 7 (27%) |
|  | Feeling safe in the home | Do they feel the environment is safe? (sleep specialist psychiatrist)  “Home security. Police advice. Reduce anxiety.” (MH OT) | 14 (25%) | 1 (4%) |
| Social environment & context | Social environment & context | | 44 (79%) | 17 (65%) |
|  | Social environment in the home | “consider the older person who lives in a child's house, and how they go to sleep early because they don't want to disturb the others. They're not sleepy at 8:00 pm but just want to get out of the way.” (sleep OT)  “Assess bed partner issues” (CBTi practitioner)  “…for example the person might live with several noisy siblings or family members and chose to stay up all night when it is quiet as a result” (MH OT) | 12 (21%) | 1 (4%) |
|  | Support from friends, family and carers | “support person to identify network of friends family who can support them to keep up new activities/do activities with” (MH OT)  “Would need help from family member / friend. But would give it a go with help.” (personal experience)  “Involving family may not be helpful for independent. Friends may not want to get involved.” (personal experience) | 16 (28%) | 11 (42%) |
|  | Social commitments | “scheduling of activities, especially with commitments to others (e.g. meet a friend for coffee each morning) really helps.” (CBTi practitioner)  “Initial interview will establish shift patterns, school hols, split parenting routines and other factors that may vary from week to week” (MH OT) | 13 (23%) | 5 (19%) |
|  | Peer support | “Group sessions, led by appropriate staff, may provide some valuable peer support for patients and address this” (sleep OT CBTi therapist)  “Group treatment can be highly effective to make changes” (sleep OT)  “Groups exclude people with poor transport and chaotic people” (MH OT) | 18 (32%) | 0 (0%) |
|  | Loneliness | “many patients v lonely, and sleep because they are bored /nothing to do- good to encourage social activities” (MH & sleep researcher) | 4 (7%) | 0 (0%) |
|  | Cultural factors | “Incorporating cultural needs - such as Ramadan, Kosher meal restrictions” (MH OT)  “Western culture may over-value an uninterrupted night of sleep, as opposed to two phases of sleep at night” (sleep OT) | 6 (11%) | 1 (4%) |

## Relaxation and / or mindfulness

| Recommendation or issue raised | Data excerpts | professionals  n= (%) | personal exp.  n= (%) |
| --- | --- | --- | --- |
| Mindfulness and relaxation were often brought up together, sometimes interchangeably by participants. This is not to say that all that these participants all thought they were equivalent, but some saw them as interchangeable within a sleep intervention. | | 46 (82%) | 13 (50%) |
| Relaxation techniques | “Individual responses to each of these relaxation strategies can vary significantly” (sleep OT)  “offer a selection for person to trial” (MH OT)  “a body-based relaxation technique (yoga, progressive muscle relaxation) may be preferred over a purely "mindful" approach.” (sleep OT CBTi practitioner)  “I would see these things as an additional adjunctives rather than prioritised within the intervention.” (sleep & MH researcher)  “practice this during the day for several weeks, until they have built up their skill level, before introducing into a bedtime routine” (sleep OT CBTi practitioner)  “definitely in the daytime, I wouldn’t use it as a strategy to help you fall asleep.” (sleep OT CBTi practitioner)  “I do not favour using any kind of relaxation routine (such as PMR) in bed - especially if it involves a recording.” (sleep OT CBTi practitioner)  “…when you’re in that panic, when you’re in that state of, like, anxiety […] struggling to breathe, just take a minute to just sit down and feel what’s around you and listen to different sounds, and just focus on that.” (personal experience)  “I find that it’s just another thing that I’m thinking about, and that keeps me well awake.” (personal experience) | 45 (80%) | 13 (50%) |
| Breathing techniques | “concentrate on breathing when in bed” (circadian rhythm & MH researcher)  “Perhaps try breathing exercise as the main [approach]” (sleep & MH clinician researcher) | 21 (38%) | 0 (0%) |
| Mindfulness meditation | “Relaxation techniques (muscle relaxation, guided visualisation, mindfulness, breathing etc.)” (sleep researcher)  “for me mindfulness meditation you cant really separate that from breathing techniques. […] Mindfulness again its not necessarily about producing sleep is it…” (MH OT)  “Mindfulness is not about relaxation, that’s a misunderstanding, anything you get out of mindfulness that moves you in the direction you thought you might want to go in is a side effect, the main effect is being present, and getting good at feeling what you’re feeling” (CBTi practitioner)  “Oh, yes, it’s really good. It helps me relax but it’s not supposed to send you to sleep but it always sends me to sleep.” (personal experience) | 17 (30%) | 7 (27%) |

## Thermoregulation

| Recommendation or issue raised | Data excerpts | professionals  n= (%) | personal exp.  n= (%) |
| --- | --- | --- | --- |
| Thermoregulation | “lower the bedroom temperature, open the window wide for 20min and allow fresh air to come in before going into bed” (circadian rhythm & MH researcher)  “With regards to bedding, it could also be considered in the sense of thermoregulation - e.g. using thinner sheets if they get hot easily in bed.” (sleep & MH clinician researcher)  “The effects of cold feet are underestimated and very simple to address. However it is important to warm the feet PRIOR to going to bed.” (circadian rhythm researcher)  “Cold feet prevent vasodilation and impair drop in core body temperature which inhibits or at minimum delays sleep. This is a very well evidenced effect.” (sleep OT)  “The evidence for wearing warm footwear is not strong” (sleep specialist psychiatrist)  “Clearly if this is an issue for some clients it should be addressed, but for many I suspect it could take away time from other aspects of the intervention” (sleep & MH clinician researcher)  “timing of shower or bath (makes a difference to sleep onset)  […]not 'hot' bath or shower; instead, say warm (avoid really hot or really cold)” (sleep OT)  “Less convinced by evidence on hot bath/shower, but could be suggested as part of establishing an evening routine if the client felt a bath/shower worked well for them.” (sleep researcher)  “It could be mentioned that having an evening soak in the bath may be helpful in inducing sleep” (personal experience) | 34 (64%) | 7 (27%) |

Round 3:

## Addressing sensory factors

For qualitative content see above in ‘sensory factors’, under Environmental assessment and intervention. Sensory factors were mostly related to the bedroom, but also to nightclothes.

Round 3:

## Cognitive or psychological approaches

| Recommendation or issue raised | Data excerpts | professionals  n= (%) | personal exp.  n= (%) |
| --- | --- | --- | --- |
| Cognitive or psychological approaches | “factors over which there is no control. It may be necessary to think about how to manage thoughts about such things (there is no point getting angry about trains on the line at the end of the garden which was there before you moved in the house)” (sleep OT CBTi practitioner)  “Book to write worries in if needed” (MH OT)  “Putting the day to rest, eg. wind down, worry management” (sleep & MH clinician researcher)  “Thought records, downward arrow techniques, reviewing evidence, worry time, mindfulness, testing of safety behaviors” (CBTi practitioner)  “From my standpoint, I might touch on it, but if that’s someone’s main problem I might refer to a psychologist or a MH clinician, its not that I don’t address it but if that’s their main thing - and the CBTi I’ve learned is not so much focused on the cognitive components, its more SR, routines” (sleep OT)  “the only reason to use cognitive technique is to get the person on board to do what they need to do” (CBTi practitioner)  “I honestly think every patient should receive some cognitive input, at minimum around helping them to drop the struggle, which perpetuates insomnia and around activating the PNS.” (sleep OT CBTi practitioner)  “I feel using cognitive techniques in addressing dysfunctional sleep beliefs is within OT scope” (sleep OT CBTi practitioner)  “and the psychologist said to me instead of worrying about everything all day, choose a time, say it’s like five o'clock, and for that hour you can think about it through that hour, and then the rest of the day you forget about it. […] But it’s not that easy.” (personal experience)  “I’m not sure about that because is it, kind of, like counselling? […] If it’s going to mean I have to talk about things that might upset me then that’s not going to be a good thing…” (personal experience) | 35 (62%) | 5 (19%) |
| Psychological approaches better dealt with by psychological therapist | “if you are going too far down the CBT route you should probably be using psychological therapists.” (MH OT)  “If more cognitive approaches are needed, refer to a psychologist. You do not need to have all expertise in one hand.” (circadian rhythm & MH researcher) |  |  |


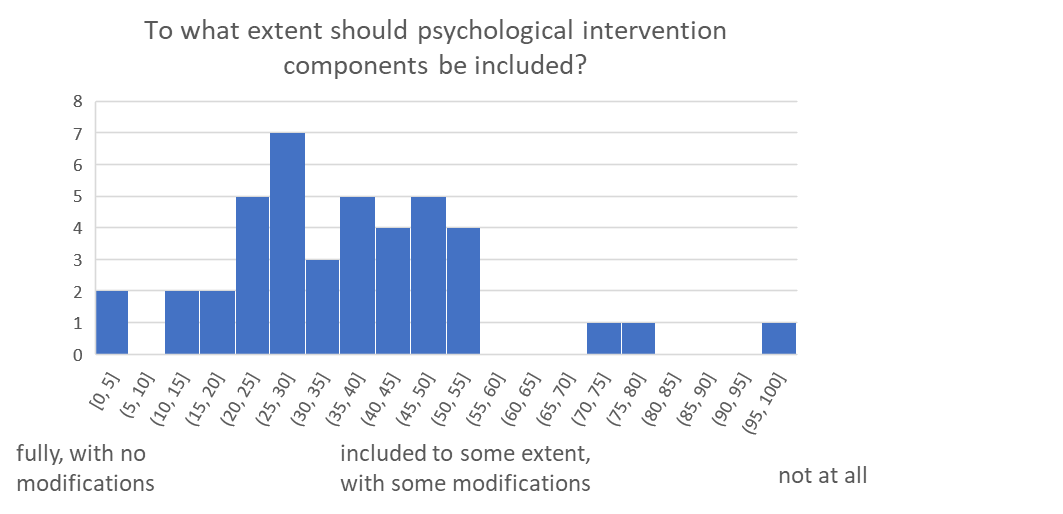


It is acknowledged in hindsight that there may be a social desirability bias in responses to this question as it was posed partly in relation to points made about the skills and professional remit of OTs. There may have been a bias toward rating higher toward inclusion in order to reflect positively on the skills of OTs, a group of which the researcher and many participants in the study are members, rather than ratings reflecting the ideal format for inclusion within this particular intervention.

**Intervention components and intervention descriptions - Final stage, views from participants with personal experience:**

# Personalisation

| Recommendation or issue raised | Data excerpts | professionals  n= (%) | personal exp.  n= (%) |
| --- | --- | --- | --- |
| The goals of the intervention should be individually determined | “ask them to write down their key concerns about their sleep” (sleep & MH researcher)  “what is the one thing you want to change most of all, and why…” (OT CBTi therapist)  “For me naps depend on whether patient wants to be a napper when Tx is done” (CBTi therapist)  “…and why should you make them mainstream” (circadian rhythm & MH researcher)  “flexibility for individual patients' interests and preferences” (psychiatrist)  “what (if anything) they feel they can/wish to change.” (MH OT)  “I think it’s really difficult to say, this is the way everybody will… Because people have different sleeping patterns, they have different interests” (personal experience)  “go through everyone’s list and say which of these is most important to you? Right, we’ll do the intervention on that. A tailored personalised…” (personal experience) | 22 (%) | 3 (%) |
|  | Although some advocated a fairly standardised protocol, there was always some degree of flexibility in aims advocated. Flexibility ranged from fairly limited: such as personalising target sleep onset and offset times, to much more wide ranging flexibility in aims. CBTi therapists were less likely to be flexible about avoiding or banning naps, other participants views varied more regarding sleep schedule and naps. | | |
| The methods of intervention should be personalised | “Some individuals may be more sensitive to light than others” (OT CBTi therapist)  “what is done first will depend to a great extent on the person.” (OT CBTi therapist)  “Individual responses to each of these relaxation strategies can vary significantly” (OT sleep researcher)  “But if you have somebody with a severe delay […] I never [sleep] restrict those people” (CBTi therapist)  “some people may be able to maintain more irregularity and maintain progress towards good sleep than others.” (MH OT)  “Chair: You don’t think it [relaxation] has to be like, it should be used with everyone?  Respondent A: No.  Respondent B: No. Everyone’s different.” (personal experience)  “We’re all unique and we all do it different ways.” (carer / significant other)  “There’s never going to be an intervention that’s right for everybody.” (carer / significant other) | 38 (68%) | 9 (35%) |
|  | Sometimes personalisation was advocated around sleep schedule. Occasionally personalisation was described around light. Very commonly personalisation was advocated around relaxation, mindfulness, and sensory approaches. Reasons for personalising included around individual client preferences, and around different sleep problem phenotypes. | | |
| Limits to personalisation | “while individuals will vary, it is not practical formally to offer a range of techniques when there are so many other things to do” (OT CBTi clinician)  “it is also an issue of whether one is sacrificing time on another aspect of the intervention by including it” (sleep & MH clinical psychologist)  “standard forms like ISI, GAD and PHQ, I think doing those is really helpful to start a conversation if you are a bit unsure” (sleep OT clinician)  “I would have thought the level of structure required may depend on the clinician's expertise and confidence, therefore, needs some flexibility across different levels of expertise in how the assessment is performed. That said, I would have thought it needs to be fairly structured across the board to be consistent in gathering information on what are determined the important areas” (MH OT) | 10 (18%) | 0 (0%) |
|  | Increasing personalisation although desirable in principle, was acknowledged as potentially increasing the length of the intervention or the assessment, as well as increasing training and experience demands on the therapist. This could impact on the confidence of the therapist in delivery, which may impact outcomes (see code below). | | |
| Other areas in which personalisation was discussed | See also content relating to personalisation in the following areas and sections:  Accommodating differences in: occupations and interests, social environment and context, cultural factors, home environment  Considering the impact of differences in: light sensitivity, caffeine sensitivity, and medication side effects, individual responses to relaxation, sensory factors and thermoregulatory input  Choices in format for activity and sleep diary discussed. | | |

# Format, structure and pragmatic considerations

## Personalisation and complexity vs simplicity to deliver

| Recommendation or issue raised | Data excerpts | professionals  n= (%) | personal exp.  n= (%) |
| --- | --- | --- | --- |
| Personalisation | See section 1. Personalisation, final sub-topic “limits to personalisation”. A tension was identified between the need to personalise the intervention, and to make it simple enough to be delivered confidently and not confusing to therapist or client (see also below 6. Therapeutic Approach and therapist factors, which discusses confidence, knowledge and skills required to deliver the therapy.) | | |
| Keep it simple | “rules of thumb are important in a program and I like the idea of general guidelines since in the real world we have little time to 'customise' everything” (sleep OT)  “The protocol should fit onto an easily legible A4 format sheet, and only one!” (sleep specialist psychiatrist)  “There is a huge amount here, and potentially daunting for non-sleep-experts. Therefore keep it really simple, modular” (sleep specialist psychiatrist)  “I guess you have to limit it somehow, but sleep involves EVERYTHING. […] you can’t focus on everything really” (sleep OT)  “…what is the one thing you want to change most of all, and why is that most important thing for you to change now, because we probably wouldn’t look at trying to do the whole thing, it would just be change one thing at a time.” (sleep OT CBTi practitioner) | 22 (39%) | 0 (0%) |

## Format of intervention and assessment materials

See also section 2. The Assessment, for discussion of diary options to suit different individuals technological literacy and preferences, and use of technology in passive monitoring of sleep, activity and light.

| Recommendation or issue raised | Data excerpts | professionals  n= (%) | personal exp.  n= (%) |
| --- | --- | --- | --- |
| Format options & literacy | “Some patients have poor concentration [and / or literacy need to vary teaching approaches/information sources” (MH OT)  “Large print and check readability and understanding of written content” (sleep& MH clinician researcher) | 4 (7%) | 0 (0%) |
| Use of technology in delivery of the intervention | “a smartphone app for use alongside the intervention and to carry on the techniques after the intervention period ends?” (MH researcher)  “In terms of high tech or low tech, I think people should be given an option.” (sleep OT)  “Signposting to websites, apps, pod casts, books etc” (MH OT)  “ Facilitator: Some people said about like therapists could call people or text people…  Respondent A: Yeah.  Respondent B: Yeah  Facilitator: …at certain times.  Respondent B: I’d prefer the text at certain times, myself.  Facilitator: Yeah. If we had like an automated texting system that we could set them all in advance, would that be as good as a real therapist texting you?  Respondent B: Erm, I think…Yeah, I think it would, to be honest.  Respondent C: Yeah, I think it would. Yeah.” (personal experience) | 24 (43%) | 9 (35%) |

## Core vs optional components

| Recommendation or issue raised | Data excerpts | professionals  n= (%) | personal exp.  n= (%) |
| --- | --- | --- | --- |
| Core vs optional components | “Yes so for a lot of ppl if they can stick to SRT and stimulus control, I find they are sleeping through 85% of the time, for some there are sensitive people, or the temp in the room is high, caffeine at night, those things you are going to have to address. For the most part the core sleep behaviours take care of it.” (sleep OT CBTi practitioner)  “If the effects of modifying light environment causes deactivation instantly and alters sleep very much- this will make some other interventions less necessary […] that might still be necessary for some.” (circadian rhythm & MH clinician researcher)  “Everything needs to be covered, it is just a case of when” (MH OT)  “I feel the 'core' (apply to everyone, cover at the beginning and middle) and 'optional' (applies to some individuals, cover towards middle and end) makes sense […] makes it more manageable for the clinician, and also patient.” (sleep specialist psychiatrist) | 9 (16%) | 0 (0%) |

## Order of delivery

| Recommendation or issue raised | Data excerpts | professionals  n= (%) | personal exp.  n= (%) |
| --- | --- | --- | --- |
| Order of delivery | “Focus on establishing sleep schedule (supported by evening routine and light exposure) to anchor circadian rhythm and improve sleep efficiency first. Then add in other components as sleep efficiency and sleep timing stability are improving.” (sleep researcher)  “Although I would not think that relaxation is the solution for many people, it would make sense to start early on it as it takes time to learn.” (sleep OT)  “psychoeducation would usually be first, but then rest of it probably relies on what is going on for that person” (sleep & MH researcher  “it is all important, should almost all be delivered early on and repeated often!!” (sleep specialist psychiatrist)  “While I might tend toward a set order, I would also want to be listening first to what the patient experiences as the critical sleep issues..because developing a good therapeutic relationship will expand trust and motivation leading to treatment success.” (sleep OT)  “’The order will depend on the complaint and the baseline pattern […] open to changes according to what the client sees as manageable concurrently and what is a 'no-no' for the client.” (circadian rhythm & MH researcher)  “If all interventions are given at once, the learning for patients about the effects is confounded.” (circadian rhythm & MH clinician researcher) | 15 (27%) | 0 (0%) |


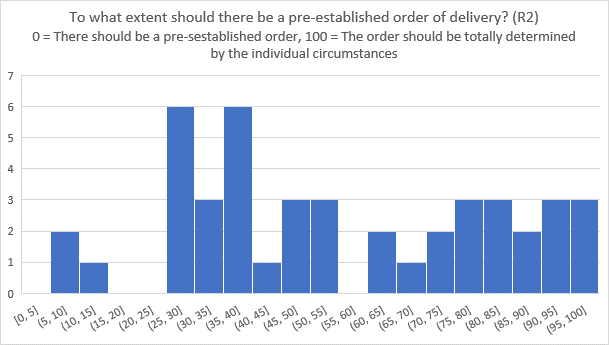


## Follow up and ending of therapy

| Recommendation or issue raised | Data excerpts | professionals  n= (%) | personal exp.  n= (%) |
| --- | --- | --- | --- |
| Maintenance plan | “Try to work out how they can remember the steps that are involved, because it’s really easy to go back to old habits. Whether you have it in a written form, or you could do a chat about what’s important and what their goals are and actually record something for them” (sleep OT).  “Having clear goals and behavioural plans to take forward, tools to self monitor improvements” (MH OT)  “Go through a relapse prevention form and write down all the things that worked for that person so they have it to refer back to. Teach them what you are doing and why so they take that with them and don't just do the intervention but once done know how to do it themselves” (sleep OT CBTi practitioner)  “Positive sleep maintenance plan (as opposed to relapse prevention plan)” (MH OT) | 23 (41%) | 0 (0%) |
|  | See also 7. Implementation considerations, MDT approach within the intervention, MDT involvement in maintenance. | | |
| Follow up / tapering of ending | “Maybe a follow up review 6 months or so after the intervention to review progress” (psychiatrist)  “support group, either online or in person” (sleep OT)  “Follow up visits. Mobile-phone app for long term recording/routine maintenance.” (circadian rhythm & MH clinician researcher)  “Arrange follow-ups after the therapy at short and longer term intervals if possible” (MH OT)  “Invite them three time a year for a special event and inform about new developments in therapy” (circadian rhythm & MH researcher) | 40 (71%) | 0 (0%) |

# Therapeutic approach and therapist factors

## Therapeutic approach, therapist attitude & manner

| Recommendation or issue raised | Data excerpts | professionals  n= (%) | personal exp.  n= (%) |
| --- | --- | --- | --- |
| An educational approach | “Education regarding the impact of being in bed too long” (sleep OT)  “Why drugs such as benzos or alcohol don’t work” (psychiatrist)  “role of the biological clock, light and darkness” (MH & circadian rhythm researcher)  “Information about sleep & good sleep practices” (sleep & MH researcher)  “Sleep psychoeducation (in contrast to e.g. standard insomnia treatment, where often people know a lot already!)” (sleep & MH researcher) | 34 (61%) | 0 (0%) |
|  | Education is also discussed specifically in relation to light, circadian rhythm, and regular rise time (above), and prior knowledge in these areas was rated as low by people with personal experience, as well as confidence that these approaches would work. | | |
| Education re: normal sleep | “What is normal sleep - many people I’ve assessed actually sleep fairly well but don’t realise it’s normal to wake several times per night and go straight back to sleep again” (psychiatrist)  “…helping people understand sleep and their sleep systems and why some of their beliefs are not accurate is a huge factor in their motivation to change their sleep behaviours.” (sleep OT CBTi practitioner) | 22 (39%) | 0 (0%) |
| Normalising | “normalising information on the variability of sleep for everyone” (sleep & MH researcher)  “Yes I think people can say that nightmares = going mad, but it might just be that your processing some stuff. […] [psychoeducation] include nightmares and role in processing memories.” (clinical psychologist) | 8 (14%) | 0 (0%) |
| Experimentation | “Encourage patients to see patterns (e.g. late wake time one day leads to poorer sleep and insomnia that night).  Obtain baseline sleep diary or passive monitoring data, and review together with patient, to gain insight into schedule.” (sleep specialist psychiatrist)  “…behavioural experiments with light in sessions (e.g. going outside and seeing how much that improves alertness/mood)” (sleep & MH researcher) | 5 (9%) | 0 (0%) |
| Benefits of change, motivational interviewing approach | “The benefits of it and link it to each person's goal achievement.” (MH OT)  “changing the view upon how time spend is a starting point” (sleep & MH researcher)  “Motivational interviewing for following through with treatment” (sleep & MH clinician researcher) | 11 (20%) | 0 (0%) |

| Recommendation or issue raised | Data excerpts | professionals  n= (%) | personal exp.  n= (%) |
| --- | --- | --- | --- |
| Therapeutic rapport & listening | “instilling hope for change will be an important part of building the rapport” (MH OT)  “must emphasize good connection/good relation with the patient” (MH & circadian rhythm clinician researcher) | 5 (9%) | 0 (0%) |
| Rapport required before home assessment | “I don't like strangers so interaction in the community first would be necessary” (personal experience)  “Too intrusive if it is a social worker. Social workers coming to your house are very judgemental - who would be doing the assessment and what does it entail.” (personal experience) | 0 (0%) | 6 (23%) |

## Therapist knowledge, skills & confidence

| Recommendation or issue raised | Data excerpts | professionals  n= (%) | personal exp.  n= (%) |
| --- | --- | --- | --- |
| Therapist confidence in delivering the intervention | “a lot of people say to me ‘if it wasn’t for how sure you were I couldn’t have done this’, so one think I always say […] is, never blink” (CBTi practitioner)  “Well usually I say that I know in practice for many patients this works so probably for you it will as well. So just go out and try it.” (sleep specialist psychiatrist)  “Encourage client that CBTi is well evidenced and researched to help people with insomnia.” (sleep OT)  “I would have thought the level of structure required may depend on the clinician's expertise and confidence, therefore, needs some flexibility across different levels of expertise in how the assessment is performed. That said, I would have thought it needs to be fairly structured across the board to be consistent in gathering information on what are determined the important areas” (MH OT) | 5 (9%) | n/a |
| Relationship to OT role & skills | “where others have specific, maybe psychology training or something far above what we have, we have those practical problem solving skills, its natural for us to look at ways to managing difficult activities in an easier way, and learning a new skill, its repetition, routine” (sleep OT CBTi practitioner)  “Alternatives to looking at screens in the evening. This is something I struggle with in patients who don't like reading, and OTs may be in a good position to explore this.” (psychiatrist)  “Barriers: OTs aren't aware it is in their domain, poor training of OT, not enough research” (sleep OT)  “ [Difficulties you would foresee with OTs delivering this] None. However in previous role it was clearly seen as the role of psychologists” (MH OT)  “[Difficulties you would foresee with OTs delivering this] Specialist therapy skills, unless training received. Eg. Socratic questioning, gentle cognitive restructuring, imagery rehearsal” (sleep & MH clinician researcher)  “you know how to be an OT, this is just an extension of what you already do […] sleep interventions. Its just extending OT to the 24hr day” (sleep OT) | 21 (38%) | n/a |
| Generic working barrier to OT interventions | “Occupational therapists who are working generically in the community are struggling to carry out occupational therapy interventions […] When testing the intervention I suggest targeting those occupational therapists who are already employed to provide occupational therapy interventions” (MH OT) | 3 (5%) | n/a |

# Implementation considerations

## Reaching referrals

| Recommendation or issue raised | Data excerpts | professionals  n= (%) | personal exp.  n= (%) |
| --- | --- | --- | --- |
| Reaching referrals | “MDT colleagues would not routinely consider sleep disturbance as an occupational issue” (MH OT)  “The biggest obstacle may be convincing the other professionals that this belongs in the OT treatment plan and is not left only to MDs/meds or psychologists” (sleep OT)  “a) Provide specific services addressing sleep. […] b) Provide a basic toolkit to professionals (so every team has someone with at least a basic knowledge of assessing and treating sleep in psychosis)” (sleep specialist psychiatrist)  “Include sleep questions in all assessments, ask the question (most people think it's about not enough sleep). Highlight the impact of poor sleep?” (MH OT)  “an advantage as OTs as when ppl are asked by the Dr to see a psychologist they might say ‘no theres nothing wrong with me I’m not crazy’, but when its do you want to work with the OT, they might be able to suggest some ways to help you, they say ‘sure’. […] its not really a therapy group […] focus on education.” (sleep OT)  “clients were more than willing to go into detail of their sleep problems and could very well pinpoint the problem” (circadian rhythm & MH researcher) | 35 (56%) | 0 (0%) |

## MDT approach within intervention

| Recommendation or issue raised | Data excerpts | professionals  n= (%) | personal exp.  n= (%) |
| --- | --- | --- | --- |
| MDT knowledge & attitude | “Changing the cultures of CMHTs to look at people’s quality of life, not just preventing/treating illness and making sure people comply with medication - big ask perhaps” (MH OT)  “Mental health professionals often view sleep problems as epiphenomena or symptoms associated with other diagnoses” (sleep OT)  “the social workers and occupational therapists, rather than the doctors were aware of the sleep disturbances but helpless in doing anything about it, because they were not having access to information and tools.” (circadian rhythm & MH researcher)  “Is sleep classed as a mental health? […] …so why doesn’t my psychiatrist treat me for insomnia? He won’t give me sleeping tablets. He won’t give me melatonin. He just treats me for this psychosis which I haven’t had for three years.” (personal experience) | 18 (32%) | 3 (12%) |

| Recommendation or issue raised | Data excerpts | professionals  n= (%) | personal exp.  n= (%) |
| --- | --- | --- | --- |
| MDT approach to intervention | “I have worked in multiple CMHTs. The difficulty is getting consistent and regular staff input to support the patients as they go through the process.” (sleep specialist psychiatrist)  “this needs to be generic to rehabilitation and not specific to OT” (sleep OT)  “Involvement of carers and support workers” (sleep & MH clinician researcher)  “addressing food and drink content […] people have complex medical issues and their diet (including fluid intake) should be carefully managed [not by OT but by dieticians] (sleep OT)  “Another example is having a dual diagnosis specialist who understands the role of drug use in sleep.” (sleep specialist psychiatrist) | 36 (64%) | 1 (4%) |
| MDT approach to medication | “In order to optimise outcomes, the team will need to work together to treat sleep. This will therefore require prescribers (usually psychiatrists) who understand sleep and the effect of different psychotropics on sleep, and work with the OT to optimise this.” (sleep specialist psychiatrist)  “Medication changes tend to pose some difficulties with determining whether a sleep strategy is working or not” (sleep & MH clinician researcher)  “napping is sometimes unavoidable due to medication, coordination between [C]MHT and OT important” (personal experience) | 25 (45%) | 1 (4%) |
| MDT approach to maintenance | “Engaging the person's social and other professional support network to continue any required prompting” (MH OT)  “Info for the usual clinicians on what has been covered in therapy, with resources to point the patient to if problems recur” (psychiatrist) | 9 (16%) | 0 (0%) |
