# Supplementary material for: A mixed methods expert opinion study on the optimal content and format for an occupational therapy intervention to improve sleep in schizophrenia spectrum disorders
Source: PLoS One. 2022 Jun 6;17(6):e0269453. doi: 10.1371/journal.pone.0269453 (PMC9170103; doi:10.1371/journal.pone.0269453)
Supplement: S2 File — (PDF) [file pone.0269453.s002.pdf]

Round 1:

About you

3. What is your name?

4. What is your job title / role / position?

5. Are you predominantly a clinician or researcher?
   - ◯ predominantly a clinician
   - ◯ predominantly a researcher
   - ◯ pretty much equally split, clinical academic

6. What groups do you work with or study?

   Select all those you regularly work with:
   - ☐ general population / healthy adults
   - ☐ depression (seasonal or non-seasonal)
   - ☐ bipolar
   - ☐ psychosis
   - ☐ personality disorder
   - ☐ anxiety disorders and phobias
   - ☐ sleep disorders - insomnia
   - ☐ sleep disorders - parasomnias and sleep disordered breathing
   - ☐ sleep disorders - circadian rhythm
   - ☐ neurodegenerative disorders
   - ☐ Other, please specify

7. **Your expertise:**
   We would like to identify the nature of your knowledge and experience, you could include information about the focus of the majority of your work as it relates to this project, and your professional background (if relevant). Please provide information in whatever way is easiest for you. E.g. If information is already on line you can paste a link to your institutional profile page, LinkedIn page, ResearchGate page, etc, and/or you can write in or paste in information.

   - This does not need to be exhaustive.

- We are **not** weighting responses based on qualifications or experience.
- We will use this information to best target further recruitment to fill in gaps and get a range of expertise within each group.
- We will use this information to help decide which group to include participants in, in cases where they fit into more than one group.

8. Please select which expert group you feel you best fit into:

*

- [ ] Sleep and/or circadian rhythm expert (clinician or researcher)
- [ ] Experienced mental health occupational therapy clinician or researcher
- [ ] Occupational therapist with expertise in sleep
- [ ] Other mental health expert or senior stakeholder
- [ ] I fit equally into two or more of these groups

9. What is the best email address to contact you with the link to complete round 2 and round 3?*

Your initial thoughts

This page is to capture your initial thoughts before we start to use any more specific headings which might alter your responses. There will be chance to elaborate on these later if you wish.

15. Do you have any views about what should be covered and how in the pre-intervention assessment?

This is the assessment that would be completed if the intervention were in routine clinical practice. An assessment to inform the therapy. We don't mean any baseline assessments which might be done primarily for the purposes of the research study.

1

2

3

16. Based on your knowledge and experience, what are the main aspects or components are which should be included in the intervention?

1

2

3

4

5

17. Do you have any thoughts about how to best help the service user maintain any improvements after therapy has ended?

1

2

3

For all of the free-text questions please feel free not to write in full sentences.

You are free to leave boxes blank if you have no idea or no opinion on that topic.

If you are arranging a phone / skype call (optional) you can leave some or all boxes blank and give verbal feeback on those instead.

The assessment

19. We have already determined that passive monitoring of rest-activity patterns will form part of the assessment, and later will form part of the intervention.

Do you have any comments or suggestions regarding this? (for example, how important is this, any drawbacks or challenges, any views about how to best approach this)

20. We have already determined that some form of recording of daytime activity will be invovled in the baseline assessment and later in the intervention.

Do you have any comments or suggestions regarding this? (for example, how important is this, any drawbacks or challenges, any views about how to best approach this)

21. We would ideally like to measure light exposure as part of the assessment and later durign the intervention, but as you may be aware there are methodological challenges in measuring light exposure accurately (devices worn onthe wrist are covered by clothes, devices struggle to account for factors like direction of gaze).

Do you have any comments or suggestions regarding this? (for example, how important is this, any drawbacks or challenges, any views about how to best approach this)

22. Do you have any comments or suggestions regarding technology or tools relating to any of the above (hardware, software, online or paper based)?

23. How long should the baseline assessment period be (roughly)? (i.e. the period within which sleep, and anything else, is measured and assessed before beginning intervention)

- ◯ assessment within first contact
- ◯ 1 week
- ◯ 2 weeks
- ◯ 3 weeks
- ◯ 4 weeks
- ◯ Other, please specify

Possible areas to address

24. How appropriate do you feel it is for the intervention to focus on the following areas:*

	**Very Appropriate**	**Appropriate**	**Neutral**	**Inappropriate**	**Don't know / no view on this**
sleep schedule	○	○	○	○	○
meal timing	○	○	○	○	○
daytime napping	○	○	○	○	○
daytime activity	○	○	○	○	○
bedroom / sleep area	○	○	○	○	○
light exposure	○	○	○	○	○
sleep interfering beliefs	○	○	○	○	○
waking up and morning routine	○	○	○	○	○
nightmares	○	○	○	○	○
evening and bedtime routine	○	○	○	○	○
antipsychotics and other medication	○	○	○	○	○
time in bed restriction / sleep restriction therapy	○	○	○	○	○
home environment	○	○	○	○	○

25. Do you feel there is anything missing from this list that you didn't already mention earlier? (response optional)

On the previous page you said:

· ____________________

· ____________________

· ____________________

26. Can you say any more about the reasons you feel it is better NOT to include any of the components you have selected as inappropriate above?

____

Page 13

27. What should be included in relation to light exposure and why?

Page 14

28. What should be included in relation to daytime activity and why?

Page 15

29. What should be included in relation to waking up and morning routine and why?

Page 16

30. What should be included in relation to evening and bedtime routine and why?

Page 17

31. What should be included in relation to daytime napping and why?

Page 18

32. What should be included in relation to sleep schedule and why?

Page 19

33. What should be included in relation to meal timing and why?

Page 20

34. What should be included in relation to time in bed restriction / sleep restriction therapy and why?

35. What should be included in relation to home environment and why?

36. What should be included in relation to bedroom / sleep area and why?

37. What should be included in relation to nightmares and why?

Page 24

38. What should be included in relation to sleep interfering beliefs and why?

Page 25

39. What should be included in relation to antipsychotics and other medication and why?

Page 26

40. Do you want to add any more detail about what should be included in relation to any of these answers you gave?

-
-
-
-

Page 27

Implementation questions

41. The intervention is aimed at people with schizophrenia spectrum disorders, who have problems with sleep initiation, maintenance, quality or timing.

Do you have any comments regarding for whom within this group the intervention would be most appropriate, or any exclusions you feel would need to be made?

42. (if relevant) What difficulties if any would you foresee with an occupational therapist in your service / in community mental health services you are familiar with delivering this intervention in future?

43. Do you have any comments regarding barriers to the delivery and effectiveness of the intervention, and how to avoid or overcome these?

44. Are you electing to give feedback verbally by phone or skype for this round? This can be general or just on a certain topic or question.

If so please give contact details and an indication of what days and times are best for you and I will get in touch.

Thank you very much for completing this survey!

I will be in touch with round 2 in late November.

Best Wishes,
Sophie
