# Supplementary material for: A mixed methods expert opinion study on the optimal content and format for an occupational therapy intervention to improve sleep in schizophrenia spectrum disorders
Source: PLoS One. 2022 Jun 6;17(6):e0269453. doi: 10.1371/journal.pone.0269453 (PMC9170103; doi:10.1371/journal.pone.0269453)
Supplement: S3 File — (PDF) [file pone.0269453.s003.pdf]

Round 2

Developing an Occupational Therapy intervention to improve sleep in people with schizophrenia spectrum disorders.

Welcome to round 2!

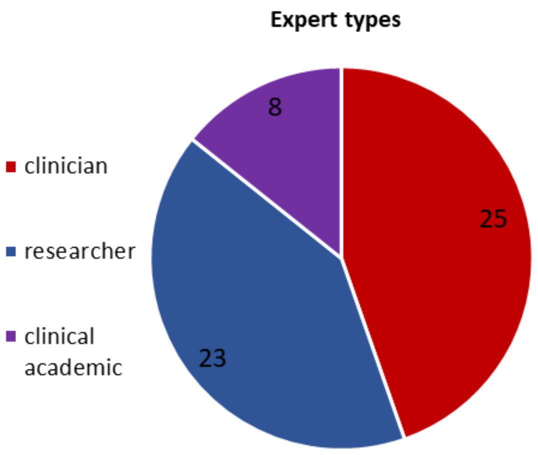

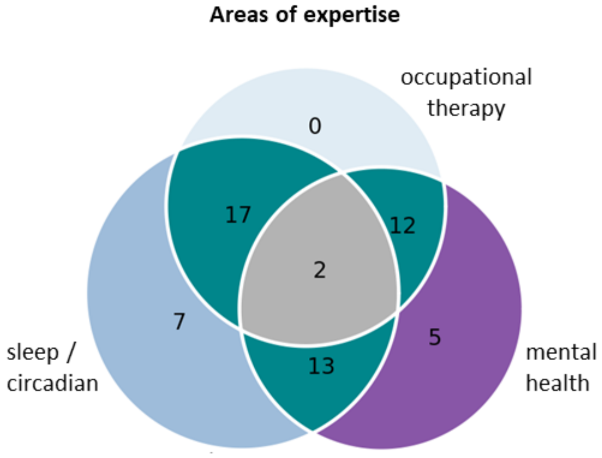

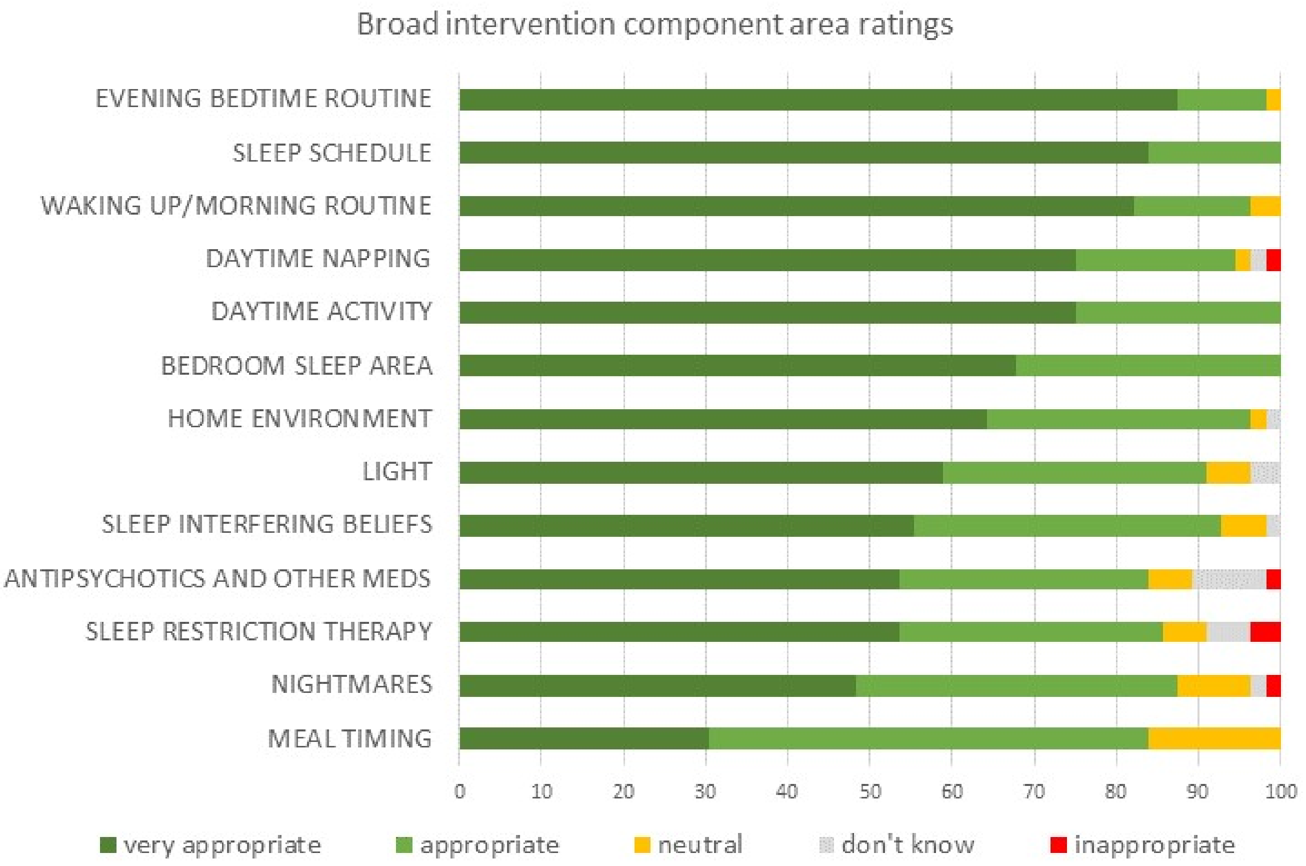

This survey will cover the following areas:

1. the initial assessment
2. the baseline assessment period
3. what should be included within the intervention components
4. which components are 'core' or 'optional'
5. order of componentsWe've put comments boxes throughout, but you needn't write in them all.

It should take you about half an hour, maybe less.
You can save and resume: press the 'save and resume button at the bottom of the page.

1 of 5: The initial assessment

◯ strongly agree
◯ agree
◯ neutral
◯ disagree
◯ strongly disagree

Assuming that there is limited time to complete the initial assessment, how should we prioritise the following broad areas?

Please rank:

	1st - give most time and effort	2nd	3rd	4th	5th	6th - give least time and effort
impact of problem, motivation to change sleep, expectations of intervention	◯	◯	◯	◯	◯	◯
nature and history of sleep complaint	◯	◯	◯	◯	◯	◯
medical and psychiatric condition	◯	◯	◯	◯	◯	◯
life context (social, occupational, physical, immediate and wider)	◯	◯	◯	◯	◯	◯
values, interests and priorities in life	◯	◯	◯	◯	◯	◯
medication, including client views	◯	◯	◯	◯	◯	◯

In round 1 many of you commented to the effect:
~You need to first identify the type of problem, and the context, to decide what to assess and what intervention to suggest.~

It is agreed that we need to find out the following early on:

is the problem with sleep initiation, broken sleep, unrefreshing sleep, sleep timing, something else
what aspects of sleep does the person want to change (if any?)
personal and social factors which dictate what sleep schedule is needed or desired
is the problem more insomnia or more circadian rhythm disorder (or both, or something else)
If you have anything to add, please write it here:

How important is it to cover the following in the initial assessment?

	very important	important	neutral	not important	better NOT to include
medical co-morbidities / physical illnesses	◯	◯	◯	◯	◯
mental health condition and symptoms (when ill, when well, current)	◯	◯	◯	◯	◯
history of sleep complaint	◯	◯	◯	◯	◯
family history of sleep complaints	◯	◯	◯	◯	◯
self-report triggers for sleeping difficulties	◯	◯	◯	◯	◯
assess chronotype / circadian preference (owl or lark)	◯	◯	◯	◯	◯

It is agreed:
We will screen for sleep disordered breathing, restless leg syndrome, movement disorders and other parasomnias. Many of you mentioned this. Also it would be wrong not to. (Thank you for suggestions regarding screening tools.)

How important is it to cover the following in the initial assessment?

	very important	important	neutral	not important	better NOT to include
social environment (immediate and wider)	◯	◯	◯	◯	◯
overview of activities and routines	◯	◯	◯	◯	◯
interests and priorities in waking life	◯	◯	◯	◯	◯

	very important	important	neutral	not important	better NOT to include
medication(s) (what drug)	◯	◯	◯	◯	◯
medication timing(s)	◯	◯	◯	◯	◯
medication dose(s)	◯	◯	◯	◯	◯
person's view of medications and their role(s)	◯	◯	◯	◯	◯
person's view of medication's impact on sleep	◯	◯	◯	◯	◯

	very important	important	neutral	not important	better NOT to include
effect on daytime functioning sleep	○	○	○	○	○
assess motivation to improve sleep	○	○	○	○	○
can the person tolerate intervention without immediate benefit (sleep might be worse initially)?	○	○	○	○	○
what have they already tried?	○	○	○	○	○
what are their expectations and hopes for intervention?	○	○	○	○	○

2 of 5: The baseline assessment period

Better for baseline assessment period to depend on variability of presentation	Better to use a set length of baseline assessment period

(Place a mark on the scale above)

If we vary the length of the baseline assessment period, how much should it vary?

Shortest length

- ◯ 1 week
- ◯ 2 weeks
- ◯ 3 weeks
- ◯ 4 weeks
- ◯ 5 weeks
- ◯ more than 5 weeks

Longest length

- ◯ 1 week
- ◯ 2 weeks
- ◯ 3 weeks
- ◯ 4 weeks
- ◯ 5 weeks
- ◯ more than 5 weeks

If you have anything to add, please write it here:

3 of 5: Intervention components

Evening routine

It is agreed:
encourage to reduce stimulus and have evening wind down time
ensure following sleep hygiene recommendations as far as possible

Please rate:

	very important	important	neutral	not important	better NOT to include
support to establish evening routine which is similar each night	○	○	○	○	○
set time to start bed preparation	○	○	○	○	○
support to find suitable relaxing activities for evenings, provide ideas and materials if needed	○	○	○	○	○
encourage preparation for the next day the evening before	○	○	○	○	○

If you have anything to add, please write it here:

Morning routine

It is agreed:
give psychoeducation on sleep inertia - that sleepiness immediately on waking does not always reflect inadequate quality or amount of sleep
encourage energising activities such as shower, dress, go outside if possible

Please slide:

regular rise time
to anchor the circadian rhythm

could sometimes sleep in to recover sleep debt
if not enough sleep time

(Place a mark on the scale above)

Please slide:

continue with strictly regular rise time
to anchor the circadian rhythm

can afford occasional flexibility once routine is established

(Place a mark on the scale above)

Alarm clocks
(select all that would sometimes be appropriate)

- ☐ support to set alarms
- ☐ include morning phone calls in intervention
- ☐ multiple alarms and set away from the bed
- ☐ puzzle alarm clocks (usually a mobile app)
- ☐ dawn simulation alarm clock
- ☐ avoid using alarm clocks

If you have anything to add, please write it here:

Sleep restriction therapy / time in bed restriction / sleep efficiency training.

	strongly agree	agree	neutral	disagree	strongly disagree
do not use any version of time in bed restriction	○	○	○	○	○
use adapted sleep restriction with a longer minimum sleep window	○	○	○	○	○
use sleep compression instead of sleep restriction	○	○	○	○	○

How long should the MINIMUM sleep window be (the shortest that should ever be used in this intervention)?

response optional

○ 5.5hrs ○ 6hrs ○ 6.5hrs ○ 7hrs ○ 7.5hrs ○ 8hrs ○ 8.5hrs ○ 9hrs ○ 9.5hrs
○ 10hrs

If you have anything to add, please write it here:

Napping

It is agreed:
  avoid naps too late in the day
avoid longer naps
  consider scheduling a nap midday or early afternoon if it is the only way to avoid a nap later in the day
Please rate:

	strongly agree	agree	neutral	disagree	strongly disagree
Allow napping	○	○	○	○	○
Avoid napping	○	○	○	○	○
Ban napping	○	○	○	○	○
Evaluate the role of naps	○	○	○	○	○
Encourage a regular, planned nap	○	○	○	○	○
Replace naps with activities...	○	○	○	○	○
...replace naps with active energising activities	○	○	○	○	○
...replace naps with restorative calming activities	○	○	○	○	○

If you have anything to add, please write it here:

Modifying light exposure

	very important	important	neutral	not important	better NOT to include
use a light box (in some cases)	○	○	○	○	○
use outdoor light (if possible)	○	○	○	○	○
address window coverings, curtains, blinds	○	○	○	○	○
use amber glasses (blue-light blocking) in the evening	○	○	○	○	○
alter the lighting in the home, e.g. change bulbs, arrange low light lamps for evening and night-time	○	○	○	○	○

If you have anything to add, please write it here:

Home assessment and intervention

	very important	important	neutral	not important	better NOT to include
Address sense of security in the home	◯	◯	◯	◯	◯
Try to improve air quality if mould, damp or allergens are an issue	◯	◯	◯	◯	◯
Consider use of ambient noise / white noise in bedroom	◯	◯	◯	◯	◯
Alter locations of items so that the bed (and ideally the bedroom) is only used for sleep and sex	◯	◯	◯	◯	◯

If you have anything to add, please write it here:

Activity and occupation

	very important	important	neutral	not important	better NOT to include
support to set "do not disturb" settings on devices	◯	◯	◯	◯	◯
continue with activities planned irrespective of sleep	◯	◯	◯	◯	◯
identify appropriate activities to do if waking in the night	◯	◯	◯	◯	◯

If you have anything to add, please write it here:

Food and drink

It is agreed:
  More relevant to refer to food and drink than to 'meals'
  Psychoeducation on why to avoid large late meals
  Don't go to bed hungry either (light snack 1 - 1.5hrs before bed if hunger disturbs sleep)

Please rate

	very important	important	neutral	not important	better NOT to include
address food routines earlier in the day to avoid eating a big meal late at night	◯	◯	◯	◯	◯
address content of food and drinks	◯	◯	◯	◯	◯
avoid snacking	◯	◯	◯	◯	◯

If you have anything to add, please write it here:

Substance use (caffeine, alcohol, nicotine, illicit substances)

It is agreed:
reduce caffeine use, especially late in day
reduce smoking late at night, discourage smoking if awakening in night
psychoeducation on the effect of alcohol on sleep

If you have anything to add, please write it here:

Medication

It is agreed:
ensure taken at correct timing - morning or evening
liaise with prescriber, generally and regarding any sleep affecting side effects (e.g.daytime sedation, hypersalviation)

Please rate:

	very important	important	neutral	not important	better NOT to include
Experiment with altering exact timing of oral medication (e.g. earlier / later in the evening)	◯	◯	◯	◯	◯

If you have anything to add, please write it here:

Nightmares

Although some of you suggested delivering imagery rehearsal or re-scripting, more respondents felt specialist nightmare intervention was beyond the scope of this intervention.

It is agreed:
nightmares will be assessed, refer or liaise as indicated
psychoeducation to normalise occasional nighmares or bad dreamsSome noted that nightmares may improve through treatment of other sleep problems.

Please rate:

	very important	important	neutral	not important	better NOT to include
work on strategies to calm self to return to bed if waking with a nightmare	◯	◯	◯	◯	◯
if hypersomnia (e.g. over 9hrs), work on reducing to 8hrs	◯	◯	◯	◯	◯

If you have anything to add, please write it here:

New components suggested

	very important	important	neutral	not important	better NOT to include
thermoregulation - hot bath or shower in evening (not if hot weather)	◯	◯	◯	◯	◯
thermoregulation - ensure wearing evening footwear	◯	◯	◯	◯	◯
mindfulness meditation	◯	◯	◯	◯	◯
relaxation - breathing techniques	◯	◯	◯	◯	◯
relaxation - progressive muscle relaxation	◯	◯	◯	◯	◯
relaxation - guided imagery	◯	◯	◯	◯	◯
Address sensory qualities of pyjamas and bedding	◯	◯	◯	◯	◯
Address sensory qualities of room: colours, chaotic vs calm appearance	◯	◯	◯	◯	◯

If you have anything to add, please write it here:

Cognitive approaches to worry and rumination were suggested, particularly by people trained in CBT for insomnia, but some note not all occupational therapists would be confident in this area.

POINTS FOR:
  Usually included within CBT for insomnia.
  Worry will be an issue for some clients.
POINTS AGAINST:
  Maybe better matched to the skills of psychologists.
Cognitive approaches not as essential within CBT for insomnia as in other forms of CBT.

To what extent should cognitive or psychological approaches be included?

fully, with no modifications	included to some extent, with some modifications	not at all

(Place a mark on the scale above)

If you have anything to add, please write it here:

4 of 5: Core vs optional components

Core componentswould be those which are relevant for only certain cases (for example if reducing napping was an optional component, you might only attempt to reduce napping under certain conditions, such as if a particular complaint was present, or if naps were of a certain type/length/timing)

Please rate:

	core component (always use)	optional component (sometimes use)	do not use
nightmares (just in so far as described above)	◯	◯	◯
relaxation techniques	◯	◯	◯
psychoeducation regarding normal sleep	◯	◯	◯
thermoregulation	◯	◯	◯
sensory factors	◯	◯	◯

	core component (always use)	optional component (sometimes use)	do not use
food & drink	◯	◯	◯
substance use	◯	◯	◯
morning routine	◯	◯	◯
evening routine	◯	◯	◯
address napping	◯	◯	◯
sleep schedule modifications	◯	◯	◯

	core component (always use)	optional component (sometimes use)	do not use
address medication	◯	◯	◯
home and bedroom intervention / adaptations	◯	◯	◯
activity and occupation	◯	◯	◯
modify light exposure patterns	◯	◯	◯
cognitive approaches to worry	◯	◯	◯

5 of 5: Order of delivery

to what extent should there be a pre-established order?

There should be a pre-established order of delivery — The order should be totally determined by the individual circumstances

(Place a mark on the scale above)

Please select:

	should be delivered first / early on	should be delivered around the middle	should be delivered later on / last	doesn't matter when
nightmares (just in so far as described above)	○	○	○	○
relaxation techniques	○	○	○	○
psychoeducation regarding normal sleep	○	○	○	○
thermoregulation	○	○	○	○
sensory factors	○	○	○	○

	should be delivered first / early on	should be delivered around the middle	should be delivered later on / last	doesn't matter when
food & drink	○	○	○	○
substance use	○	○	○	○
morning routine	○	○	○	○
evening routine	○	○	○	○
address napping	○	○	○	○
sleep schedule modifications	○	○	○	○

	should be delivered first / early on	should be delivered around the middle	should be delivered later on	doesn't matter when
address medication	○	○	○	○
home and bedroom intervention / adaptation	○	○	○	○
activity and occupation	○	○	○	○
modify light exposure patterns	○	○	○	○
cognitive approaches to worry	○	○	○	○

If you have anything to add, please write it here:

DONE!
