# Supplementary material for: A mixed methods expert opinion study on the optimal content and format for an occupational therapy intervention to improve sleep in schizophrenia spectrum disorders
Source: PLoS One. 2022 Jun 6;17(6):e0269453. doi: 10.1371/journal.pone.0269453 (PMC9170103; doi:10.1371/journal.pone.0269453)
Supplement: S4 File — (PDF) [file pone.0269453.s004.pdf]

Round 3 survey (final round): ask the experts

Welcome to Round 3!
(the final round)

Thanks again for your responses in rounds 1 & 2. They were really great!

A high level of consensus has been reached on some items to include. We are only asking about selected topics. Sometimes re-rating, other times we are exploring further how to address this component. It is always OK if your view has not changed. We are not expecting to reach a consensus on all items.

Paraphrased comment are ~"presented like this"

Items agreed based on round 2 will be presented in green boxes.
Points which we already described as 'agreed' in the last round (agreed from round 1) will be presented in yellow boxes.
Judging consensus was based on ratings of 'importance' and 'agreement', and the extent of votes in the opposite direction. Qualitative comments were also taken into account.

First a summary of where we are up to so far.

This survey will cover:

1. Eligibility (who the intervention is aimed at)
2. Intervention components questions

3. OPTIONAL: Additional feedback, comment optionalEND - select gift voucher & contact preferences

It should take you around 30min to complete (longer if you comment on the optional section). You can press 'save and resume' at any time. You will be able to return and read more and edit or add to your response until we close round 3, even after you have pressed 'finish'.

There are many optional comments boxes throughout, you are welcome to write in as many or as few as you want.

1) Eligibility (who is the intervention aimed at)

For background

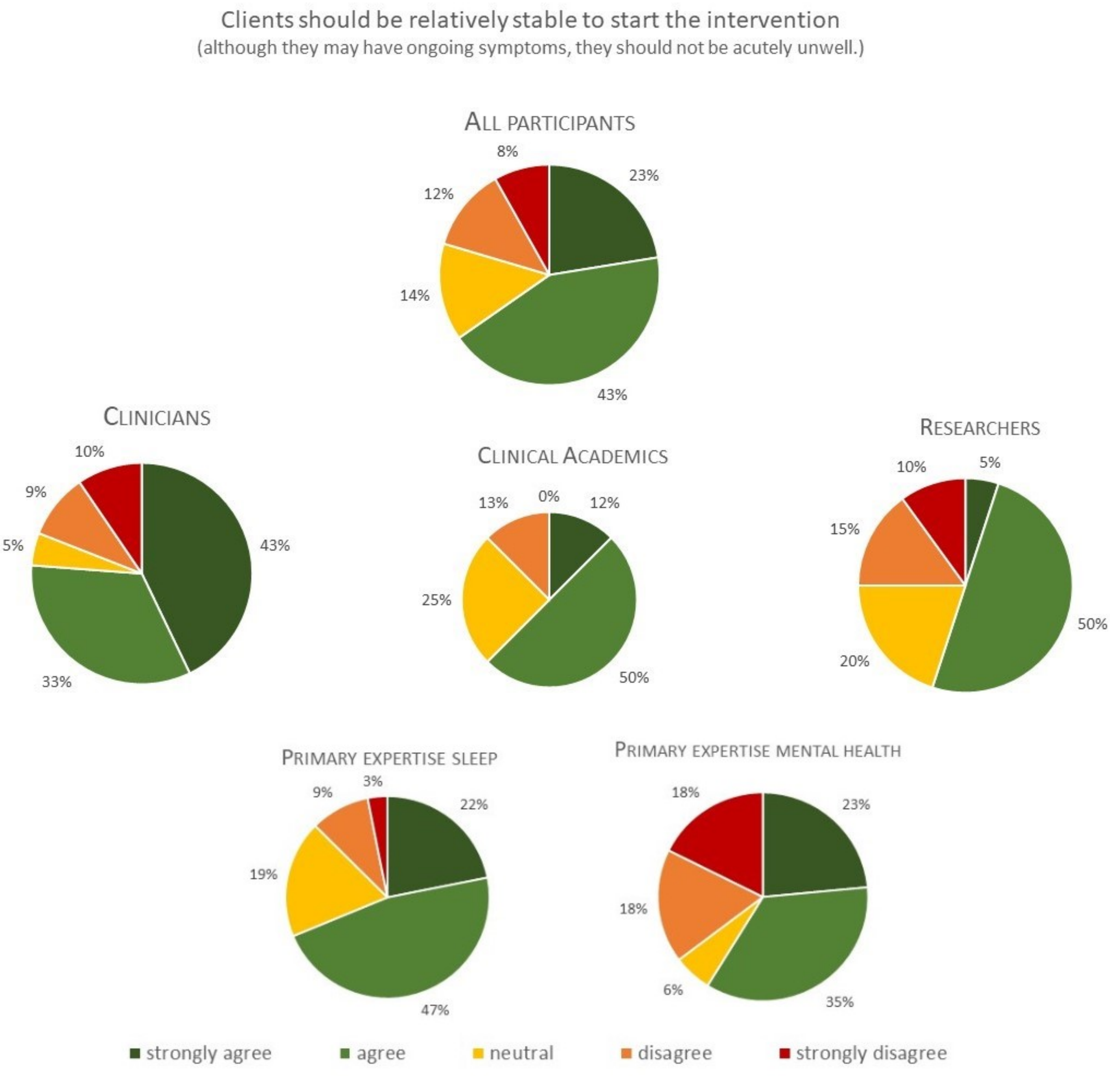

If you have concerns about imposing inclusion criteria or using a clinical assessment to determine whether the client is stable enough to usefully begin the intervention, please can you describe?

- ◯ strongly agree
- ◯ agree
- ◯ neutral
- ◯ disagree
- ◯ strongly disagree
- ◯ don't know / no view on this

2) Intervention components questions

Food & drink
Agreed:Education regarding why to avoid large late meals
Don't go to bed hungry either (light snack 1 - 1.5hrs before bed if hunger disturbs sleep)

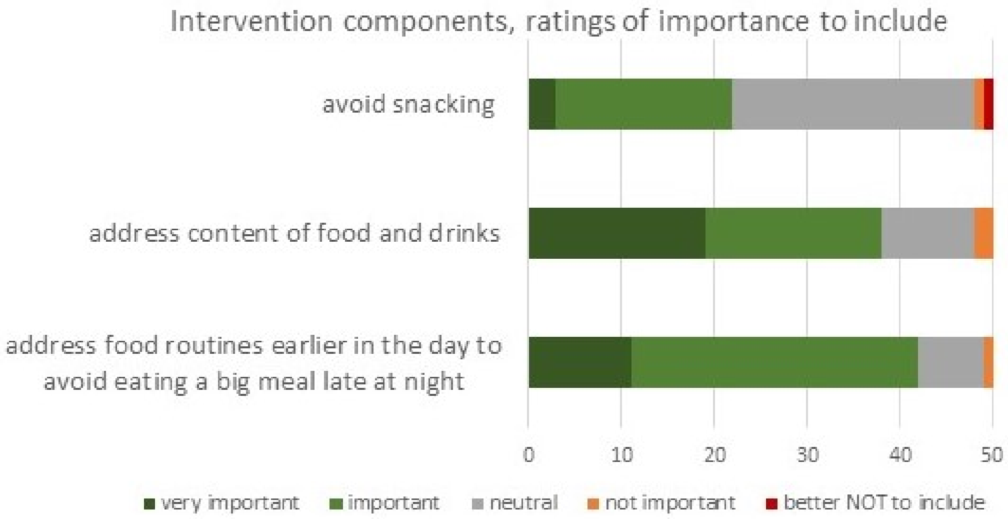

	very important	important	neutral	not important	better NOT to include
address food routines earlier in the day to avoid eating a big meal late at night	◯	◯	◯	◯	◯
address content of food and drinks (e.g. how filling, fat, sugar, portion size)	◯	◯	◯	◯	◯
avoid snacking (unless medical condition requires)	◯	◯	◯	◯	◯

Sleep restriction / time in bed restriction / extension of time up and awake

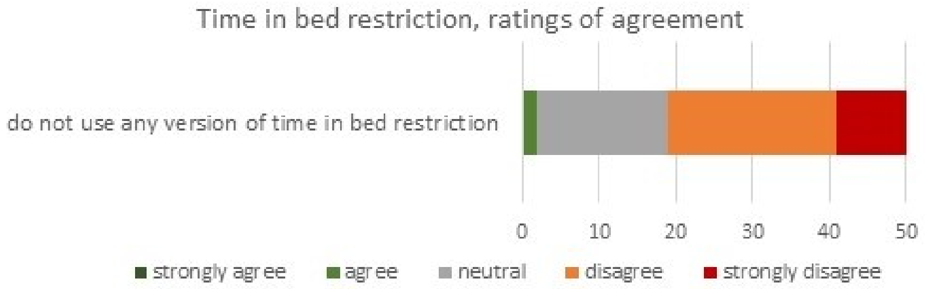

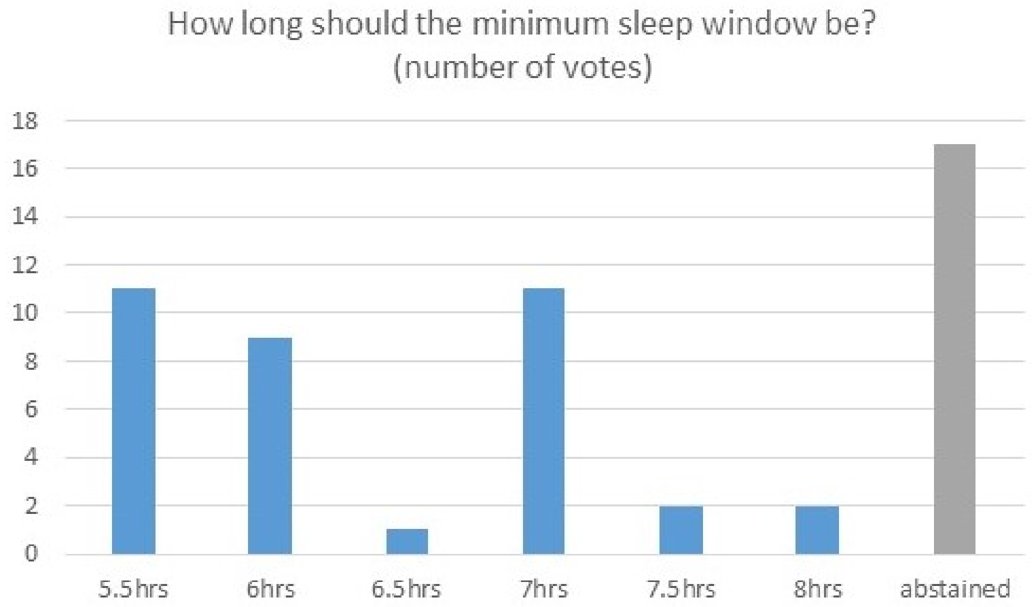

(some felt they often do, others felt they do not).
~"Even a small amount of time in bed restriction may be effective."
~"Limited research on effectiveness in this population."
~"Limited evidence of risk of causing psychosis."
~"In practice the limiting factor may not be contra-indications and risk, but what participants can manage to adhere to."

The below were suggested. Do you agree?

	strongly agree	agree	neutral	disagree	strongly disagree	don't know / no view on this
~"The length of the sleep window should take into account not just the total sleep time at baseline, but also the length of the time in bed at baseline"	○	○	○	○	○	○
~"The actual terminology of sleep restriction or time in bed restriction should be avoided"	○	○	○	○	○	○
~"Terminology and presentation should focus on the 'positive' of time you are up and awake rather than time you are not in bed."	○	○	○	○	○	○

~"People taking anti-psychotics may need to sleep for longer."

How long should the MINIMUM sleep window be (the shortest that should ever be used in this intervention)?

includes options 'don't know / no view on this' and 'do not use'

○ 5.5hrs ○ 6hrs ○ 6.5hrs ○ 7hrs ○ 7.5hrs ○ 8hrs ○ longer than 8hrs ○ better NOT to use time in bed restriction ○ don't know / no view on this

Medication
Agreed:ensure taken at correct timing - morning or evening
liaise with prescriber - generally, and regarding any sleep affecting side effects (e.g.daytime sedation, hypersalivation)~"Obtain this information from clinical records in advance if possible"
~"Spend less time discussing a medication if there is no realistic prospect of altering this medication"

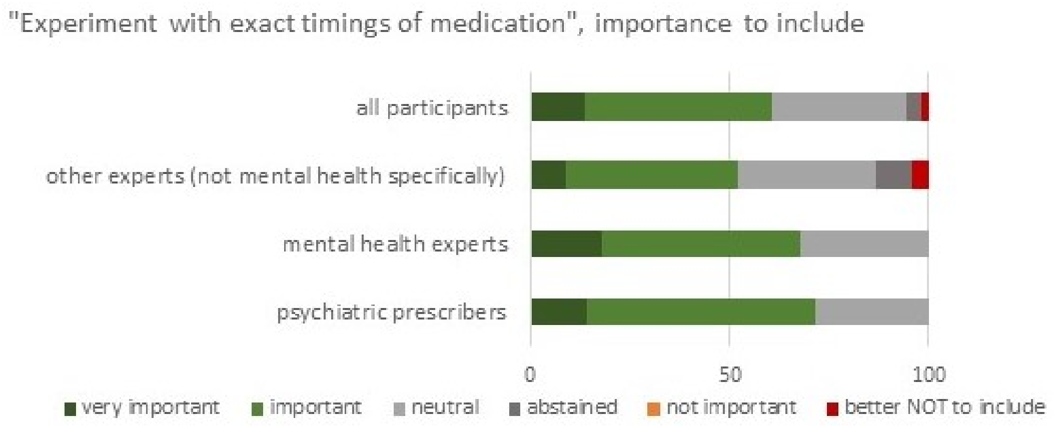

◯ very important ◯ important ◯ neutral ◯ not important ◯ better NOT to include
◯ don't know / no view on this

Intervention components still under consideration

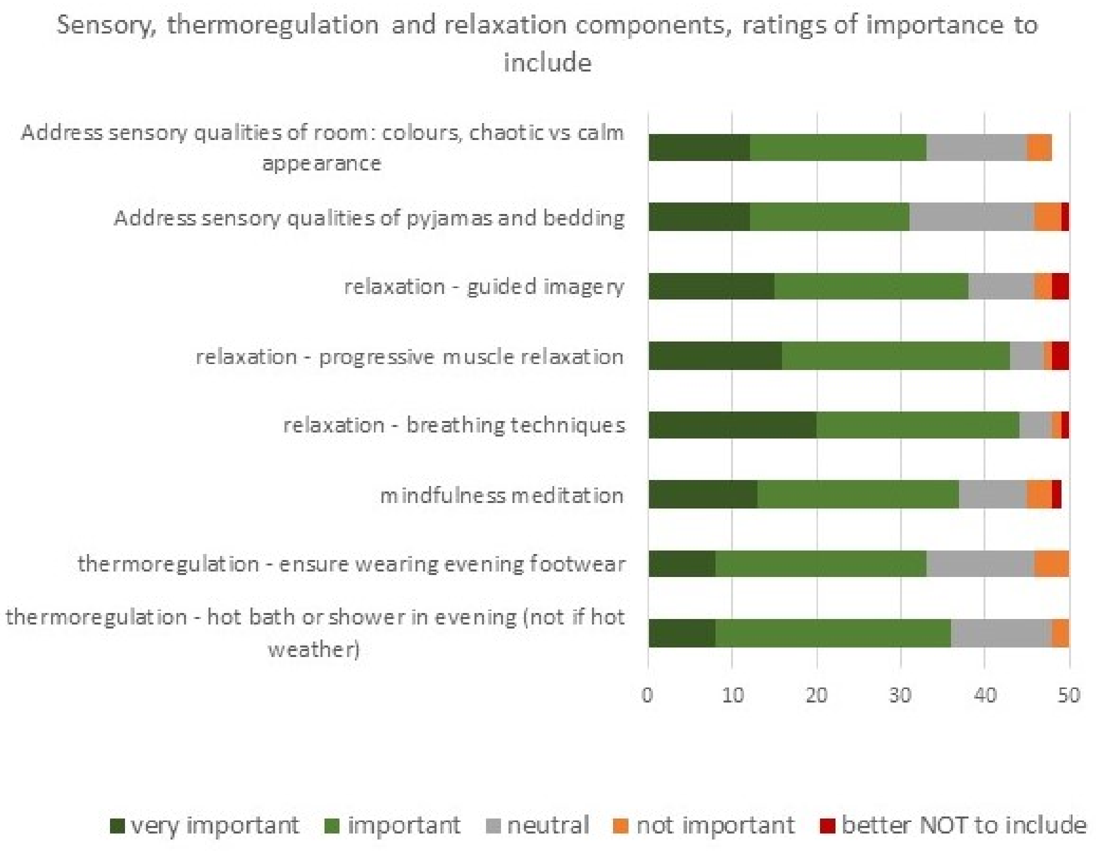

Sensory factors

	strongly agree	agree	neutral	disagree	strongly disagree
I have knowledge and / or experience on this topic	◯	◯	◯	◯	◯

	very important	important	neutral	not important	better NOT to include
address sensory qualities of room: colours, chaotic vs calm appearance	◯	◯	◯	◯	◯
address sensory qualities of pyjamas and bedding	◯	◯	◯	◯	◯

Thermoregulation
(agreed, discussed without using the word thermoregulation within the home environment component)

	strongly agree	agree	neutral	disagree	strongly disagree
I have knowledge and / or experience on this topic	◯	◯	◯	◯	◯

	very important	important	neutral	not important	better NOT to include
thermoregulation - ensure wearing evening footwear	◯	◯	◯	◯	◯
thermoregulation - hot bath or shower in the evening (not if hot weather)	◯	◯	◯	◯	◯

Relaxation

	strongly agree	agree	neutral	disagree	strongly disagree
I have knowledge and / or experience on this topic	◯	◯	◯	◯	◯

	very important	important	neutral	not important	better NOT to include
relaxation - guided imagery	◯	◯	◯	◯	◯
relaxation - progressive muscle relaxation	◯	◯	◯	◯	◯
relaxation - breathing techniques	◯	◯	◯	◯	◯
mindfulness meditation	◯	◯	◯	◯	◯

PAGE 3 IS ADDITIONAL FEEDBACK, AND CAN BE SKIPPED OR SKIMMED.
If skipping please proceed to pages 5 & 6.
You can return to this survey later with the same link if you want to look at this another time, you will be able to edit or add to your response until we close round 3, even after you have pressed 'finish'.

3. Additional feedback, comment optional, can skip

Assessment:
Here are the ratings of importance of the assessment topics:

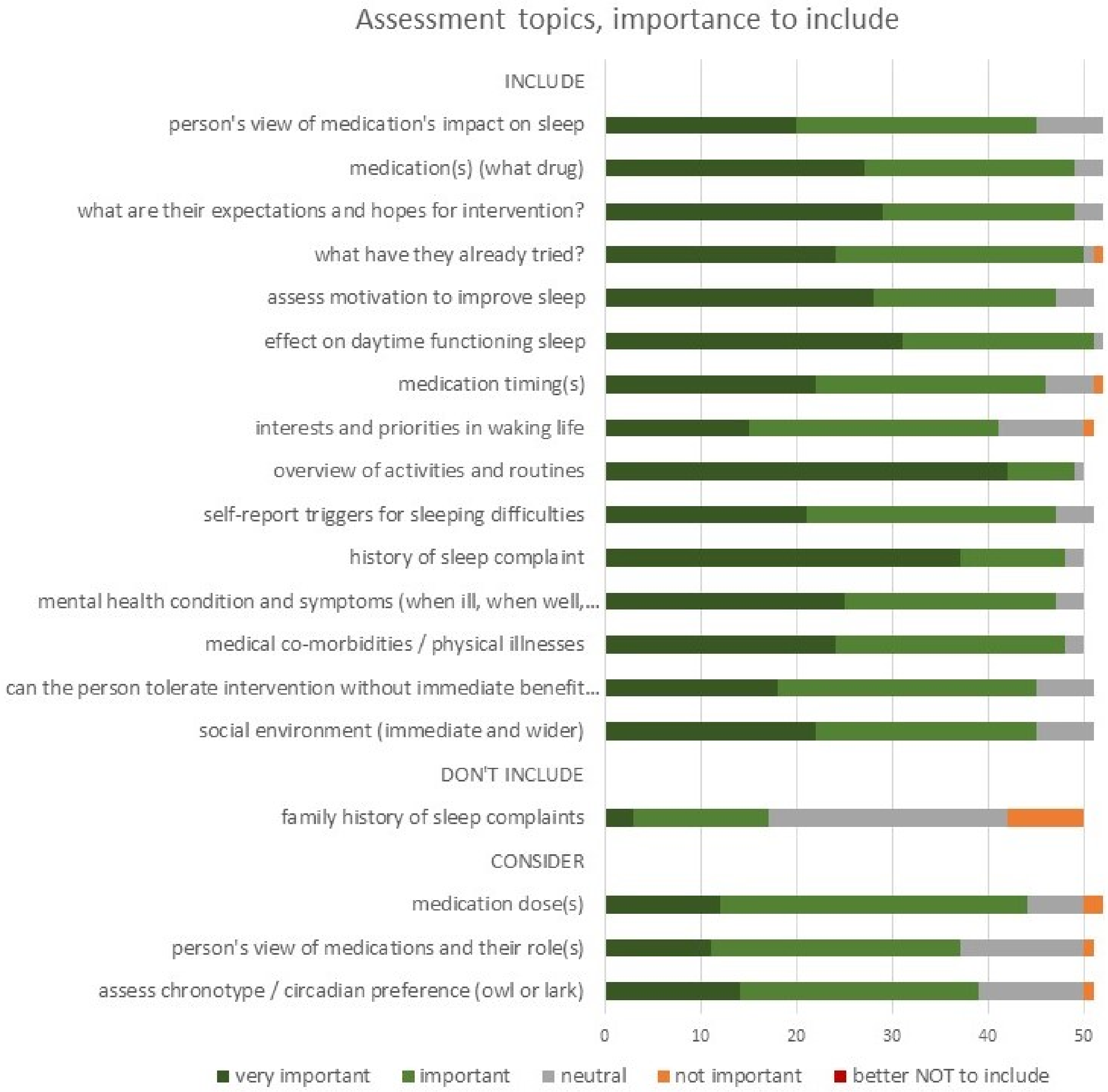

Also agreed:
As part of assessing food routines we will ask about night eating.
If the person follows a religion we will ask about the timing or prayers or other practices (including fasting if relevant) to be taken into account in relation to routines. (This will form part of the 'overview of activities and routines', but merits its own prompt within the protocol/materials)

Light exposure:
  Increase morning light
  Increase daytime light
  Outdoor light especially good
  Reduce evening light exposure
  Incorporate with activity/occupation
Give education and explanation of how light affects circadian rhythm

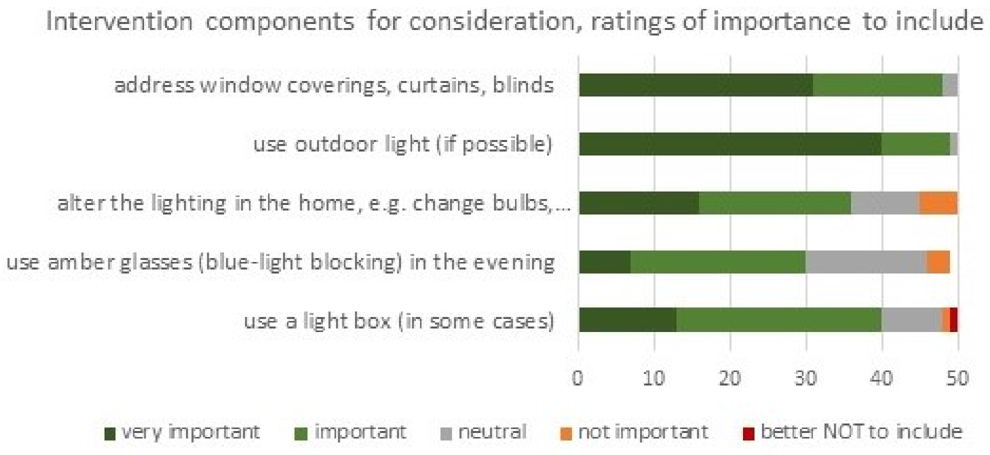

Address window coverings (e.g. windows and blinds)
 Use outdoor light (if possible)

Rise time

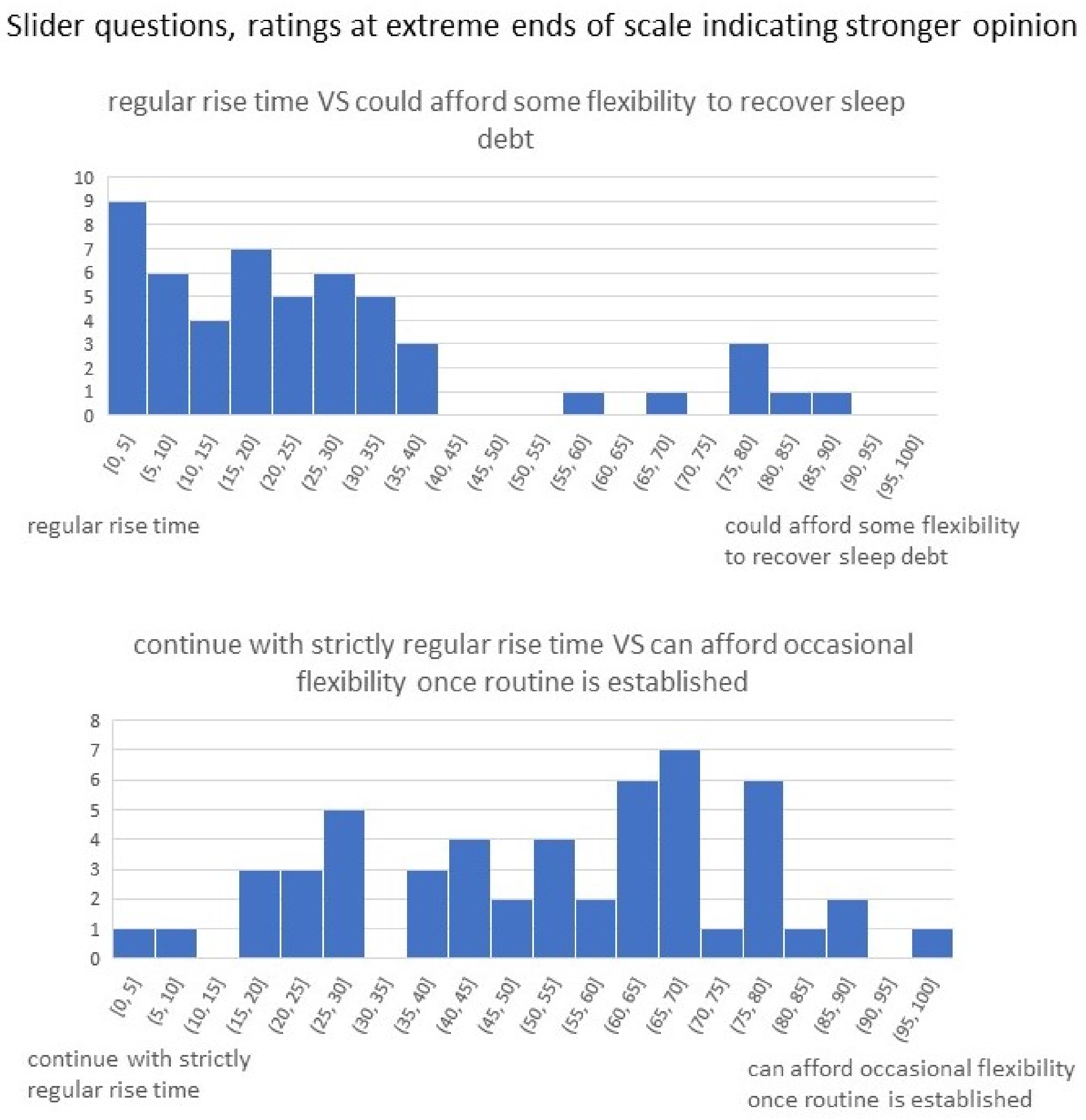

Regularity should be the aim, especially during the intervention.
Regularity should not be at any cost- i.e. if the only way to achieve regular timing is for sleep to be regularly timed but continuously insufficient, it would be better to eventually revert to napping or irregular timing.
If you have any further comments, please write them here:

Activity and occupation:
Encourage daytime exercise / physical activity, to increase sleep pressure
Reviewing activity diary with client may form an intervention in itself
  Activity analysis - includes emotional, cognitive and physical stimulating / calming effect of activities, and light exposure during.
  Collaborative activity scheduling if required

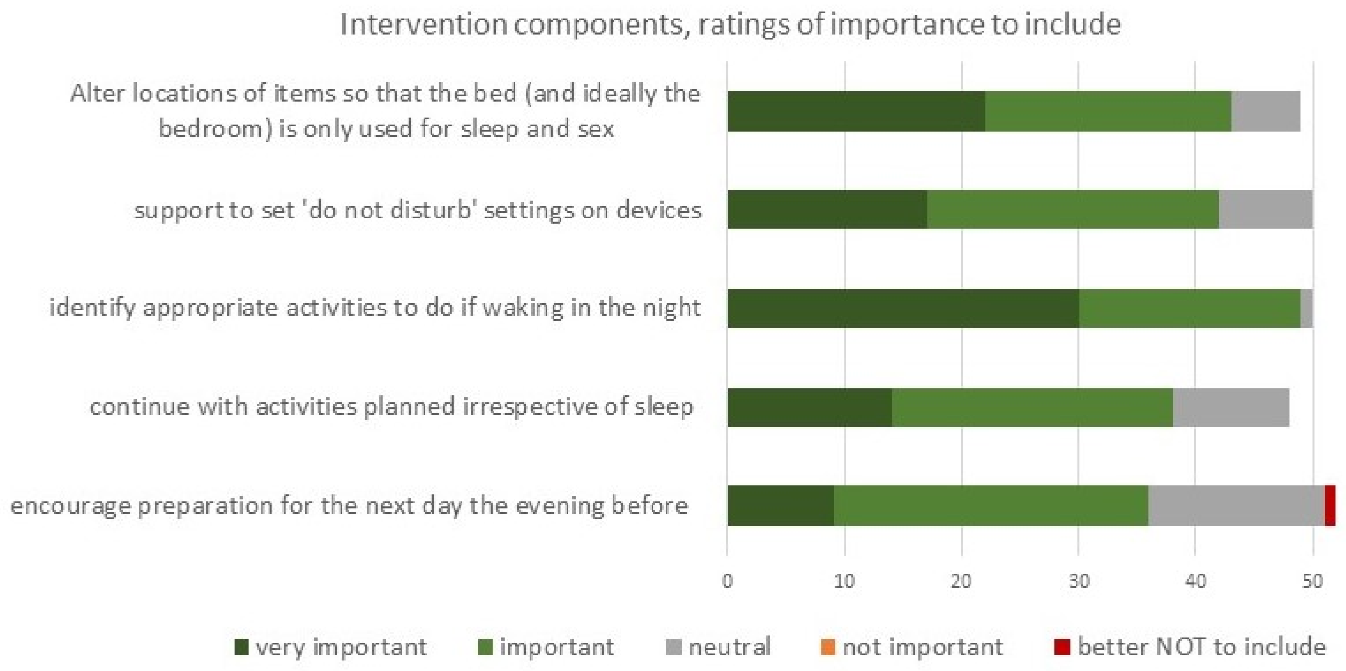

Alter locations of items so that the bed (and ideally the bedroom) is only used for sleep and sex
Support to set do not disturb setting on devices
Support to find appropriate activities to do if waking in the night

~"(This varies between individuals) preparing for the next day could make some people more anxious"
~"It is easier to get up if things are in place for the morning, e.g. breakfast, clothes, alarm"

Napping:
Avoid naps too late in the day
Avoid longer naps
Consider scheduling a nap midday or early afternoon if it is the only way to avoid a nap later in the day

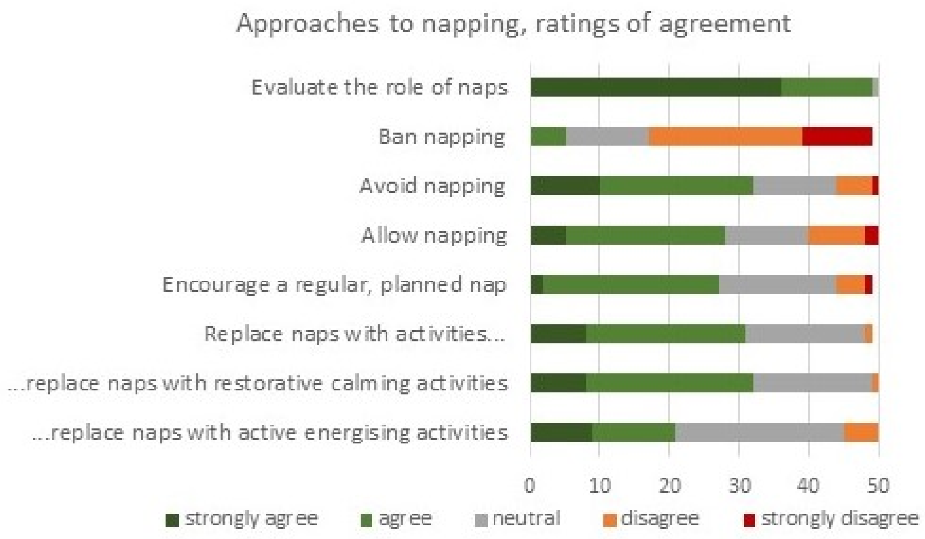

Evaluate the role of naps
Do not ban naps

Alarms:
If you have any further comments, please write them here:

Evening routine:Encourage to reduce stimulus and have evening wind down time
Ensure following sleep hygiene recommendations as far as possible

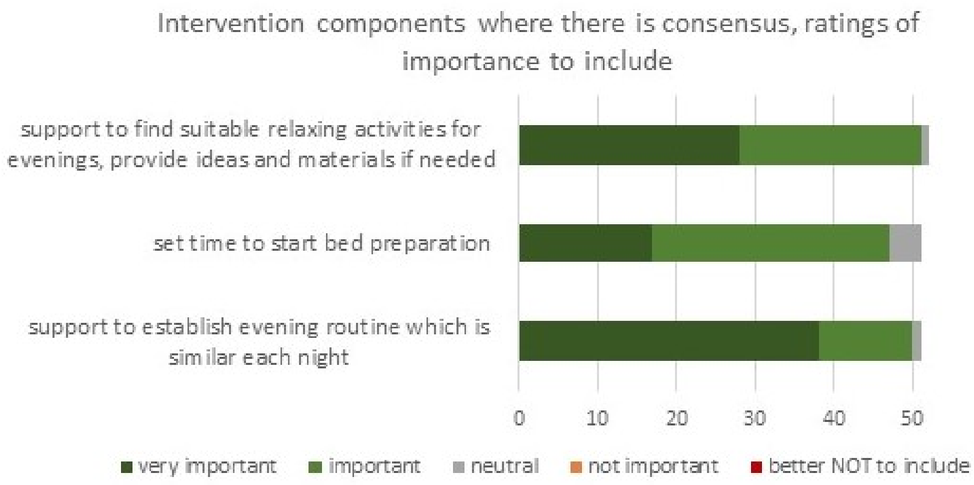

Support to establish evening routine which is similar each night
Support to find suitable relaxing activities for evenings, provide ideas and materials if needed
Set time to start bed preparation

Morning routine:
Include attention to:
Getting dressed
Going outdoors if possible
Increasing light exposure soon after waking
Energising activities to help wake up and get going
Ideally physical activity if possible
Give education on sleep inertia - that sleepiness immediately on waking does not always reflect inadequate quality or amount of sleep

---

Substance use:
Reduce caffeine use, especially late in day
Reduce smoking late at night, discourage smoking if awakening in night
Psychoeducation on the effect of alcohol on sleep
~"Bear in mind individual differences in metabolism and sensitivity to caffeine and other substances"

Home environment:
Home environment will be assessed with client agreement
Explicitly ask where sleep takes place (may not be the bed)
Consider and address: noise, temperature, other occupants, pets, comfort
Identify modifications which can be made to improve the above, set goals, offer support
If you have any further comments, please write them here:

Nightmares:
Nightmares will be assessed, refer or liaise as indicated
Psychoeducation to normalise occasional nightmares or bad dreams
Nightmares may improve through treatment of other sleep problems.

Psychological components:

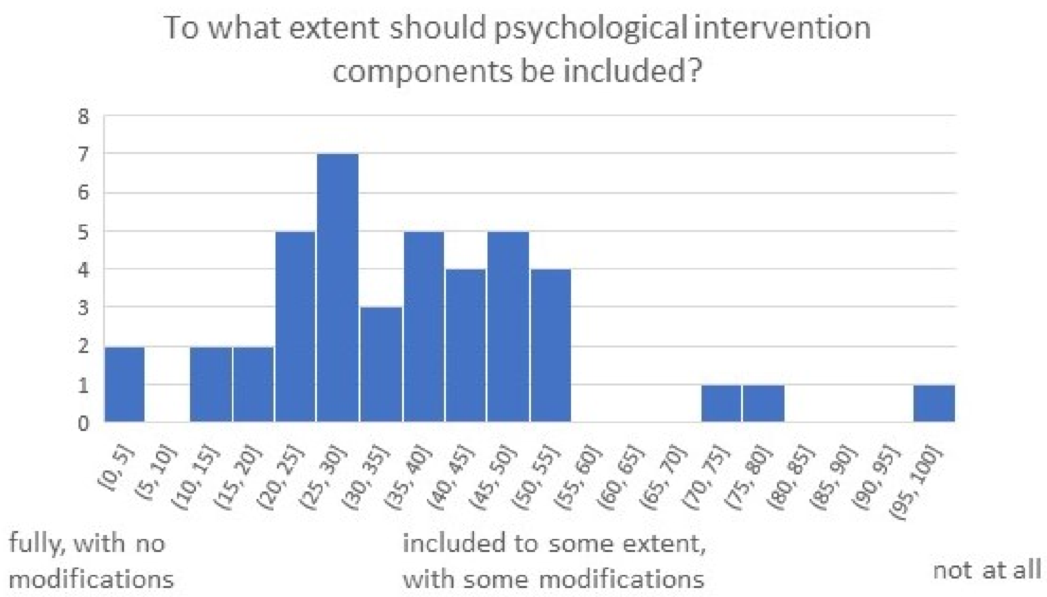

~"There is enough to address in terms of environment, behaviours and routines"
~"Dealing with worry will be very important for some clients"
~"Strategies to manage worry are already relevant to the role of mental health occupational therapists"

4) The baseline period

Please note that a long baseline period would not require constant self-report recording by the participant, only intermittent self-report recording, and constant use of an activity tracking wearable device ("passive monitoring"; the person just wears the device and has to take no action.)

Here is a graph of the votes for proposed shortest and longest length of the baseline period.

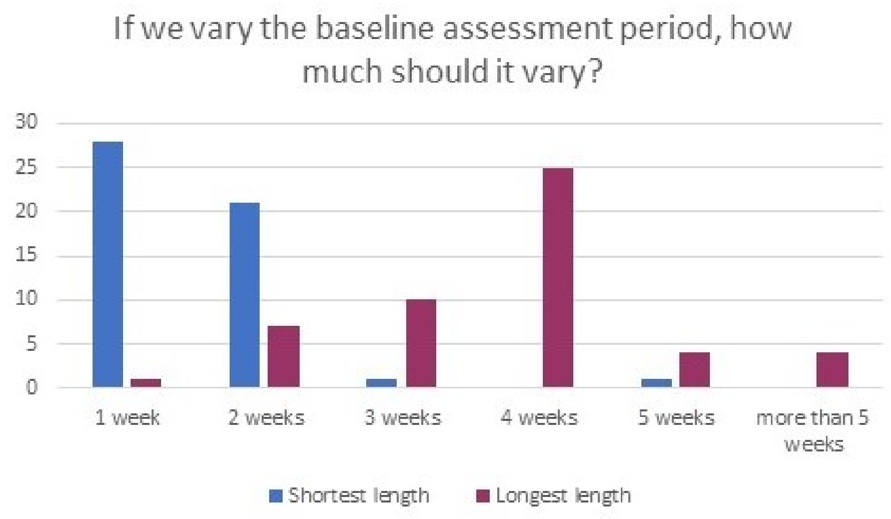

If we have a set length of baseline how long should it be?

◯ 1 week ◯ 2 weeks ◯ 3 weeks ◯ 4 weeks ◯ 5 weeks ◯ more than 5 weeks

Better to vary baseline assessment period depending on presentation — Better to use a set length of baseline assessment period

(Place a mark on the scale above)

If you have any other comments relating to the baseline assessment please write them here:

5) Implementation issues

Intervention reach / can the intervention reach the recipients?

In the service you have mentioned above, what percentage of clients on an average practitioners caseload do you think would be suitable for this intervention (as far as you currently understand the intervention will look)?

0% of caseload 50% of caseload 100% of caseload

(Place a mark on the scale above)

(or other comments about what we have said above.)

Inclusion criteria for your reference (as per section 1).

A 'diagnosis based' intervention
(This might include bipolar affective disorder, severe uni-polar depression, personality disorder)
If more relevant for some mental illnesses than others please describe.

Would the intervention require much modification to be applied to people with different diagnoses?

If you have any other comments about this intervention being diagnosis specific VS being offered to people with other diagnoses or other symptoms (now or in future), please write them here:

Next steps
(response optional)

(response optional)

END OF QUESTIONS

Thank you!

You have completed round 3!

Would you like us to contact you about the results by email?	☐ Please send a summary of the results<br>☐ Please contact me when the results are published<br>☐ Neither of the above
How do you feel about being contacted in future about this research topic?	○ I am keen to be contacted<br>○ I am OK with being contacted<br>○ I prefer not to be contacted
Please select which type of voucher you would like: (You may decline, but we would like to send you this as a small thank you gesture if you will accept.)	○ Amazon (United Kingdom, France, Germany, Italy, Netherlands, Spain, Turkey, Canada, Mexico, United States, Australia, Brazil, China, India, Japan, Singapore)<br>○ IKEA (Australia, Austria, Belgium, Canada, China, Czech republic, Denmark, Finland, France, Germany, Hungary, Italy, Japan, Netherlands, Norway, Poland, Portugal, Russia, Slovakia, Spain, Sweden, Switzerland, United kingdom and USA)<br>○ Prezzee digital gift card (Australia)<br>○ Argos e-gift card (use in store or online) (UK)<br>○ Tesco digital gift card (UK)<br>○ 'Love to shop' voucher (by post, UK)<br>○ Waterstones gift card (by post, UK)
For Amazon or IKEA please specify country and currency:	______________________________
For 'Love to shop' or Waterstones please provide a postal address:	______________________________

____
