# Supplementary material for: A mixed methods expert opinion study on the optimal content and format for an occupational therapy intervention to improve sleep in schizophrenia spectrum disorders
Source: PLoS One. 2022 Jun 6;17(6):e0269453. doi: 10.1371/journal.pone.0269453 (PMC9170103; doi:10.1371/journal.pone.0269453)
Supplement: S5 File — Used in stage 4 with participants with relevant personal experience. (DOCX) [file pone.0269453.s005.docx]

**What aspect of the intervention do you like the sound of the most? (and why)**

**Was there anything described that you would be worried about trying? (and why)**

**Was there much where you thought “I / they already do exactly that” (which things?)**

**What did you think about:**

Avoiding non-sleep activities in bed (except sex)

Reducing excess time in bed & avoiding or reduce napping

Increasing daytime light exposure

light boxes getting outdoor light

Reducing evening light exposure

changing lamps/bulbs amber glasses

Sticking to a regular rise time

Does it seem important to do? Would it be achievable / hard?

Changes to wake up and morning routine

Alarm clocks dawn simulation

Daytime activity (type, timing & amount of activity)

How much help would be needed to change routines?

Evening wind down routine

Do you think this would help?

Any of the other components or elements we haven’t discussed just now?
